# A cortical semantic map integrates fractions and integers in the human brain

**DOI:** 10.1371/journal.pbio.3003820

**Published:** 2026-10-22

**Authors:** Daniela Valério, Samuel Debray, Alireza Karami, Maxime Cauté, Nicolás Gravel, Stanislas Dehaene

**Affiliations:** 1 Université Paris Saclay, INSERM, CEA, Cognitive Neuroimaging Unit, NeuroSpin Center, Gif-sur-Yvette, France; 2 Collège de France, PSL University, Paris, France; University of Surrey Faculty of Health and Medical Sciences, UNITED KINGDOM OF GREAT BRITAIN AND NORTHERN IRELAND

## Abstract

How does the human brain represent the meaning of abstract symbols? Some theories postulate the existence of semantic spaces where concepts occupy positions that reflect their conceptual relationships. In the number domain, psychological evidence suggests that integers are represented along a mental number line which, with education, integrates higher-level number concepts such as fractions. To test this hypothesis, we recorded whole-brain 7T fMRI responses to integer and fraction symbols during an active magnitude comparison task designed to engage magnitude processing. Consistent with predictions, we found both behavioral and neural numerical distance effects. Activation vectors in intraparietal, inferior temporal, prefrontal, hippocampal, and parahippocampal cortices formed a two-dimensional semantic space organized by numerical magnitude and domain (fractions versus integers). Gaussian fits revealed a topographic map of numerical preferences in the anterior inferior parietal cortex, common to both domains. Our results suggest that, when educated adults engage in an explicit magnitude comparison task, their brain can construct an ordered representation that integrates fractions and integers, with a topographic organization in the anterior IPS/IPL.

## Abstract 2

How does the brain represent symbolic integers and fractions? This study shows that educated adults process both integers and fractions within a shared, topographic map in the parietal cortex, indicating that the brain integrates integers and higher-level concepts such as fractions to support the understanding and comparison of numerical magnitudes.

## Introduction

“*God made the integers, all else is the work of Man*” - Leopold Kronecker.

The issue of how the human brain represents the meaning of complex and abstract concepts is a challenging problem. A plausible hypothesis is that concepts are mapped onto internal conceptual spaces or “semantic maps”, in which semantic similarities are represented as distances that can be modeled using multidimensional scaling (MDS; [1]). Within the high-dimensional space provided by the activity of millions of neurons in relevant areas of the brain, each concept would be represented as a specific point or vector, with semantic relationships encoded by the proximity between those vectors, constituting a neural implementation of the concept of second-order isomorphism [2]. This view is supported by the discovery that, in both brain-imaging and multiple single-cell recordings, the major dimensions of semantic variations of words induce gradients of neuronal activation on the surface of the cortex [3–5]. However, while major semantic axes have been found to distinguish broad domains of knowledge, such as animate versus inanimate entities, whether a similar map-like organization exists for abstract concepts *within* a given domain, at the level of individual words, has received fewer attention (though see, e.g., [6–10]).

Here, we tackle this issue by studying a specific domain of semantic competence, central to human education: the cerebral representation of numerical symbols, ranging from simpler numerical concepts such as the integers 0–5 to more abstract forms such as fractions $\frac{\text{1}}{\text{2}}$, $\frac{\text{1}}{\text{3}}$, or $\frac{\text{5}}{\text{2}}$. The ability to perceive numerousness (i.e., the subjective sensation of discrete quantities), estimate concrete numerosities (i.e., the objective number of items in a set), and discriminate numerical ratios emerges early in life [11,12] and is consistently observed across individuals, regardless of formal education [13–16]. Even the magnitude zero, understood as the quantity of a set with no elements, can be grasped by monkeys and humans [17–19]. However, through formal education and the acquisition of symbolic systems, humans move far beyond “quantical cognition” to develop “numerical cognition” [15], expanding the concept of number even beyond integers. While most 7-year-olds struggle with the notion that there might be numbers between 1 and 2, college students find this idea obvious, because education expanded their concept of number to include fractions, decimals, and real numbers. The expansion of the number field, including the invention of negative and complex numbers, represents a uniquely human invention that provides new mathematical tools for modeling the world. Yet, how and where such abstract symbolic mathematical concepts are represented in the brain during a magnitude comparison task, and what kind of semantic space they form, remains poorly understood.

For integers, it is well established that individuals in Western cultures tend to associate smaller numbers with the left side of space and larger numbers with the right, the SNARC effect (Spatial-Numerical Association of Response Codes; [20]). Moreover, two numbers are more easily discriminated as the distance between them increases—a phenomenon known as the distance effect [21,22]. Together, these findings support the idea that integers are mentally represented along a one-dimensional semantic map, a spatial continuum known as the mental number line [20,23]. Constructing this representation requires extracting the quantity associated with a number independently of its format—whether it appears as a word (e.g., “five”), a number (e.g., “5”), or a set of items (e.g., “five dots”). This process is typically attributed to the Approximate Number System (ANS), which is thought to involve the bilateral intraparietal sulci (IPS) as a primary cortical site [17,24–27], although other potential candidates have also been proposed, including inferior temporal cortex and hippocampus [18,28–31]. Interestingly, a similar representation principle may underlie fraction processing, as numerical magnitude (i.e., how large a fraction is) also plays a central role in understanding fractions but see also [32]. Consistent with this idea, a distance effect has been observed during fraction processing in both behavioral [33–36] and brain-imaging studies [37–39], and a SNARC effect has also been found with fractions [40]. Note, however, that an alternative account of the ANS is the Discrete Semantic System (DSS), which explains the distance and SNARC effects in symbolic numbers in terms of the strength of the semantic-memory nodes and their connections, as numbers (e.g., 1 and 2) or concepts (e.g., left and small) that co-occur more frequently tend to develop stronger associations [41–44].

Here, our goal is to understand how symbolic fractions are represented both neurally and behaviorally during a task that elicits magnitude processing, and whether this representation aligns with a mental number line. We hypothesize that, in participants with sufficient education, a number line based on magnitude might integrate the two domains of integers and fractions. This is because, with education and expertise, individuals learn various strategies that help them place numbers correctly along a magnitude continuum [45]. If this is the case, we should expect to observe, at the behavioral level, a distance effect and an internal representational geometry (revealed through MDS) organized according to numerical magnitude, independently of the number’s original domain (integers or fractions). This would be a first source of evidence for a shared number line between fractions and integers. At the neural level, using representational similarity analysis (RSA; [46]), we expect to observe graded BOLD responses such that numbers closer on the mental number line show more similar neural responses. Additionally, if neurons putatively tuned to similar magnitudes cluster together on the surface of the cortex, we expect to find cortical regions where voxels tuned to symbolic numbers are topographically laid out on the cortical surface and present a similar tuning across fractions and integers.

Note that, while previous studies have identified such tunings for nonsymbolic numerosities presented as sets of dots [7,47–49], symbolic tuning for number symbols has only been detected in occipito-temporal regions with functional magnetic resonance imaging (fMRI) [28], and in the mesial temporal lobe using intracranial recordings [18,29,50]. To maximize the chances of observing such tunings, we engaged participants in a demanding continuous number comparison task while collecting individual high-resolution (1.2 mm isotropic) fMRI data at high field (7 Tesla).

To anticipate our findings, we observed graded magnitude-based activation patterns in math-responsive areas as well as the medial temporal lobe. Within the math-responsive areas, numbers with closer magnitudes were represented by more similar activation vectors and in closer spatial proximity. In the bilateral anterior IPS/ inferior parietal lobe (IPL), our images revealed a clear cortical semantic map with a shared magnitude code for both fractions and integers.

## Methods

### Ethics statement

This 7T study was approved by the local ethics committee (Comité de Protection des Personnes Sud-Ouest et Outre-mer III; references: CEA 100 055, ID RCB 2020-A01713-36), and the study was conducted following the Declaration of Helsinki. Written informed consent was obtained from all participants, who were compensated for their participation.

### Participants

Twenty-two healthy adult participants were recruited for this study (13 women and 9 men, age range: 19–39 years, mean age = 26.4, SD = 5.6). All participants were right-handed, native French speakers, and held a high-school degree with a scientific background. They had normal or corrected-to-normal vision and reported no history of psychiatric or neurological disorders.

One participant was excluded due to a cyst in the temporal lobe that could affect that region’s anatomy and functional connectivity. Another participant was excluded after experiencing a panic attack in the scanner and requesting to leave it. Additionally, for a different participant, one of the response buttons failed, preventing analysis of his behavioral responses. As a result, we included 20 participants in the fMRI analyses and 19 in the behavioral analyses.

### Number comparison task

Participants were engaged in a serial continuous comparison task, inspired by Nelli and colleagues [30], in which each target had to be judged as larger or smaller than the previous one. To achieve that, we avoided consecutive numbers with the same magnitude. The targets consisted of 12 numbers: six integers (i.e., 0, 1, 2, 3, 4, and 5), and six fractions ($\frac{\text{1}}{\text{4}}$, $\frac{\text{1}}{\text{3}}$, $\frac{\text{1}}{\text{2}}$, $\frac{\text{2}}{\text{3}}$, $\frac{\text{3}}{\text{2}}$, and $\frac{\text{5}}{\text{2}}$). The stimuli were presented as white Arabic numerals on a black screen for 1 s, with fractions shown using a horizontal line. The small set of familiar fractions was selected to avoid excessive task difficulty and discouragement among participants. Importantly, the fractions were constructed from the same integers, thus allowing us to test whether participants represented magnitude rather than perceptual features (e.g., whether $\frac{\text{1}}{\text{4}}$ is closer to smaller numbers than to 4). All possible transitions between the 12 target numbers occurred an equal number of times, with no number repeated consecutively, resulting in 11 presentations of each target and 132 unique transitions per run. This relatively small stimulus set facilitates interpretable results in MDS and RSA, while ensuring that each number was preceded exactly once by every other number.

Before starting the fMRI experiment, participants completed a few trials to familiarize themselves with the task and stimuli. Each participant was assigned a number based on their arrival order. Participants with an even number were instructed to press the “left button” when the current number was greater than the previous one, and the “right button” when the current number was lower. In the second run, the buttons were reversed. Participants with an odd number started with the opposite button assignments. Each participant completed two runs, each lasting 9 min and 42 s. After the number presentation, a pseudo-randomly jittered interval of 2–4 s followed, fixed by the experimenter to ensure a mean SOA of 4 s, during which the fixation cross turned green if the response was recorded. Subsequently, the fixation cross turned red 500 ms before the onset of the next stimulus to prepare participants (see Fig 1A). Participants received a 10 s rest time for every 45 stimuli, the fixation cross was on the screen the entire time, and the feedback with the percentage of correct answers appeared for 3 s. Before starting the next block, the fixation cross turned red to prepare participants. At the beginning of each block, the last number before the rest was repeated when participants returned to the task.

**Fig 1:**
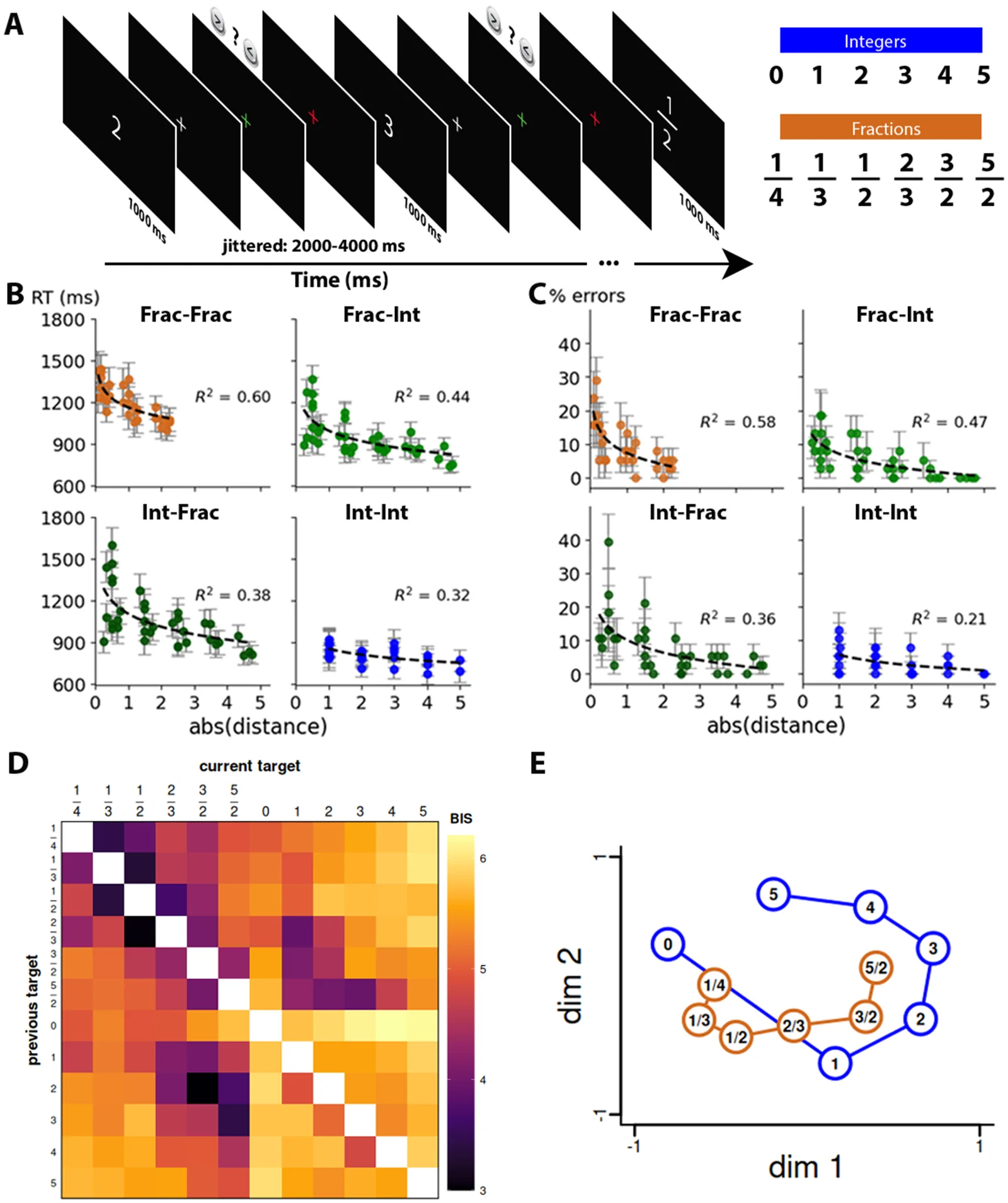
Experimental design and behavioral results. **A)** An example of the task sequence in the scanner: participants compared the current number to the previous one. Each number was followed by a fixation cross that turned green when the response was registered and red 500 ms before the next trial to prepare participants. After 45 stimuli, participants received feedback on their percentage of correct answers. Right of panel A, the full set of stimuli is shown, including both integers and fractions. **B)** RTs as a function of absolute distance between current and previous numbers (132 combinations). Panels separate trials in which both the previous and the current numbers were integers (int), both were fractions (frac), and trials with mixed number domains. **C)** Percentage of errors as a function of the absolute distance between current and previous numbers (same format). **D)** Balanced Integration Score (BIS), where smaller values (closer to purple) indicate greater difficulty in comparing numbers, while larger values (yellow) indicate easier comparisons. **E)** Two-dimensional MDS representation of the BIS matrix, showing that fractions and integers are organized according to their magnitudes. The data underlying this Figure can be found in https://doi.org/10.17605/OSF.IO/JQCNA.

### Math localizer

To localize math-responsive areas within each individual, we used a highly reproducible sentence-based paradigm previously employed multiple times in our lab [51–55], which has the advantage of not including symbolic numbers in Arabic format. Participants read 60 sentences and had to decide whether they were True or False. Half of the sentences were mathematical statements, either stating arithmetic calculations in verbal form or describing geometrical facts concerning basic shapes (e.g., “*Number three multiplied by twelve is thirty-six*” or *“A rectangle has only one axis of symmetry”*). The remaining sentences were nonmathematical, including general factual knowledge or sentences referring to a person’s perspective (e.g., “*The camel is a large humpbacked mammal” or “For Einstein the atomic bomb is a harmless object”*). In each category, half of the sentences were true (T), and the other half were false (F), with the response buttons (left and right) counterbalanced across participants. Participants completed a single run lasting ~11 min.

The visual stimuli were balanced to control the number of words, total number of characters, word length, and frequency of occurrence in French. They were presented using rapid serial visual presentation (RSVP) at 350 ms per word, with an average total duration of 3.2 s. Each sentence was followed by a fixation cross, jittered to maintain an average stimulus onset asynchrony (SOA) of 8 s. A red cross was presented 350 ms in advance of the upcoming stimulus. Participants were instructed to answer as quickly and as accurately as possible, and response times were measured relative to the onset of the last word.

Stimulus delivery and response collection of both Localizer and Numbers tasks were controlled by Expyriment [56].

### fMRI acquisition

MRI data were acquired using a 7T MAGNETOM whole-body MR scanner (Siemens Healthineers, Erlangen, Germany) with a 32-channel head coil (Nova Medical, Wilmington, USA) at the NeuroSpin Center of the French Alternative Energies and Atomic Energy Commission. Structural MRI data were collected using T1-weighted rapid gradient echo (MPRAGE) sequence (repetition time (TR) = 5,000 ms, echo time (TE) = 2.51 ms, resolution = 0.65 mm isotropic, field of view (FoV) = 208 ⨯ 208 mm2, flip angle = 5/3, bandwidth (BW) = 250 Hz/px, echo spacing = 7 ms). Functional MRI (fMRI) data were acquired using a T2*- weighted gradient echo planar imaging (EPI) sequence (TR = 2,000 ms, TE = 21 ms, voxel size = 1.2 mm isotropic, multiband acceleration factor = 2, FoV = 192 ⨯ 192 mm2, flip angle = 75, BW = 1,488 Hz/px, partial Fourier = 6/8, echo spacing = 0.78 ms, number of slices = 70 (no gap)). To correct for EPI distortion, a five-volume functional run with the same parameters except for the opposite phase encoding direction (posterior to anterior) was acquired immediately before each task run. Manual interactive shimming of the B0 field was performed for all participants. The system voltage was set at 250 V for all sessions, and the fat suppression was decreased per run to ensure that the specific absorption rate did not surpass 62% for all functional runs.

### Anatomical and functional preprocessing

Both anatomical and functional MRI data were preprocessed using fMRIPrep 20.0.2. This pipeline incorporates standard preprocessing steps within a robust and user-friendly workflow, ensuring reliable performance across datasets independently of the data idiosyncrasies and highly consistent results [57].

The T1-weighted (T1w) anatomical image was first corrected for intensity nonuniformity (INU) with N4BiasFieldCorrection [58], distributed with ANTs 2.3.3 [59], RRID: SCR_004757), and used as T1w-reference throughout the workflow. Next, the T1w-reference was then skull-stripped with a *Nipype* implementation of the ANTs antsBrainExtraction.sh workflow, with the OASIS30ANTs template as the target. Brain tissue segmentation into cerebrospinal fluid (CSF), white matter (WM), and gray matter (GM) was performed on the skull-stripped T1w image using fast from FSL [60]. Cortical surface reconstruction was carried out using recon-all from FreeSurfer 7.2.0 [61], and the resulting brain mask was subsequently refined with a custom variation of the Mindboggle method [62]. Finally, volume-based spatial normalization to the MNI152NLin2009cAsym standard space was performed through nonlinear registration with antsRegistration (ANTs 2.3.3), using brain-extracted versions of both the T1w reference and the T1w template.

A reference volume and its skull-stripped version were generated by aligning and averaging a single-band reference (SBRefs). Head-motion parameters relative to the BOLD reference image, including transformation matrices and the six corresponding rotation and translation parameters, were estimated before any spatiotemporal filtering using MCFLIRT (FSL 6.0.5; [63]). The estimated *fieldmap* was then aligned to the target echo-planar imaging (EPI) reference run. The resulting field coefficients were then mapped onto the reference EPI using the computed transformation. BOLD runs were corrected for slice-time differences using 3dTshift from AFNI [64], with all slices temporally realigned to 0.975 s, corresponding to the midpoint of the slice acquisition interval (0–1.95 s). The BOLD reference image was then co-registered to the T1w anatomical reference using bbregister (FreeSurfer), which implements boundary-based registration [65]. Co-registration was performed with nine degrees of freedom to account for residual distortions present in the BOLD reference image. A reference volume and its skull-stripped version were first generated using a custom methodology implemented in *fMRIPrep*. Several confounding time series were then calculated from the *preprocessed BOLD* data, including framewise displacement (FD), DVARS, and three region-wise global signals. FD was calculated using two formulations: the absolute sum of relative motions described by [66] and the relative root mean square displacement between affine transformations proposed by [63]. Both FD and DVARS were computed for each functional run using their implementations in *Nipype,* following the definitions by [66]. Global signal regressors were extracted from the CSF, the WM, and the whole-brain masks. In addition, physiological noise regressors were estimated using component-based noise correction method (*CompCor*, [67]). Principal components were derived from the *preprocessed BOLD* time series after high-pass filtering with a discrete cosine basis set using a 128 s cut-off period. Two *CompCor* variants were implemented: temporal (tCompCor) and anatomical (aCompCor). For tCompCor, principal components were calculated from the top 2% variable voxels within the brain mask. For aCompCor, probabilistic masks corresponding to CSF, WM, and combined CSF + WM compartments were generated in anatomical space. This mask was obtained by dilating a GM mask extracted from the FreeSurfer’s *aseg* segmentation, and it ensures components are not extracted from voxels containing a minimal fraction of GM. Finally, these masks were resampled into BOLD space and binarized using a threshold of 0.99. Components were also calculated separately within the WM and CSF masks. For each CompCor decomposition, the *k* components with the largest singular values were retained, such that the selected components’ time series explained at least 50% of the variance within the corresponding nuisance mask (CSF, WM, combined, or temporal mask). Components that did not contribute to this threshold were discarded. The head-motion estimates obtained during the correction step were included in the corresponding confounds file. To further model nuisance-related variance, the confound time series derived from head motion estimates and global signals were expanded by including temporal derivatives and quadratic terms [68]. Volumes with FD greater than 0.5 mm or standardized DVARS exceeding 1.5 were identified as motion outliers. Additional nuisance time series are calculated using principal components analysis of the signal found within a thin band (*crown*) of voxels around the edge of the brain, following the approach proposed by [69]. The BOLD time series were resampled into standard space, generating a *preprocessed BOLD run in MNI152NLin2009cAsym space*. First, a reference volume and its skull-stripped version were generated using a custom procedure implemented in *fMRIPrep*. All resampling can be performed with *a single interpolation step* by composing all the pertinent transformations, including head-motion transform matrices, susceptibility distortion correction (when available), and co-registration transforms between functional, anatomical, and output spaces. Volumetric resampling were performed using antsApplyTransforms (ANTs), employing Lanczos interpolation to reduce the smoothing effects commonly associated with other interpolation kernels [70]. Surface-based resampling was carried out using mri_vol2surf from FreeSurfer.

### Data analysis

We analyzed behavioral data by integrating response times and error rates into a Balanced Integration Score (BIS; [71]), separately for each combination of the 12 numbers, and as a function of the numerical distance between the current and previous number. To ensure that all distance values were nonnegative, we subtracted, for each participant, the minimum BIS value from all cells in the matrix. To the BIS values, we fitted a linear mixed-effects model in which trial type (fraction versus integer domain for both the current and the previous stimuli) and numerical distance (the log of the absolute difference between current and previous numbers) were included as interacting fixed effects. To account for repeated measures and individual differences in baseline performance, participant was included as a random effect.

Statistical analyses of the fMRI data were performed using NiLearn (RRID: SCR_001362) and rsatoolbox [72]. In the general linear models (GLM), regressors were convolved with the canonical hemodynamic response function (HRF). GLM1 included 17 regressors of interest: one for each current target number (12 regressors); two for left and right button presses; one for RT difficulty, calculated as the current RT minus the participant’s average RT; one for feedback onset; and one for the first target presentation, where participants were not required to respond. Additionally, to regress out motion and physiological noise, we included six head motion parameters, the CSF and white matter signal, 10 anatomical CompCor (computed by *fMRIPrep*), and 14 cosine drifts. This GLM was used for the PCA, RSA, searchlight, MDS, and Gaussian fit analyses. The data remained unsmoothed for all analyses, but for visualization purposes, we smoothed the data at the group level with a 6 mm kernel. We chose to use unsmoothed data to preserve sensitivity to subtle patterns and fine-grained distinctions between numbers. An exception was made for the Gaussian fit analysis, where the data were smoothed at the individual level with a 4 mm kernel as well as the results of the group-averaged-data. Possibly due to a limited amount of data, smoothing was necessary, but the results remained when using either 2.4 mm or 4 mm smoothing.

GLM2 was very similar to GLM1, except that we excluded the RT difficulty regressor and included the logarithm of the absolute distance, calculated as log(|current number − previous number|). This model was used exclusively for Fig 2C, and the data were smoothed at the group level with a 6 mm kernel.

**Fig 2:**
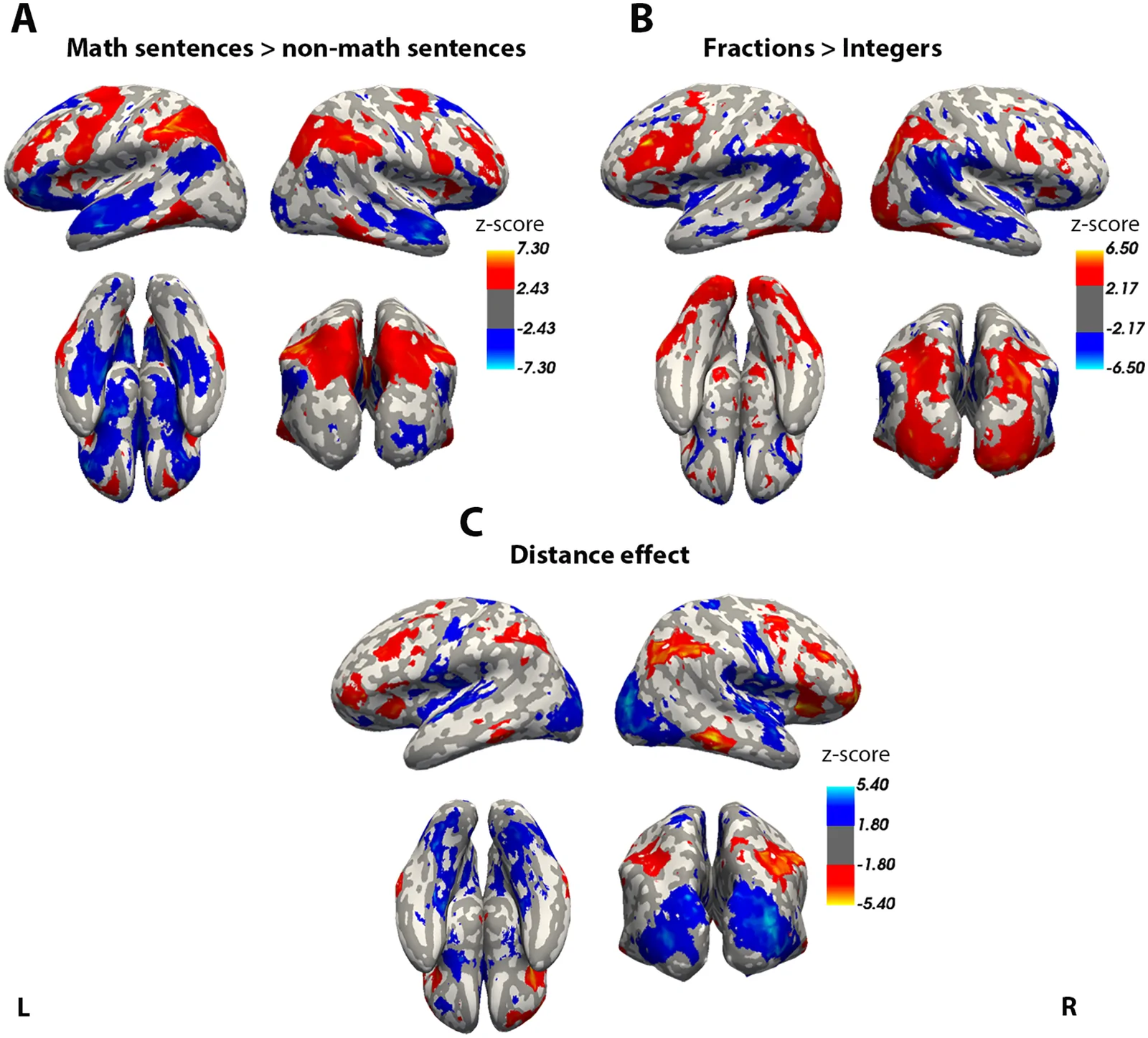
A math-responsive cortical network and its response to three distinct univariate contrasts. **A)** Greater activation for math than for nonmath sentences in the localizer. **B)** Greater activation for fractions than for integers. **C)** Effect of the distance between the current and previous numbers (greater activation to closer numbers shown in red). All maps thresholded at a false discovery rate (FDR) *q* < 0.05 across the whole-brain.

### Principal component analysis (PCA)

We used PCA on the 12 target-related voxel-wise model weights to identify the most important orthogonal dimensions for the task. We averaged the t-maps for each target number across participants using unsmoothed data (similar results were obtained at the individual level in the majority of participants). Then, we created a matrix where each row corresponded to a target number, and each column corresponded to a voxel. We applied PCA to these weights. We determined that two components were optimal by examining the elbow of the Scree Plot. To project the results onto the cortical surface of a common space across participants, we selected the top 1% of voxels, and this data was subsequently smoothed with a 6 mm kernel. This threshold allowed us to isolate the voxels with the most robust and reliable response to each component, whereas more lenient thresholds preserved the results but extended the coverage to a larger portion of the brain.

### Representational similarity analysis (RSA)

We identified brain areas showing greater activation for math-related sentences compared to nonmath-related sentences in the localizer task, at the whole-brain level for each participant (each thresholded at *p* < 0.001 uncorrected). From the contrast math > nonmath sentences, we also manually selected the parietal, frontal, and temporal ROIs at the individual level. BOLD representational dissimilarity matrices (RDMs) were constructed by calculating the correlation distance d = 1−r, where r is the Pearson correlation coefficient between the within-subject multivoxel patterns elicited by each number, resulting in a 12 x 12 RDM for each participant, which was then averaged across participants. To visualize neural geometry, we used a two-dimensional metric inter-individual difference scaling (INDSCAL) MDS model, which takes into account the individual participants’ RDMs, implemented using the R-package SMACOF [73,74].

To identify cortical sectors in which the neural RDM correlated with a specific RDM—particularly focusing on the distance across the domains of fractions and integers—while accounting for the influence of remaining RDMs, we performed a multiple-regression searchlight RSA. This analysis was performed within a mask of mathematically responsive areas (math > nonmath sentences, FDR *q* < 0.05) as defined by the localizer, to which we added Glasser-defined ROIs of hippocampi and parahippocampal cortex. The neural RDM for each searchlight was computed using correlation distances. For the searchlight analysis, we included all voxels within a radius of 4 mm of the central voxel. For each searchlight sphere, we computed neural RDMs from the condition-by-voxel matrix of estimated neural responses using Pearson correlation distance between pairs of conditions. This procedure was carried out at the individual participant level, followed by a second-level analysis across participants, using FDR correction *q* < 0.001 for multiple comparisons. The multiple regression model included five predictor RDMs: distance within fractions, category of fractions, distance within integers, category of integers, and distance across the domains of integers and fractions.

### Voxel tuning

For this analysis, we averaged the t-maps for each target number across participants using smoothed data (4 mm FWHM). The same analysis was also performed at the individual level in each participant’s native space. We then created a matrix in which each column represented a target *t*-value and each row corresponded to a voxel within the mask of math-related regions defined by the localizer math > nonmath sentences, FDR *q* < 0.05, as well as the Glasser ROIs of IPS, pITG, MFG (see S4 Fig), parahippocampal, and hippocampus. Separate analyses were conducted for integers and fractions. Each voxel’s values were normalized between their minimum and maximum across conditions of integers or fractions, resulting in a range from 0 to 1. A Gaussian function was then fitted using the Levenberg–Marquardt method within a bounded parameter space, yielding for each voxel a preferred value on a continuous scale from 0 to 5 for integers and from 0.25 to 2.5 for fractions. The amplitude was constrained between 0 and 1, and the width between 0.1 and 2. At the single-subject level, maps were thresholded at R^2^ > 0.3 because this threshold allowed us to reliably inspect maps across the 20 participants while ensuring consistency. Similar thresholds have been used in pRF analysis at the single-participant level, even after averaging data across multiple sessions (e.g.[7,75]). In contrast, group-averaged data provides a significantly higher signal-to-noise ratio, allowing us to use a stricter threshold of R^2^ > 0.5. The same threshold was applied both to individual runs and to the average of the two runs.

Using data from one of the runs (1 or 2), we categorized voxels into 6 bins by rounding their preferred values for integers and, for fractions, by applying category boundaries based on the subtraction (next fraction - previous fraction)/2. Then, we plotted the normalized activation using data from the other run and averaged the two resulting plots (finding preferred numbers with run 1 and plotting the activation of run 2, and vice versa). To assess statistical significance, we organized the average data in a matrix (6 x 6 for integers, 6 x 6 for fractions) where columns indicated the preferred numbers and rows the average normalized activation to each target number. Then, we tested whether the diagonal elements of this matrix were significantly larger than the off-diagonal elements. For that, we shuffled the columns to break the correspondence between rows and columns and computed a permutation test (*n* = 720 permutations).

We correlated each voxel’s preferred value for fractions and for integers within math-responsive areas (pITG, MFG, and IPS) and in aIPS/IPL (Glasser ROIs: PF and IP2). To assess the significance of these correlations, we generated 10,000 random white-noise brain volumes, smoothed them with a 4 mm FWHM kernel to match the spatial smoothness of our real data, and extracted the corresponding voxels to each ROI. For each ROI, we computed the Pearson correlation between the fraction map and each smoothed noise map, generating a null distribution of correlation values.

Statistical significance was then calculated by comparing the observed Pearson correlation against this spatially preserved null distribution. This allowed us to test whether the observed correlation between fractions and integers was greater than chance while accounting for the spatial smoothness of the data. We then applied the same procedure to assess the correlation between the two runs for integers and fractions. To plot the tuning data across fractions and integers, we selected the voxels corresponding to numbers from 0 to 3, as there were too few voxels representing magnitudes of 4 and 5. Voxels were assigned to each category based on their rounded preferred magnitude (e.g., voxels categorized as 0 had preferred values between 0 and 0.49). We then extracted the normalized activation for fractions from these voxels (see S7 Fig).

To control for the cumulative risk of false positives across ROIs, each permutation analysis was corrected for multiple comparisons across ROIs using the Benjamini–Hochberg FDR test.

## Results

### Behavioral evidence for a distance effect and a mental number line

Participants responded significantly more slowly to fractions (M_Frac-Frac_ = 1,192 ms; SD_Frac-Frac_ = 129) than to integers (M_Int-Int_ = 808 ms; SD_Int-Int_ = 64; *t*(28) = −17.12, *p* < 0.001), and they made more errors when both the previous and current numbers were fractions (M_Frac-Frac_ = 9.5%; SD_Frac-Frac_ = 6.90) than when both were integers (M_Int-Int_ = 3.7%, SD _Int-Int_ = 3.56; *Mann-Whitney U = 368*, z = 3.77, *p* < 0.001). In trials requiring comparisons between integers and fractions, participants were also slower when integers preceded fractions (M_Int-Frac_ = 1,053 ms; SD_Int-Frac_ = 191) than when fractions preceded integers (M_Frac-Int_ = 952 ms; SD_Frac-Int_ = 147, *Mann*−*Whitney U = 469*, z = 2.14, *p* = 0.03), with no corresponding differences in accuracy between the two conditions (M_Int-Frac_ = 8.11% of errors; SD_Int-Frac_ = 8.11; M_Frac-Int_ = 5.56% of errors; SD_Frac-Int_ = 5.40; *Mann−Whitney U = 329*, z = 1.23, *p* = 0.22). These results were stable across time, since reaction times were highly correlated between run 1 and run 2 (*r* = 0.95, *p* < 0.0001), as were the percentage of errors (*rho* = 0.49, *p* < 0.0001). Overall, these results indicate that participants found conditions involving fractions more difficult than those involving integers.

RTs and the percentage of errors are presented as a function of absolute distance in Fig 1B and 1C for each condition (i.e., Frac-Frac, Frac-Int, Int-Frac, and Int-Int). Regardless of condition, we found a statistically significant distance effect: participants were slower and less accurate when two numbers were closer on the number line. This effect was particularly pronounced when both the current and previous numbers were fractions (RTs: β_Frac-Frac_ = −220.26, *t*(28) = −6.54, *p* < 0.0001; Errors: β_Frac-Frac_ = −11.54, *t*(28) = −6.26, *p* < 0.0001; βs ob*t*ained with a linear regression model with log-transformed numerical distance as predictor), but a distance effect was also present when both numbers were integers (RTs: β_Int-Int_ = −146.68, *t*(28) = −3.60, *p* = 0.001; Errors: β_Int-Int_ = −6.65, *t*(28) = −2.75, *p* = 0.01) or when participan*t*s compared an integer to a previous fraction (RTs: β_Frac-Int_ = −252.01, *t*(34) = −5.12, *p* < 0.0001; Errors: β_Frac-Int_ = −9.69, *t*(34) = −5.54, *p* < 0.0001) and vice-versa (RTs: β_Int-Frac_ = −308.79, *t*(34) = −4.61, *p* < 0.0001; Errors: β_Int-Frac_ = −12.67, *t*(34) = −4.37, *p* < 0.0001).

To combine information from RTs and error rates, we calculated the BIS score for each pair of numbers [71]; see Fig 1D; see S1 Fig for an alternative measure, Efficiency Score). Values closer to 0 indicate more difficult trials. A participant level linear mixed-effects model revealed a logarithmic distance effect between the previous and current numbers (β = 0.79, *t*(2447) = 3.77, *p* = 0.0002). Additionally, the BIS score decreased when the *p*revious item was a fraction compared to an integer (β = −0.38, *t*(2447) = −4, *p* < 0.0001), and also when the current item was a fraction com*p*ared to an integer (β = −0.68, *t*(2447) = −7.04, *p* < 0.0001). The only s*t*atistically significant interaction was between the *p*revious and current number conditions, indicating further difficulty when both items were fractions (β = 0.27, *t*(2447) = 2.17, *p* = 0.03). Model com*p*arison confirmed tha*t* numerical distance was best modeled by the logarithm of numerical distance rather than by a linear function (Δ AIC = 6), reflecting a classical tapering of the distance effect when distance increases.

MDS of the balanced integration score (Fig 1E) showed a one-dimensional curvilinear structure (number line) embedded in two dimensions, similar to that previously observed in similar magnitude comparison tasks [30,76]. Importantly, fractions and integers were integrated together, and both were ordered according to their magnitudes on the first dimension (*x* axis), with smaller numbers on the left side and larger numbers on the right side. When we correlated x-coordinates of the MDS solution with numerical magnitude using a Spearman correlation (to account for curved or otherwise nonlinear representations), we found a statistically significant correlation (rho(10) = 0.75, *p* = 0.007). The second dimension (*y* axis) seemed to encode difficulty, as it separated the intrinsically easier numbers 0 and 5 (because they lie at the extremes) from other numbers. To obtain a measure of difficulty, we calculated the average value of each column in the behavioral BIS matrix, where higher BIS values indicate easier comparisons. A spearman correlation between y-coordinates of the MDS solution and this difficulty measure revealed a significant relationship (rho(10) = 0.64, *p* = 0.03). Similar results, but perhaps noisier, were found with the Efficiency score (S1 Fig). When we recomputed the MDS excluding 0 and 5, their number line organization was largely unchanged (S2 Fig), further supporting the role of magnitude as a major organizing dimension of semantic space.

### Univariate fMRI activations converge to a classic network of math-responsive areas

Three univariate contrasts converged to a largely common set of classic math-responsive regions (Fig 2). First, a contrast for greater activation to math-related sentences than to nonmath-related sentences in the localizer revealed an extensive bilateral network, including the full length of the IPS, posterior inferior temporal gyri (pITG), precentral gyri, middle frontal gyri (MFG), and precuneus. The opposite contrast (in blue), indicating a greater response to nonmath-related sentences, activated a classic and bilateral network of semantic regions, comprising the angular gyri, superior and middle temporal gyri, temporal poles, fusiform gyri, superior and inferior frontal gyri, and posterior cingulate (see Fig 2A). Second, in the numbers task, most of the above math-responsive network showed a greater activation for fractions compared to integers in bilateral regions, including the IPS, precentral gyri, inferior and middle frontal gyri, precuneus, pITG, now also extending to lateral fusiform and lateral occipital cortex (presumably due to the higher visual complexity of fractions; see Fig 2B). Third, similar regions, including bilateral IPS and pITG showed a distance effect during number comparison, i.e., a parametric modulation by the log of the absolute distance between the current and previous numbers, with greater activation to smaller distances, as previously described (e.g., [26], see Fig 2C).

### Vector-based analyses of the neural representation of numbers

Using a GLM with one regressor per target, we obtained, within each participant, a reliable high-resolution estimate of the fMRI activation pattern or “vector” evoked by each of 12 individual number concepts. Our aim was to probe whether these vectors organized as a semantic map and what were its main axes of variations.

As a first step, we performed a whole-brain principal component analysis (PCA) of the average vectors across participants within a standard anatomical space (MNI). PCA revealed two main orthogonal components that explained most of the variance in our data: PC1 reflected a categorical distinction between fractions and integers, while PC2 reflected a near-perfect ordering by number magnitude, from small to large, distinguishing smaller and larger numbers while pooling together fractions and integers (Fig 3). In individual analyses, the first component was observed in 14 of our 20 participants, whereas the second was present in 13. Each component produced distinct spatial maps, although some parietal regions were involved in more than one component. Specifically, PC1 confirmed the univariate analysis of Fig 2B, indicated that fractions (probably because of their greater visual and/or semantic complexity), mobilized bilateral occipital, pITG, posterior IPS, and MFG. Crucially, PC2 revealed that a magnitude map mixing fractions and integers was present in much of the math-responsive network (Fig 3B, bottom), with a particularly strong small-to-large gradient in the anterior IPS (extending into the inferior parietal lobule for small numbers), with additional contributions of the MFG, pITG, posterior cingulate, parahippocampal cortex and hippocampus. Together, PC1 and PC2 explained 36.23% of the variance. To determine the unique contribution of domain (fractions versus integers) and numerical magnitude on the brain, we conducted separate multiple linear regressions for the first and second principal components. For the first principal component, the model fit was highly significant (*F*(2, 9) = 25.47, *p* = 0.0002, R^2^ = 0.82), and domain was a significant predictor (*b* = 132.42, *t*(9) = 6, *p* = 0.0002), whereas magnitude was not (*p* = 0.74). For the second principal component, the model fit was also significant (*F*(2, 9) = 11.98, *p* = 0.003, R^2^ = 0.67) but revealed the opposite effect: magnitude significantly predicted the second component (*b* = −28.17, *t*(9) = −4.82, *p* = 0.0009), whereas domain did not (*p* = 0.13). Thus, this analysis confirms that number magnitude is a major determinant of number representations, which affects a broad set of brain areas. Importantly, these findings remained unchanged when the analysis was repeated after excluding the larger integers 4 and 5, so that integers and fractions covered a similar numerical range (see S3 Fig).

**Fig 3:**
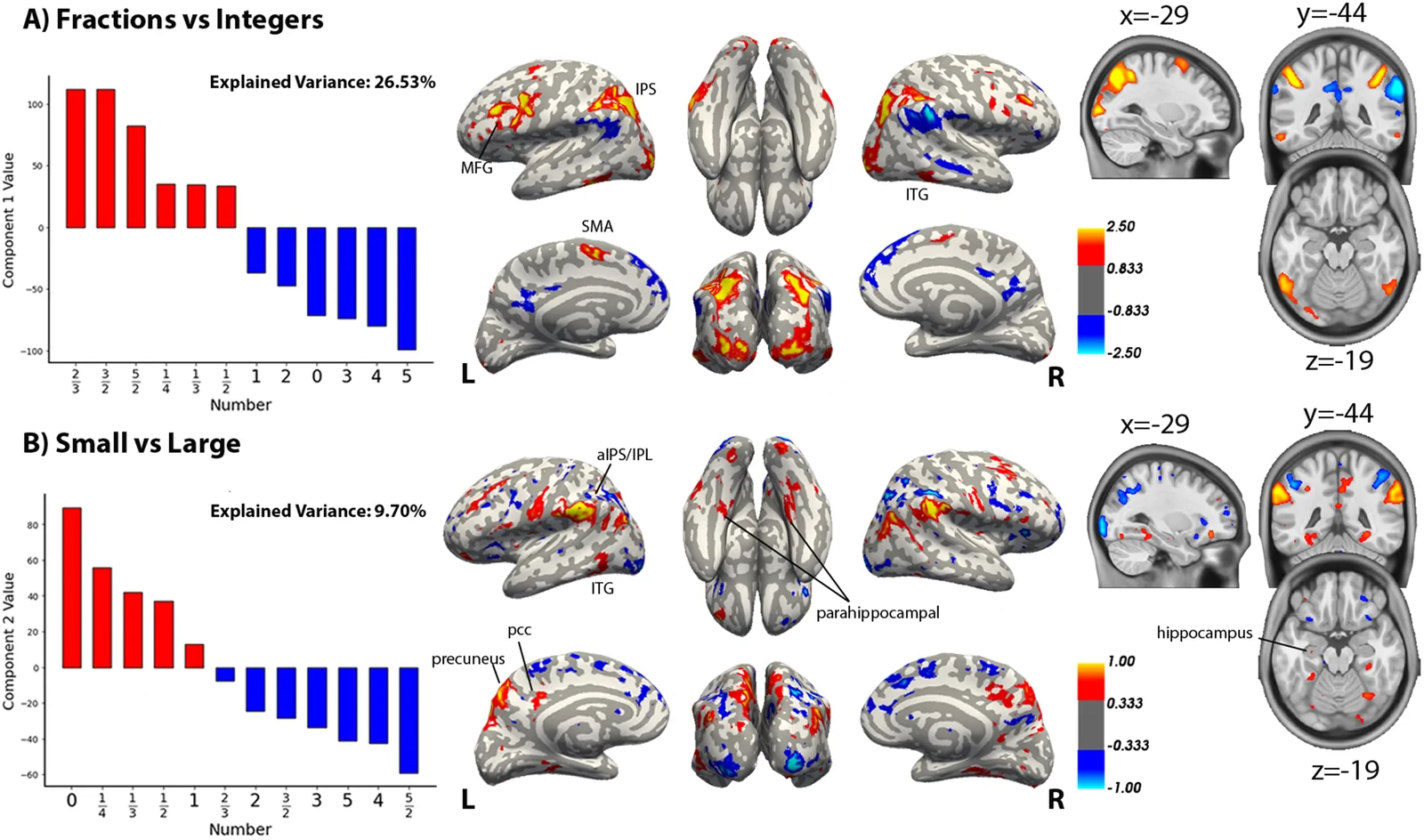
Whole-brain principal Component Analysis (PCA) reveals the two main dimensions of number representations: domain and magnitude. In this purely data-driven analysis of the activation vectors evoked by each of the 12 number targets, PC1 (top) distinguished between fractions and integers, while PC2 ordered numbers almost perfectly according to their magnitude. In each case, the 12 loadings (ordered) and the corresponding voxel weights are shown, in surface and slice views. The data underlying this Figure can be found in https://doi.org/10.17605/OSF.IO/JQCNA. Abbreviations: SFG: superior frontal gyrus; MFG: middle frontal gyrus; IFG: inferior frontal gyrus; SMA: supplementary motor area; PCC: posterior cingulate cortex; ITG: inferior temporal gyrus; MTG: middle temporal gyrus.

To take advantage of the high-resolution individual activations available using 7T fMRI, we extracted single-participant vectors within the entire math-responsive network by first identifying subject-specific voxels showing greater activation to math than to nonmath sentences in the localizer (*p* < 0.001 uncorrected).

Next, we focused on the six main areas identified by Amalric and colleagues [53] (left and right IPS, pITG, and MFG). In those voxels, we extracted the activation vectors to each of the 12 target numbers in the main comparison task, then visualized those vector spaces using RDMs followed by MDS.

This analysis confirmed that neural vector spaces were organized by two dimensions: a main distinction between fractions and integers, and a gradient of continuous magnitude (Fig 4). The latter was seen in two ways: in the RDMs, neural vectors became increasingly similar as the numbers were closer, i.e., for cells closer to the diagonal of the matrix (for both integers and fractions); and in the 2-dimensional MDS solutions of those RDMs, integers and fractions, while separated, were similarly ordered by magnitude. Furthermore, information was represented similarly across the three math-responsive regions IPS, pITG, and MFG.

**Fig 4:**
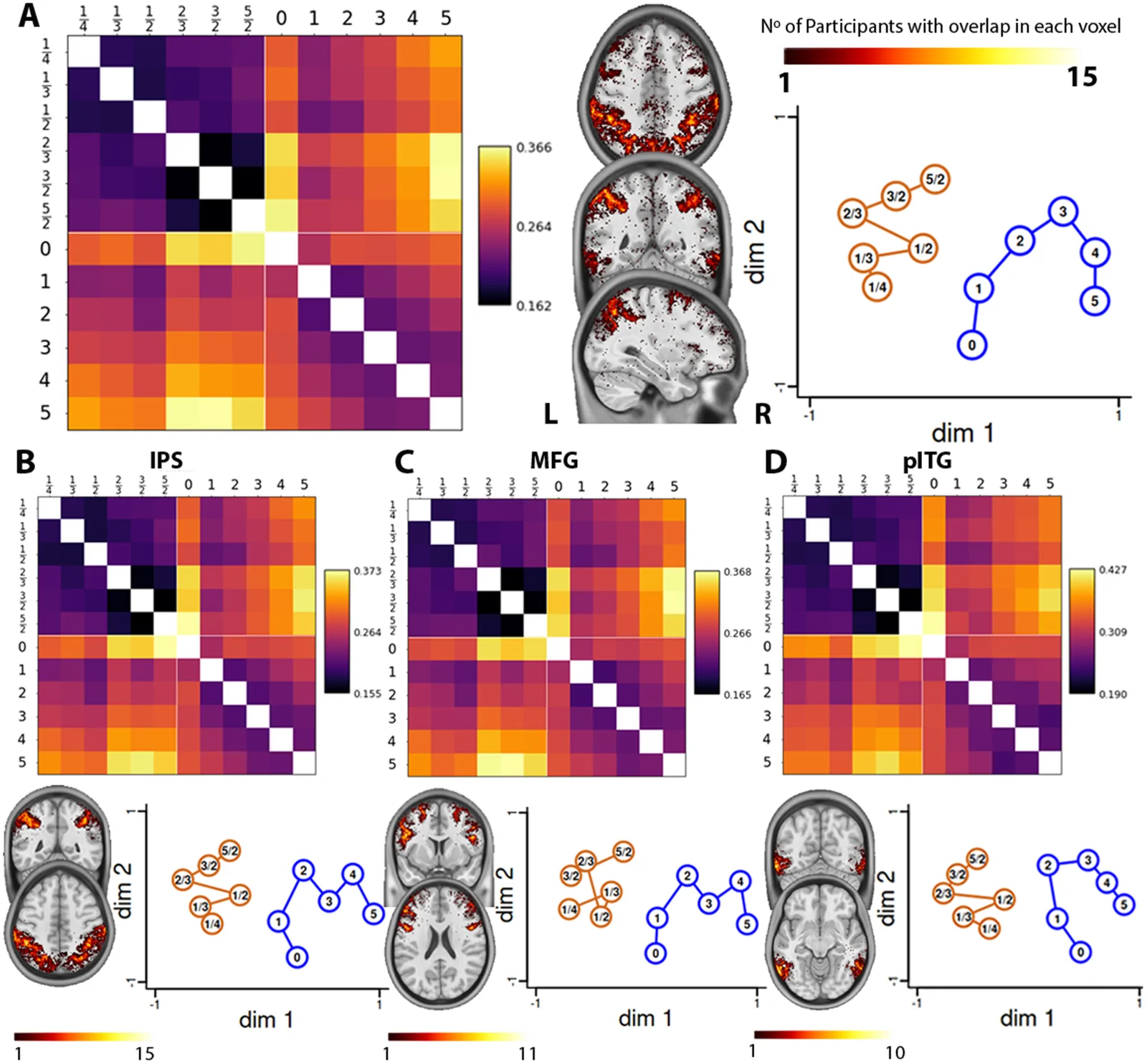
Visualization of the neural representational spaces for numbers within math-responsive areas. Representational similarity matrices and their 2-dimensional MDS solutions were used to visualize the 12-dimensional vector space spanned by number activations within individual voxels-of-interest identified by the math>nonmath contrast in the sentence localizer. **A)** On the left, the RDM shows the average dissimilarity across participants. In the middle, voxel overlap across individuals is displayed with lighter colors indicating higher overlap (up to a maximum of 15 participants). On the right is the corresponding neural geometry representation derived from MDS. **B)** The top panel shows the average RDM restricted to the intraparietal sulcus (IPS) regions of interest (ROIs); the bottom panel shows participant overlap and the resulting neural geometry. **C)** Average RDM and MDS for middle frontal gyrus (MFG) ROIs. **D)** Average RDM and MDS for posterior inferior temporal gyrus (pITG) ROIs. The data underlying this Figure can be found in https://doi.org/10.17605/OSF.IO/JQCNA.

Importantly, these results remained stable even when the analysis was restricted to within-domain trials (i.e., integers preceded by integers, and fractions preceded by fractions; see S5 Fig). Moreover, this separation allowed us to assess the reliability of our brain-activity measures: there was a high correlation between the brain activity patterns measured on within-domain trials and on cross-domain trials (see S6 Fig).

Since the hippocampus and parahippocampal cortex were also identified in the PCA, and since the human hippocampus and mesial temporal lobe have been shown to contain number-tuned neurons [18,29,50], we also analyzed vector spaces in those regions. We used the bilateral ROIs defined by [77] and extracted the entire hippocampus and parahippocampal cortex. In these regions, an organization according to magnitude was even more prominent, with MDS dimension 1 ordering the targets according to their approximate magnitude regardless of whether they were fractions or integers (although a distinction between the two domains remained visible in MDS dimension 2). Note that, similar to the behavioral data, the integer number line seemed to bend after three in all MDS representations, which may reflect the absence of fractions in this range (see Fig 5). Importantly, this effect was not driven solely by the presence of “easy” extreme numbers, as the pattern remained consistent when we recomputed the MDS excluding the extremes 0 and 5 (S2 Fig).

To explicitly test for a shared representation of fractions and integers, we modeled the RDMs using multiple regression analysis focusing on the distance between fractions and integers, but also including 4 regressors controlling for categorical effects and for within-domain distance effects (Fig 6, top). In each region of the math-responsive network (i.e., IPS, ITG, and MFG) as well as medial temporal lobes, a significant effect of fraction-to-integer distance on neural dissimilarity was found (S1 Table). Thus, math-responsive regions were not only organized according to within-domain distances between two integers or two fractions (e.g., $\frac{\text{1}}{\text{4}}$ is more similar to $\frac{\text{1}}{\text{3}}$ than to $\frac{\text{5}}{\text{2}}$), but also by the between-domain distances (e.g., $\frac{\text{1}}{\text{4}}$ is more similar to 0 than to 4), indicating that in our educated adult participant, the magnitude of fractions was well integrated with that of integers. A searchlight RSA analysis within the math-responsive areas (Fig 6, bottom) further showed that the regions sensitive to cross-domain distances included the bilateral hippocampus and parahippocampal cortex, IPS, pITG, MFG as well as precentral cortex, posterior cingulate cortex, and precuneus.

**Fig 5:**
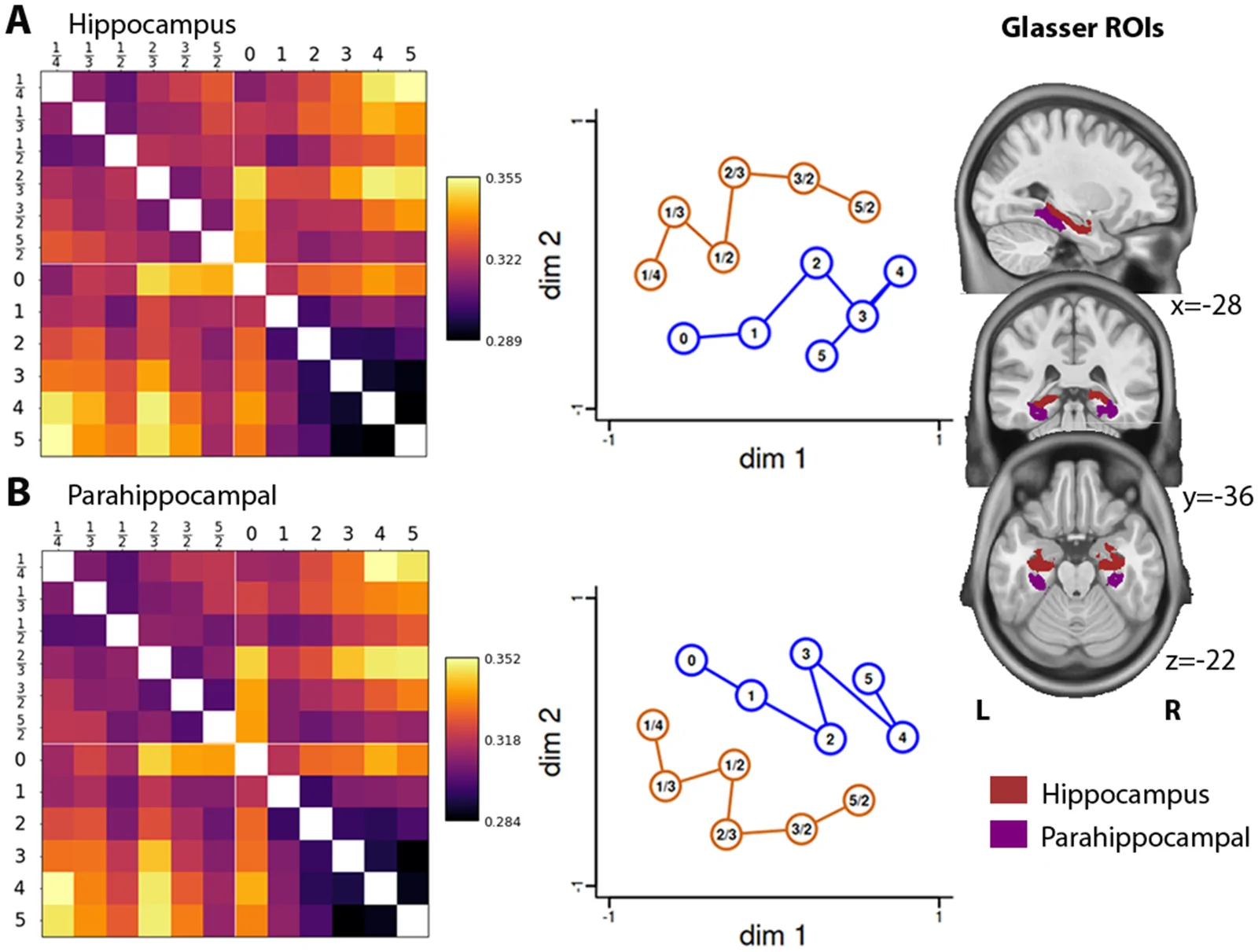
Number line representations within the medial temporal lobe. Same format as Fig 4. **A**) hippocampus, **B**) parahippocampal regions. Both regions were taken from the Glasser atlas and applied to individual participants. The data underlying this Figure can be found in https://doi.org/10.17605/OSF.IO/JQCNA.

**Fig 6:**
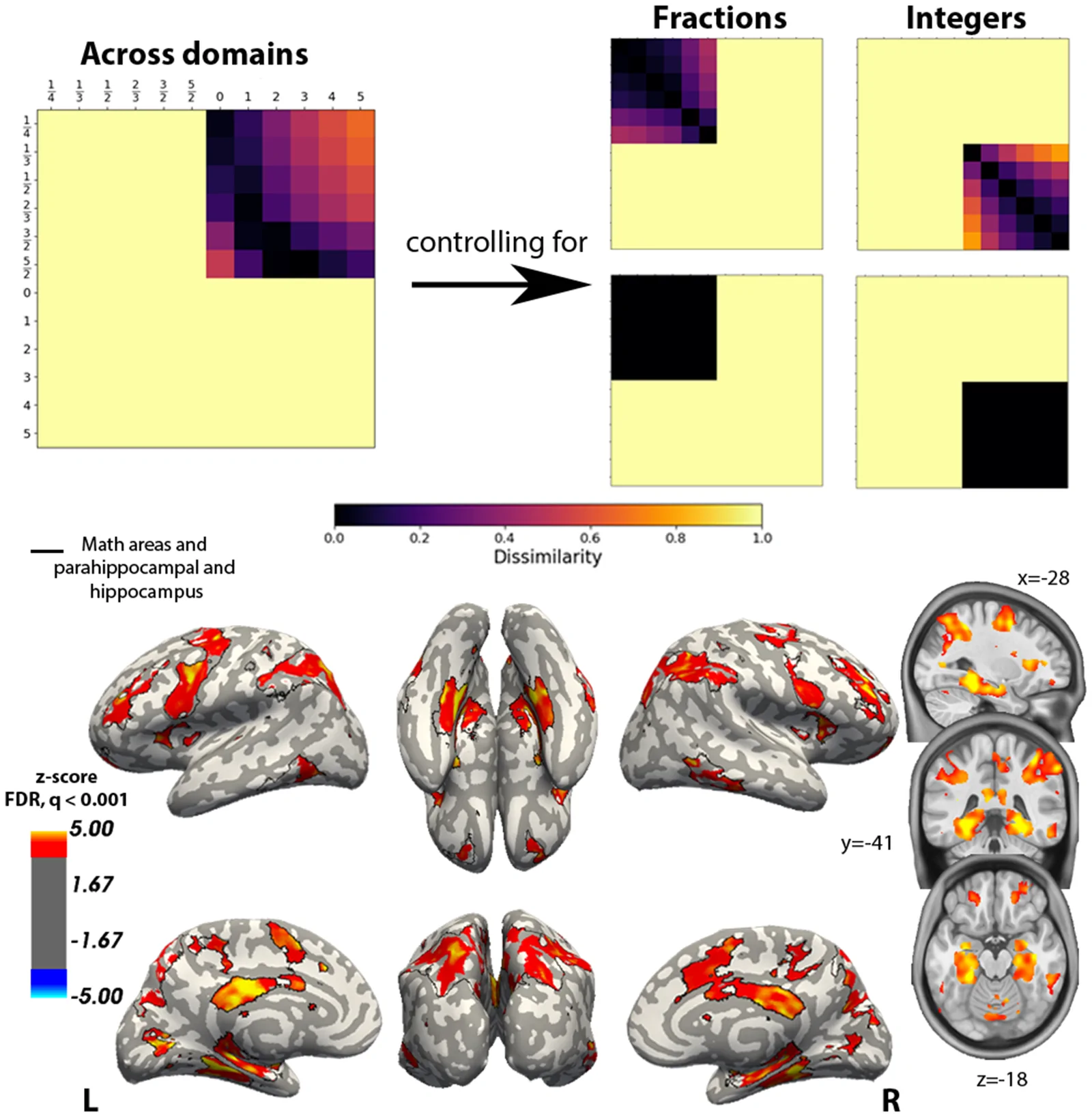
Brain areas sensitive to the numerical distance between fractions and integers. The top panel shows the theoretical RDMs used to model the empirical RDMs. Our focus was on the upper-right quadrant (numerical distance *between* integers and fractions) while controlling for categorical and distance effects *within* each domain. A searchlight analysis restricted to the math-responsive areas and hippocampus and parahippocampal cortex revealed that regions encoding distance between domains (shown in red). These regions included the hippocampus and parahippocampal cortex, as well as cortical regions of the IPS, pITG, MFG, precentral cortex, precuneus, and posterior cingulate. The data underlying this Figure can be found in https://doi.org/10.17605/OSF.IO/JQCNA.

### Individual voxel tuning and numerotopic map

The similarities observed so far between neural activation vectors could, in principle, arise either from distributed representations or from sparse localized neural responses. Furthermore, in the latter case, cortical patches responding to different concepts could either be distributed randomly on the cortical surface or form an overall mesoscopic topographic map [3,5]. Indeed, in the number domain, previous research showed that the firing of individual neurons can be fitted by a Gaussian tuning curve, reflecting their preference for a specific quantity [18,29,50,78]. Furthermore, those preferences cluster on the cortical surface to form “numerotopic” maps that are detectable by fMRI [7,28,47]. Inspired by this prior work, we fitted the present single-voxel fMRI data with Gaussian tuning curves in order to characterize how numerical information is spatially represented and whether a spatial map exists in which voxels corresponding to similar magnitudes are located closer together, regardless of domain (integers or fractions).

We extracted the single-voxel group-average responses to all numbers within the math-responsive areas defined by the localizer. Each voxel’s response profile was fitted by a Gaussian, separately for integers and fractions and for each run, keeping only the voxels that showed greater than 50% variance explained. The presence of reliable voxel tunings was indicated by the fact that, across voxels, the numerical preferences computed in run 1 and run 2 were significantly and positively correlated (r = 0.28, *p* corr = 0.0001 for integers, and r = 0.53, *p* corr = 0.0001 for fractions). There was also a smaller but still reliable correlation between preferred fraction and preferred integer (r = 0.09, *p corr* = 0.004). For brain visualization, in Fig 7A and 7B, as well as S7 Fig, we used the two runs. The graphs of Fig 7A and 7B show the average cross-validated voxel response profiles, after pooling across all the aforementioned voxels (voxel preferences were determined by fitting the data from one run, and then the normalized activation was plotted from the other run). A permutation statistical test confirmed that the preferred number identified in one run indeed led to significantly stronger activation to that target number than to others in the other run (for integers *p corr* = 0.013, and for fractions *p corr* = 0.017).

**Fig 7:**
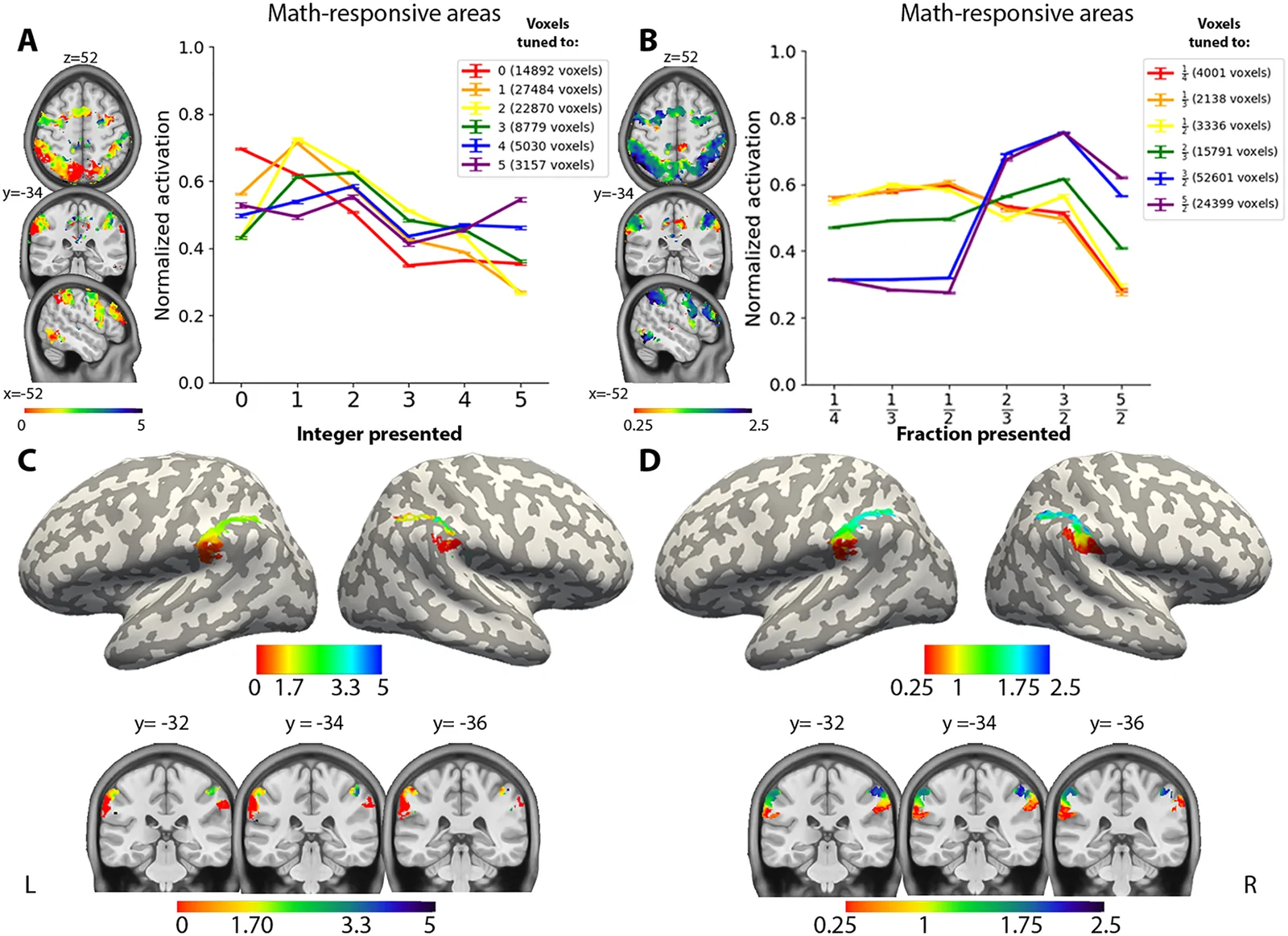
Evidence of voxel tuning to specific numbers and of an overall numerotopic map. **A, B)** Cross-validated plots of normalized activations within voxels identified by Gaussian fitting as tuned to a specific integer (A) or a specific fraction (B). All voxels within math-responsive areas were included in this plot. **C, D)** Surface view (top) and coronal slices (bottom) showing voxel-preferences for integers (C) and fractions (D) across several voxels within anterior IPS and IPL. This bilateral region comprises a congruent numerotopic map across integers and fractions, in which larger numbers are represented with bluish hues and smaller numbers with reddish hues. The map is shown using data averaged across participants; see S9 Fig for examples of single-subject maps. The data underlying this Figure can be found in https://doi.org/10.17605/OSF.IO/JQCNA.

For integers, a clear distance effect can be seen in Fig 7A, with activation decreasing as the numerical distance to the preferred number increased. This effect was prominent for voxels tuned to numbers 0, 1, 2, and to a lesser extent 3, but became undetectable for numbers 4 and 5, probably because very few voxels were tuned to the latter numbers. For fractions in Fig 7B, the distance effect was less evident, perhaps because magnitudes were closer, but a reliable distinction was observed between voxels tuned to fractions, those tuned to fraction $\frac{\text{2}}{\text{3}}$, and those tuned to fractions larger than 1. No such reliable tuning was observed for integers in the hippocampus (*p corr* = 0.62) and parahippocampal cortex (*p corr* = 0.11), though a statistical effect was found for fractions in the hippocampus (r = 0.49, *p corr* = 0.0001) but not for the parahippocampal cortex (*p corr* = 0.51).

When analyzing separately each of the three main math-responsive regions through a Monte Carlo simulation approach, only the IPS showed a positive and statistically significant correlation between fraction and integer magnitudes (integers: r = 0.23, *p corr* = 0.0001; fractions: r = 0.80, *p corr* = 0.0001; across the two domains r = 0.28, *p corr* = 0.0003). Although within-domain correlations were significant in MFG and pITG (MFG: integers: r = 0.53, *p corr* = 0.0001; fractions: r = 0.49, *p corr* = 0.0001; pITG: integers: r = 0.31, *p corr* = 0.0001; fractions: r = 0.58, *p corr* = 0.0001), cross-domain correlations were not (MFG: r = −0.26; pITG: r = −0.11).

Within the parietal lobe, a continuous cortical map from larger to smaller numbers was observed, overlapping with areas PF and IP2 of the HCP atlas. As shown in Fig 7C and 7D, the spatial patterns were broadly similar for fractions and integers, with smaller numbers represented in the most anterior part of the IPS, extending into the inferior parietal lobule, and increasingly larger numbers represented increasingly posteriorly in the horizontal part of the intraparietal sulcus. In these areas, consistent tuning was observed both within domain across the two independent runs (integers: r = 0.44, *p corr* = 0.0001; fractions: r = 0.91, *p corr* = 0.0001), as well as across the two domains (r = 0.69, *p corr* = 0.0003). Furthermore, a topographic map was confirmed by correlations between voxel tuning preferences and the Z (inferior-superior) and X (left-right) coordinates of the MNI atlas, both for integers (Z: left *r* = 0.21, *p corr* = 0.06; right *r* = 0.41, *p corr* = 0.003; X: left *r* = 0.61, *p corr* = 0.001; right *r* = −0.31, *p corr* = 0.03) and for fractions (Z: left *r* = 0.83, *p corr* = 0.0001; right *r* = 0.70, *p corr* = 0.0001; X: left *r* = 0.56, *p corr* = 0.0001; right *r* = −0.58, *p corr* = 0.0001). In contrast, correlations with Y coordinate (anterior-posterior direction) were weaker (Y: left *r* = −0.34, *p corr* = 0.004; right *r* = 0.08, *p corr* = 0.7) and fractions (Y: left *r* = −0.36, *p corr* = 0.002; right *r* = −0.30, *p corr* = 0.01).

Finally, as a further proof of cross-domain consistency, when selecting voxels preferring numbers 0, 1, 2, or 3 in the integer map, we found that these voxels exhibited magnitude preferences consistent with fractional quantities. For example, voxels most responsive to 0 in the integer map also showed a higher activation for the fractions $\frac{\text{1}}{\text{4}}$, $\frac{\text{1}}{\text{3}}$, and $\frac{\text{1}}{\text{2}}$ (S7C Fig). Importantly, this topographic organization remained evident in the aIPS/IPL even when the analysis was restricted to within-domain number comparison trials (i.e., integers preceded by integers, and fractions preceded by fractions; see S8 Fig).

The above results were obtained at the group level. At the single-subject level, although there was considerable variability across participants, the same general topographic pattern in the aIPS/IPL was observed in 14 participants for integers and 15 participants for fractions (at R^2^ > 0.3). Maps for fractions and integers from four representative participants are shown in S9 and S10 Figs shows the topographic progression for each hemisphere and map.

## Discussion

The ability to understand nonsymbolic number magnitudes and ratios is present in babies [11,12], is shared with other species [79,80], and has been consistently mapped in the brain [7,81,82]. Yet, the symbolic systems that extend these foundational abilities, allowing us to represent novel concepts such as fractions and the symbol zero, reflect a uniquely human cognitive achievement. Despite their central role in mathematical reasoning, the neural mechanisms underlying symbolic numbers and their extension to such higher-level mathematical concepts remain much less understood. To investigate this, we used high-field fMRI during a number comparison task mixing integers and fractions to explore how symbolic numbers are represented at both behavioral and neural levels. Behaviorally, we observed the classic distance effect: participants were slower and less accurate when comparing numbers that were closer in magnitude, regardless of their domain (integers or fractions). Neurally, regions within the math network encoded number magnitude in a graded fashion: numbers closer in magnitude evoked more similar fMRI activation patterns, while those farther apart produced more dissimilar patterns. This pattern was also observed in the hippocampus and parahippocampal cortex. The neural representational geometry revealed an organization of neural activity along two distinct dimensions: one representing numerical domain, and another ordering numbers according to their magnitude along a cortical number line. Individual voxels showed reliable preferences for specific integers and fractions, and these preferences formed an organized mesoscopic “numerotopic” map in the bilateral aIPS/IPL, with similar voxel-tuning preferences for fractions and integers, suggesting that during active magnitude processing these numerical domains are integrated into a single semantic map. Thus, the aIPS/IPL appears to play a key role in representing the magnitude associated with a symbolic number, whether it is a fraction or an integer.

Fractions differ from integers in several fundamental ways that could impact how they are mentally represented: 1) unlike integers, they are not organized according to successor relations; 2) an infinite number of fractions exist between any two numbers; and 3) their magnitude is not transparently related to the size of their component numbers (for instance, although 5 is greater than 3, $\frac{\text{1}}{\text{5}}$ is smaller than $\frac{\text{1}}{\text{3}}$). All these properties make fractions counterintuitive and difficult to learn, interfering with the formation of a mental number line for fractions in children [36,37,45]. Nevertheless, our behavioral and brain-imaging findings indicated that, by adulthood, fractions and integers are well integrated into a single semantic space, at least within the small range we tested (0–5). These findings suggest that, to perform the task, participants transformed fractions and integers into a joint representation of their magnitude, thus mapping them onto a shared mental number line. Thus, by adulthood, in our educated participants, both evolutionary older concepts of integers and more recent and culture-dependent concepts of fractions have become integrated into a single representational map.

This conclusion fits with several prior behavioral and brain-imaging studies, which indicate that, by 5th grade, the quantity meaning of fractions begins to be grasped and organized onto a mental number line [34,40,83,84], though this development may be more protracted in French children [45]. Further converging evidence comes from Jacob and Nieder [38], who used an adaptation paradigm to show that participants habituated to a given fraction (e.g., $\frac{\text{1}}{\text{6}}$), when subsequently presented with a fraction in numerical or word format (e.g., $\frac{\text{1}}{\text{2}}$ or “one-half”), showed a recovery of fMRI activity in the bilateral anterior IPS that varied as a function of the numerical distance between the deviant and the adaptor fraction. Our conclusion contrasts, however, with a single fMRI study suggesting that the neural code for fractions differs from that for integers [85]. Closer examination, however, indicates that this is only a superficial discrepancy. Like ours, this study found a greater activation of the entire math-responsive network to fractions than to integers [85], Fig 4), and unlike us, it did not evaluate the fine-grained representational similarities of magnitudes across those domains. Furthermore, while our task required explicit comparisons of the magnitudes of fractions and integers, DeWolf and colleagues did not require direct comparisons between them, but presented integers, decimals and fractions in different fMRI runs, thus allowing participants to rely on domain-specific strategies (e.g., comparing the distance between denominator and numerator) rather than translating fractions into their numerical magnitude [40].

Our findings align with previous studies showing that the bilateral IPS is a key site where numerical magnitude is represented independently of notation [26,37–39,86–90]; but for a distinct viewpoint, see [91–96]. Like others (e.g., [47,97]), we also found that magnitude information is present within the entire math-responsive network, also including the bilateral hippocampus and parahippocampal cortices. This raises an important question: if the aIPS/IPL suffices to translate a number into its associated internal quantity, why do other core areas of the math network, as well as the medial temporal lobe, also appear to represent information according to its magnitude? A possible explanation is that once number symbols are translated into a numerical magnitude, this information may be broadcast to other regions forming a “global workspace” to support decision-making and other working-memory dependent mathematical manipulations [98,99]. Since fMRI is unable to decisively determine the direction of information flow in this network, an important future study would be to investigate the temporal dynamics of symbol comprehension and access to magnitude information using magnetoencephalography (MEG) or intracranial recordings [31,100,101].

Interestingly, an exploratory analysis showed that the hippocampus and parahippocampal cortex may also represent information about integers and fractions in a graded manner, both within and between domains. This finding is in line with recent studies suggesting that magnitude information may be encoded in the medial temporal lobe [29,102], together with many other conceptual dimensions of words beyond the math domain [4]. One hypothesis is that the hippocampus plays a critical but time-limited role in the early phases of knowledge acquisition, by laying out the explicit structure of the learned domain [103,104], before this knowledge gets transferred to the cortex during skill development, as less efficient explicit procedures gradually give way to more automatic and memory-based approaches [105]. An interesting avenue for future studies would be to investigate whether participants with different levels of mathematical education show systematic differences in how the hippocampus is recruited during numerical processing, and whether the organization of numerical maps in the aIPS/IPL varies accordingly.

In all the cognitive maps that we inferred, from behavior (Fig 1), fMRI PCA (Fig 3), fMRI RSA (Figs 4 and 5) or direct observation of cortical tuning to specific numbers (Fig 7), the neural representational geometry we observed aligned with previous research in showing that the concept zero occupies a position at the beginning of the mental number line [17] and that fractions are encoded according to their genuine magnitude (e.g., $\frac{\text{1}}{\text{4}}$ = 0.25), rather than to their numerator or denominator (e.g., $\frac{\text{1}}{\text{4}}$ was far from 4) [40]. However, it is important to note, as a limitation of our study, that fractions did not cover the entire numerical range represented by integers. This was due to our choice to avoid fractions comprising integers that were not presented as targets (e.g., $\frac{\text{9}}{\text{2}}$ was not presented because 9 did not figure among the integer targets). Nevertheless, when the same principal component analysis was repeated using similar numerical ranges for fractions and integers (i.e., excluding the integers 4 and 5), the results remained unchanged (see S3 Fig). Importantly, the nature of our task, interleaving fractions and integers, likely prompted participants to compare the two domains by relying on a single number line [40] rather than applying distinct normalization strategies to each domain [30,76,106]. Note that, in both behavior and math-responsive areas, the representational geometry inferred from MDS of the RSA matrix did not form a perfect straight line, but instead traced a one-dimensional curve embedded in two-dimensional space. One possible explanation for the latter finding is that, due to the limited set of targets that we could study, the fractions ranged only from 0.25 to 2.5, thus covering only the smaller integers 0–3. This may have made all decisions on numbers 3–5 easier, thus causing an apparent bend in the values above three. As for the curved pattern, a similar curved structure has previously been observed during a variety of ordering tasks in the parietal cortex and prefrontal cortex in humans [17,30,97,107,108] as well as in macaques [109] and is therefore not specific to our study. Another possible explanation is that this curvature might reflect tuned neural responses, whereas a more linear representational structure might be indicative of a monotonic code [97,107]. Moreover, curvature may allow the representational space to encode more than one dimension at once, with projection on one axis decoding stimulus order while projection on another orthogonal axis decodes stimulus difficulty and/or uncertainty (in our case, distance from the extremes 0 and 5) [30]. Several authors also warn against the possibility that this curvature or “horseshoe” pattern could be an artifact of MDS, which attempts to preserve the global distances between all pairs of items and, when it cannot respect those constraints in few dimensions, bends the linear continuum [110–113]. This possibility is all the more real that we found a one-dimensional organization when using the simpler method of principal component analysis (Fig 3B). Identifying the exact representational geometry of the cortical number line is an important problem for further research, which may ultimately require neuronal population recordings.

To the best of our knowledge, this is the first study to investigate the geometry of neural representations in a task involving both integers and fractions, as well as to identify a magnitude-based voxel-tuning preference map for both domains in the aIPS/IPL. By fitting a Gaussian function [47], we found that voxels indeed have consistent and cross-domain preferences for specific numbers (Fig 7A), as found at the single-neuron level in monkeys and humans [18,29,50,78]. Furthermore, in one region (the anterior IPS extending towards the inferior parietal lobule), these preferences were organized according to a mesoscopic cortical map where voxels preferring small numbers are in the front and those preferring larger numbers occupy a more posterior and dorsal position (Fig 7C and 7D). This map overlaps with the horizontal segment of the intraparietal sulcus, an area implicated in numerical magnitude manipulation and whose damage leads to impairments in calculation abilities [24]. While such topographic maps have been ubiquitously identified in response to the numerosity of nonsymbolic concrete sets [7,47–49], only a single study, focusing only on integers 1–7, uncovered tuning for symbolic numbers [28] which, surprisingly, was significant only in temporo-occipito cortex (at a site slightly posterior to the present pITG activation). In the present study, several factors may have facilitated the detection of symbolic tuning. One is the comparison task, which encouraged access to a magnitude representation. To perform the task, participants had to focus on the magnitude of the numbers, which is only one of the dimensions of number concepts [114]. It seems likely that, in educated adults, this magnitude representation preexists to the task and is merely amplified or expanded, as previously observed for other concepts [115]. However, other accounts suggest that multiple representations exist by default, whereas a shared representation may be created when explicitly required by the task [94]. We acknowledge that both of these perspectives are tenable and imply distinct interpretations of the role of our magnitude task: one emphasizes the amplification of pre-existing representations, whereas the other suggests that only passive viewing paradigms (or Stroop-like designs) reveal how numerical information is intrinsically represented in the brain. In our view, one reason why some previous studies failed to find robust effects may be their use of passive viewing paradigms or tasks that did not require explicit access to the numerical quantity, likely resulting in weaker magnitude representations, especially when probed with symbolic numerals. Furthermore, those studies, inspired by classic fMRI retinotopy paradigms, used a cyclic paradigm where numbers very progressively increase from 1 to 7 and back [7,28,47]. Thus, consecutive numbers were highly predictable and often repeated, which may have led to fMRI adaptation [82] and participant boredom. In the present study, all transitions between numbers appeared equally often.

It is likely that the comparison task that we used here influenced how information was represented. Our results show that magnitude is represented as a small-to-large axis along the aIPS/IPL axis. However, what is considered a “small” or “large” number may depend on the task context. For example, if we had used a different numerical range (e.g., 5–10), a similar gradient might have emerged, but with the magnitude “5” attached to the opposite, smaller extreme (as previously observed behaviorally for the SNARC effect [20] and neurally for nonnumerical stimuli [30]). A similar task-dependent rescaling might also occur with fractions. Moreover, because fractions and integers were interleaved, participants may have been required to construct a single number line integrating both. It is possible that, if different runs have been used for integers and fractions, both domains would still follow the smaller-to-larger gradient in the aIPS/IPL axis, but the representations across domains would no longer be aligned. Future studies are necessary to test this hypothesis.

In the future, it might be interesting to expand the present work to decimal numbers such as 2.5, which also constitute an important extension of the number concept beyond integers. Furthermore, decimals and fractions possess the interesting ability to refer to the same quantity (e.g., 1.5 versus $\frac{\text{3}}{\text{2}}$). However, using decimals would still face a perceptual confound, because comparing a decimal with an integer would involve comparing a multi-character string with a single digit. Recent work by Summerfield and colleagues [30,76,106] suggests that when two different contexts are perceived, as in the case of decimals and integers, the brain may represent two distinct parallel number lines for each context. Therefore, with decimals and integers too, one may predict a distinction between these two formats in both behavioral and fMRI analyses, potentially leading to a similar distinction between domain and magnitude as observed here.

Another important factor may be task difficulty: comparisons involving only small integers are relatively easy, and the resulting neural activation may be weak. In contrast, by involving a mixture of fractions and integers, and by imposing a continuous load (since each number had to be kept in working memory in order to be compared to the next one), our sequential comparison task forced participants to continuously think of the magnitude of each number while maintaining sustained attention throughout the task. Indeed, Budgen, Woldorff, and Brannon (2019) showed that when participants engaged in a challenging task that required explicit magnitude processing, such as addition with two digits, the bilateral anterior IPS was similarly engaged by symbolic and nonsymbolic formats, an effect that was not observed during a color-matching task [86]. The relationship between numerical formats seems to be less evident when participants perform easy tasks that do not require explicit magnitude computation, such as deciding if two numbers are equal [94,116]. Finally, the use of a 7T fMRI (1.2 mm isotropic, compared to 1.75 mm isotropic for [28]) likely allowed us to detect finer-grained patterns of brain activity.

While integers are natural to humans, fractions and the symbol zero are cultural tools developed out of scientific and mathematical inventiveness. Our findings suggest that, during active magnitude processing, education allows both fraction and integer concepts to be jointly represented in terms of magnitude on the same internal cognitive and neural map. Furthermore, they provide particular clear support for the existence of cognitive maps [1,2], i.e., geometric neural representations in which concepts with similar meaning are represented by similar vectors. High-field fMRI appears capable of monitoring, not only the large-scale cortical gradients that separate different categories of concepts [3–5], but even the mesoscale representations that distinguish individual concepts within a given domain. With longer acquisition times, future work could extend the present findings to a broader set of math concepts [117] and clarify how cortical topography changes with education.

## Supporting information

S1 Fig Results of an alternative method of measuring task difficulty, efficiency score, calculated by combining information about RTs and the percentage of errors (Accuracy/ RTs) for each pair of numbers. **A**) Efficiency score matrix, where smaller values (closer to purple) indicate greater difficulty in comparing numbers, while larger values (yellow) indicate easier comparisons. **B)** When we correlated the x-coordinates of the MDS solution with numerical magnitude using a Spearman correlation (to account for the curved trajectory of the representation), we found a statistically significant correlation. (rho(10) = 0.86, *p* = 0.0004). A Spearman correlation between y-coordinates of the MDS solution and the Efficiency score difficulty measure (defined as the average value of each column) also revealed a similar tendency that did not reach statistical significance (rho(10) = −0.48, *p* = 0.11). The data underlying this Figure can be found in https://doi.org/10.17605/OSF.IO/JQCNA.
(TIF)

S2 Fig Two-dimensional MDS representation obtained without using the extreme numbers (0 and 5). MDS solutions were obtained using either the behavioral balanced integration score, efficiency score matrix, math-responsive areas, as well as IPS, pITG, MFG, parahippocampal, and hippocampus.
(TIF)

S3 Fig Whole-brain principal component analysis without using larger numbers (i.e., 4 and 5) to have similar ranges to integers and fractions. The first principal component distinguishes fractions and integers, whereas the second component distinguishes smaller and larger numbers. To determine the unique contribution of domain (fractions versus integers) and numerical magnitude on the brain, we conducted separate multiple linear regressions for the first and second principal components. The model predicting the first component was highly significant (*F*(2, 7) = 17.15, *p* = 0.002, R^2^ = 0.78), and the domain was a significant predictor (*b* = 138.96, *t*(7) = 5.84, *p* = 0.0006), whereas magnitude was not (*p* = 0.27). The model predicting the second component was also significant (*F*(2, 7) = 31.95, *p* = 0.003, R^2^ = 0.87) but revealed the opposite effect. Magnitude significantly predicted the second component (*b* = −45.22, *t*(7) = −7.59, *p* = 0.0001), whereas domain not (*p* = 0.74). The data underlying this Figure can be found in https://doi.org/10.17605/OSF.IO/JQCNA. Abbreviations: SFG, superior frontal gyrus; MFG, middle frontal gyrus; IFG, inferior frontal gyrus; SMA, supplementary motor area; PCC, posterior cingulate cortex; ITG, inferior temporal gyrus; MTG, middle temporal gyrus.
(TIF)

S4 Fig Glasser ROIs used on S1 Table and Gaussian fit. (TIF)

S1 Table Effect of fraction-to-integer distance on neural dissimilarity matrices within the regions of interest used in the RSA (S4 and S5 Figs). For each region, a multiple regression of RSA matrices was performed as described in Fig 6. The table reports the mean beta coefficient for the crucial fraction-to-integer distance predictor (averaged across subjects, with standard error of the mean in brackets), the peak MNI coordinates of the region, and the corresponding *Z*-score (derived from a group-level one-sample *t* test of the subject betas against zero). *P*-values were corrected for multiple comparisons using Benjamini–Hochberg FDR.
(DOCX)

S5 Fig Visualization of neural representational spaces for numbers within-domain (i.e., trials in which fractions were preceded by fractions and integers were preceded by integers). Representational similarity matrices (RDMs) and their 2-dimensional multidimensional scale (MDS) solutions were used to visualize the 12-dimensional vector space spanned by number activations within individual voxels-of-interest identified by the math>nonmath contrast in the sentence localizer. **A)** On the left, the RDM shows the average dissimilarity across participants, computed using only within-domain trials. In the middle, voxel overlap across individuals is displayed with lighter colors indicating higher overlap (up to a maximum of 15 participants). On the right is the corresponding neural geometry representation derived from MDS. **B)** Results for the intraparietal sulcus (IPS), including average RDM (on the top); the bottom panel shows participant overlap and the resulting neural geometry. **C)** Analysis of the middle frontal gyrus (MFG). **D)** Analyses for the posterior inferior temporal gyrus (pITG). **E, F)** Corresponding analyses for the hippocampal and parahippocampal cortex ROIs, respectively. These regions were defined using Glasser ROIs.
(TIF)

S6 Fig Reliability of representational similarity matrices (RDMs) estimated from independent subset of trials. RDMs visualize the 24-dimensional vector space spanned by number-evoked activity within voxels-of-interest identified by the math>nonmath contrast in the sentence localizer (*p* < 0.001 unc, at the individual level). To assess reliability, the data were split according to the preceding stimulus, yielding independent estimates for fractions preceded by either a fraction (“f_”) or an integer (“i_”; top left, black-highlighted region), and for integers preceded by either a fraction or an integer (bottom right black-highlighted region). Despite this split, the resulting RDMs (blue dashed squares) preserved two key signatures: higher similarity for repeated presentations of the same target than for different targets (diagonal > nondiagonal) and a graded decrease in similarity with increasing numerical distance. These effects were observed for both fractions (diagonal > nondiagonal similarity: *t*(19) = −6.58, *p* < 0.0001; correlation with numerical distance for nondiagonal terms: *t*(19) = 4.06, *p* = 0.0003) and integers (diagonal > nondiagonal similarity: *t*(19) = −11.28, *p* < 0.0001; numerical distance: *t*(19) = 6.93, *p* < 0.0001). Cross-domain similarity patterns (blue dashed squares) were also highly reproducible across independent subset of trials. For integers, the cross-domain similarity matrices were strongly correlated whether the preceding stimulus was an integer or a fraction (Pearson’s *r*(13) = 0.84, *p corr* = 0.0002; Pearson’s *r*(13) = 0.75, *p corr* = 0.002, respectively). For fractions, the cross-domain similarity matrices (top left, blue dashed squares) were highly correlated whether the preceding stimulus was a fraction or an integer (Pearson’s *r*(13) = 0.66, *p corr* = 0.007; Pearson’s *r*(13) = 0.93, *p corr* < 0.0001, respectively).
(TIF)

S7 Fig Further evidence for voxel tuning to specific numbers. A) Whole-brain view of the voxel tuning preferences for integers and fractions, with all math-responsive regions in the sentence localizer. Overall, the correlation between the two maps is very low (*r* = 0.09), but it is high and significant in the aIPS/IPL (*r* = 0.69) (panel **B**). Furthermore, in the aIPS/IPL, voxels preferring 0, 1, 2, and 3, selected based on their preferences in the integer map, show systematic changes in their normalized activation in response to fractions (panel **C**). The data underlying this Figure can be found in https://doi.org/10.17605/OSF.IO/JQCNA.
(TIF)

S8 Fig Surface view and coronal slices showing voxel-preferences for integers preceded by integers (left) and fractions preceded by fractions (right) across several voxels within anterior IPS and IPL (R^2^ > 0.5). This region comprises a congruent numerotopic map across integers and fractions, in which larger numbers are represented with bluish hues and smaller numbers with reddish hues. The map is shown using data averaged across participants. **C)** Correlation between the map of fractions and integers (*r* = 0.29). **D)** In the aIPS/IPL, voxels preferring 0, 1, 2, and 3, selected based on their preferences in the integer map (using only within-domain trials), show systematic changes in their normalized activation in response to fractions (within-domain). The data underlying this Figure can be found in https://doi.org/10.17605/OSF.IO/JQCNA.
(TIF)

S9 Fig Single-participant maps of numerical preferences for integers (top) and fractions (bottom) in four participants. (TIF)

S10 Fig Topographic progression along the aIPS/IPL ROI for the participants 2, 8, 18, and 22 (see S9 Fig). For each participant, we plotted the topographic progression for each hemisphere and each map (integers or fractions). We extracted the preferred numerical magnitude as a function of cortical distance (in mm), binning magnitudes every 5 mm, and the bars show the standard error mean. Cortical distance was computed geodesically starting from the vertex with the lowest preferred magnitude. Then, a spearman rank correlation was performed between distance and preferred magnitude, using permutation testing to assess statistical significance and applying Benjamini–Hochberg FDR correction for multiple comparisons across the 16 tests. After FDR correction, a statistically significant spatial gradient was confirmed in 14 of 16 individual trajectories (with the vast majority surviving at *p corr* < 0.01). LH, left hemisphere; RH, right hemisphere.
(TIF)

## Abbreviations

ANS: Approximate Number System
ANTs: Advanced Normalization Tools
BIS: Balanced Integration Score
CSF: cerebrospinal fluid
DSS: Discrete Semantic System
EPI: echo planar imaging
FD: Framewise Displacement
FDR: False Discovery Rate
fMRI: functional Magnetic Resonance Imaging
FoV: Field of View
GLM: General Linear Models
GM: Gray Matter
HRF: Hemodynamic Response Function
INU: intensity nonuniformity
IPL: inferior parietal lobe
IPS: intraparietal sulci
MDS: multidimensional scaling
MFG: middle frontal gyri
PCA: Principal Component Analysis
pITG: posterior inferior temporal gyri
RDMs: Representational Dissimilarity Matrices
ROIs: Regions of Interest
RSA: Representational similarity analysis
RSVP: Rapid Serial Visual Presentation
SBRefs: single-band reference
SNARC: Spatial-Numerical Association of Response Codes
SOA: Stimulus Onset Asynchrony
WM: white matter.
