# Supplementary material for: A cortical semantic map integrates fractions and integers in the human brain
Source: PLoS Biol. 2026 Oct 22;24(10):e3003820. doi: 10.1371/journal.pbio.3003820 (PMC13690230; doi:10.1371/journal.pbio.3003820)
Supplement: Supplementary file 1 [file pbio.3003820.s005.docx]

| **Region of interest** | **Average beta** | **MNI peak coordinates** | | | **Z score** | ***P corr*** |
| --- | --- | --- | --- | --- | --- | --- |
|  |  | x | y | z |  |  |
| Math areas | M = 0.35; SEM = 0.06 | -16 | -68 | 2 | 4.57 | < 0.0001 |
| IPS | M = 0.32; SEM = 0.06 | 39 | -42 | 55 | 4.27 | < 0.0001 |
| ITG | M = 0.32; SEM = 0.05 | 42 | -65 | -3 | 4.48 | < 0.0001 |
| MFG | M = 0.34; SEM = 0.05 | 20 | 37 | 39 | 4.51 | < 0.0001 |
| Hippocampus | M = 0.38; SEM =0.06 | -24 | -37 | -8 | 4.68 | < 0.0001 |
| Parahippocampal | M = 0.40; SEM = 0.05 | -26 | -36 | -16 | 5.11 | < 0.0001 |

**S1 Table: Effect of fraction-to-integer distance on neural dissimilarity matrices within the regions of interest used in the RSA (S4 and S5 Fig).** For each region, a multiple regression of RSA matrices was performed as described in Fig 6. The table reports the mean beta coefficient for the crucial fraction-to-integer distance predictor (averaged across subjects, with standard error of the mean in brackets), the peak MNI coordinates of the region, and the corresponding Z-score (derived from a group-level one-sample t-test of the subject betas against zero). P-values were corrected for multiple comparisons using Benjamini-Hochberg FDR.
