# Supplementary material for: A cortical semantic map integrates fractions and integers in the human brain
Source: PLoS Biol. 2026 Oct 22;24(10):e3003820. doi: 10.1371/journal.pbio.3003820 (PMC13690230; doi:10.1371/journal.pbio.3003820)
Supplement: Supplementary file 2 [file pbio.3003820.s014.docx]

Corresponding Author

Daniela Valério

Neurospin, CEA, Saclay

INSERM-CEA Saclay, Cognitive Neuroimaging Unit

Paris, France

[daniela.filipa.valerio@gmail.com](mailto:daniela.filipa.valerio@gmail.com)

July 15^th^, 2026

Dear Professor Christian Schnell,

Subject: Resubmission of manuscript – “A cortical semantic space integrating fractions and integers”.

Thank you very much for your decision on our manuscript entitled “A cortical semantic space integrating fractions and integers”. We greatly appreciate the valuable feedback provided by you and the reviewers.

We have fully revised our manuscript to address the concerns raised. Moreover, below, we also present point-by-point responses to the Reviewers’ comments, as well as your comments. The suggestions have greatly improved our manuscript, making this process very instructive and a positive experience.

We sincerely hope that you and the Reviewers find sufficient merit in our revised manuscript to be accepted and published in *PLOS Biology*. We firmly believe that the manuscript has benefited from the comments and is now much clearer, thus making a stronger contribution to the field.

Looking forward to hearing from you,

Daniela Valério

(on behalf of the other authors)

Point-by-point responses to the Reviewers are presented below. We present the Reviewer’s comments in bold, and our responses in normal font. All quotes from the revised manuscript will be presented underlined. We also provide the manuscript with the main changes highlighted to help Reviewers evaluate the revisions.

**REVIEWS
------------------------------------
Reviewer #1:**

**In the present study, Valério and colleagues use high-field (7T) fMRI to investigate how fractions and integers are represented in the brain during a continuous magnitude comparison task. Using a combination of analyses (univariate, PCA, RSA, MDS, and Gaussian voxel-tuning), they report that neural activation patterns within areas localized as responsive to mathematical sentences are organized along two main dimensions, i.e., a categorical distinction between fractions and integers, and a gradient of numerical magnitude integrating fractions and integers along the same dimension. They further identify a "numerotopic" map in the IPS, where voxels show magnitude preferences across both fractions and integers.**
**The manuscript has a number of strengths, including the use of high-resolution imaging and an analytic approach using several methods applied to a task mixing fractions and integers. The results, showing a systematic organization of symbolic numerical quantities in the adult IPS, are genuinely novel and actually conflict with a previous report that failed to find such organization (Cai et al., 2023). However, there are also several significant limitations that temper my enthusiasm, as they raise questions about whether the findings clearly support the strong claims of the paper regarding how fractions and integers (and even more broadly symbols) are represented in the brain.**

**R:** We thank the Reviewer for all the work on the manuscript. We sincerely believe that the manuscript’s message and readability are now a lot clearer. Throughout the revision, we have aimed to better convey the importance of the task and to highlight that these findings were only possible because educated adults were engaged in a magnitude comparison task.

**COMMENT #1 – My main concerns with this paper all relate to the same fundamental issue, which is that the conclusions are, in my opinion, too broad relative to what the data can actually demonstrate. As it stands, the paper is framed as revealing how the brain represents the meaning of abstract numerical symbols, suggesting that integers and fractions are integrated into a shared cortical semantic space. I do not think the current data support this level of generality, for several reasons that I develop below.**

**1. First, the framing of the paper refers to a broad question about how the brain represents the meaning of abstract symbols, drawing parallels with semantic mapping work (e.g., Huth et al., 2016). However, magnitude is only one dimension of the meaning of a fraction. That meaning also encompasses its part-whole relationship, its equivalence class, its relation to its components, and so on. By using a task that exclusively targets magnitude comparison, the study focuses on one dimension and then concludes that this dimension organizes the representation. It is also worth noting that the semantic mapping studies cited as inspiration (e.g., Huth et al.) typically use passive or minimally demanding paradigms precisely to capture stable representational properties rather than task-induced computations. The contrast with the present continuous comparison task could hardly be sharper.**

**Second, the task used (i.e., a continuous magnitude comparison task in which participants must explicitly judge whether the current number is larger or smaller than the previous one) requires participants to explicitly convert every stimulus (fraction or integer) into a magnitude and compare it to the preceding one. In other words, the task demands that participants construct a shared magnitude representation across fractions and integers on every trial. Finding that the brain represents fractions and integers along a common magnitude dimension is therefore, to some extent, a confirmation that participants are doing what the task asks them to do. To me, the most critical question (i.e., whether fractions and integers are integrated into a shared semantic space as a stable representational property, independently of task demands) is not really answered by the current design. In fact, the authors are aware of this, as they emphasize that previous studies, such as DeWolf et al. (2016), did not find evidence for a shared code because fractions and integers were presented separately, allowing participants to rely on domain-specific strategies. This is a reasonable argument, but it actually undermines the generality of the present findings. I would argue that if the shared representation only emerges when cross-domain comparison is explicitly required (as in the present task) and disappears when it is not (as in DeWolf et al., 2016), then the integration may be task-induced rather than a default property of the number system. What the current data may show is that adults CAN represent fractions and integers on a common magnitude scale when explicitly required to do so, in a fairly specific task with fairly specific quantities (see below), which is a weaker claim than what is currently made in the paper.**

**R:** We would like to thank the reviewer for this valuable comment, with which we largely agree. We now make it clear that magnitude is only one dimension of number concepts, and we emphasize the importance of our magnitude comparison task. Still, we believe that in passive viewing paradigms, or in tasks emphasizing other numerical dimensions, magnitude representations may still be present, but not sufficiently robust to be reliably detected using non-invasive imaging. This is why we used an active magnitude-comparison task that directs participants’ attention to the magnitude, strengthening the accessibility and expression of the dimension. Attention amplification of semantic dimensions according to task demands is a well-demonstrated phenomenon (Çukur et al., 2013) and we merely use it in order to reveal whether, where and how fractions and integers are represented and integrated.

These points have been mentioned at several places in the Discussion, for example:

Line 699:

“The nature of our task, interleaving fractions and integers , likely prompted participants to compare the two domains by relying on a single number line (Toomarian & Hubbard, 2018) rather than applying distinct normalization strategies to each domain (Nelli et al., 2023; Sheahan et al., 2021; Summerfield et al., 2020).”

Line 784:

“In contrast, by involving a mixture of fractions and integers, and by imposing a continuous load (since each number had to be kept in working memory in order to be compared to the next one), our sequential comparison task forced participants to continuously think of the magnitude of each number while maintaining sustained attention throughout the task. Indeed, Budgen, Woldorff, and Brannon (2019) showed that when participants engaged in a challenging task that required explicit magnitude processing, such as addition with two digits, the bilateral anterior IPS was similarly engaged by symbolic and non-symbolic formats, an effect that was not observed during a color-matching task (Bugden et al., 2019). The relationship between numerical formats seems to be less evident when participants perform easy tasks that do not require explicit magnitude computation, such as deciding if two numbers are equal (Lyons et al., 2015).”

We also acknowledge that we must be careful not to overstate the generality of our findings beyond the dimension of magnitude. We now state the importance of the task quite explicitly in Introduction or Abstract in order to avoid surprising the reader.

In the abstract:

“To test this hypothesis, we recorded whole-brain 7T fMRI responses to integer and fraction symbols during an active magnitude comparison task designed to engage magnitude processing.”

“Our results suggest that, when educated adults engage in an explicit magnitude comparison task, their brain can construct an ordered representation that integrates fractions and integers, with a topographic organization in the anterior IPS/IPL.”

In the Introduction, on page 4, lines 74-76:

“Yet, how and where such abstract symbolic mathematical concepts are represented in the brain during a magnitude comparison task, and what kind of semantic space they form, remains poorly understood.”

In the Introduction, on page 5, lines 102-104:

“Here, our goal is to understand how symbolic fractions are represented both neurally and behaviorally during a task that elicits magnitude processing, and whether this representation aligns with a mental number line.”

In the Discussion, starting on line 619:

“Individual voxels showed reliable preferences for specific integers and fractions, and these preferences formed an organized mesoscopic “numerotopic” map in the bilateral aIPS/IPL, with similar voxel-tuning preferences for fractions and integers, suggesting that during active magnitude processing these numerical domains are integrated into a single semantic map.”

In the Discussion, we expanded our view about the role of task, starting on line 742:

“To perform the task, participants had to focus on the magnitude of the numbers, which is only one of the dimensions of number concepts (Dehaene et al., 2025). It seems likely that, in educated adults, this magnitude representation preexists to the task and is merely amplified or expanded, as previously observed for other concepts (Çukur et al., 2013). However, other accounts suggest that, depending on the task goal, different types of number representations may be created (Cohen Kadosh & Walsh, 2009). We acknowledge that both of these perspectives are tenable and imply distinct interpretations of the role of our magnitude task: one emphasizes the amplification of pre-existing representations, whereas the other suggests that only passive viewing paradigms (or Stroop-like designs) reveal how numerical information is intrinsically represented in the brain. In our view, one reason why some previous studies failed to find robust effects may be their use of passive viewing paradigms or tasks that did not require explicit access to the numerical quantity, likely resulting in weaker magnitude representations, especially when probed with symbolic numerals.”

In the Discussion, starting on line 760:

“It is likely that the comparison task that we used here influenced how information was represented. Our results show that magnitude is represented as a small-to-large axis along the aIPS/IPL axis. However, what is considered a “small” or “large” number may depend on the task context. For example, if we had used a different numerical range (e.g., 5 to 10), a similar gradient might have emerged, but with the magnitude “5” attached to the opposite, smaller extreme (as previously observed behaviorally for the SNARC effect (Dehaene et al., 1993) and neurally for non-numerical stimuli (Nelli et al., 2023)). A similar task-dependent rescaling might also occur with fractions. Moreover, because fractions and integers were interleaved, participants may have been required to construct a single number line integrating both. It is possible that, if different runs have been used for integers and fractions, both domains would still follow the smaller-to-larger gradient in the aIPS/IPL axis, but the representations across domains would no longer be aligned. Future studies are necessary to test this hypothesis.”

Conclusion, starting on line 798:

“Our findings suggest that, during active magnitude processing, education allows both fraction and integer concepts to be jointly represented in terms of magnitude on the same internal cognitive and neural map.”

If the Reviewer feels it is important, we would be happy to expand the section related to the task in the Discussion.

**2. Importantly, there may be a missed opportunity to address this issue of task-specificity in the current study. The task actually contains both mixed-domain trials (fraction compared to integer and vice versa) and within-domain trials (fraction compared to fraction, integer compared to integer). If I understand correctly, the authors separate these trial types for the behavioral analyses but not for the fMRI analyses, where a single regressor per target number is used in the GLM regardless of the preceding stimulus. It would be informative to separate these trials in the fMRI analyses as well. If the cross-domain magnitude code in the RSA and tuning analyses is just as strong when restricting to within-domain trials, that would strengthen the claim of a stable shared representation. If, on the other hand, the integration is primarily driven by mixed-domain trials, that would confirm the task-induced interpretation.**

**I would encourage the authors to conduct such an analysis, or at minimum to explicitly discuss this limitation and temper their conclusions accordingly (for instance by framing the findings in terms of task-induced magnitude computation rather than stable semantic organization).**

**R:** We would like to thank Reviewer 1 for this insightful suggestion. We agree that separating the mixed-domain and within-domain trials offers a clever way of testing whether the shared magnitude code is a stable representational property or merely a task-induced computation.

Following the Reviewer’s recommendation, we built a new GLM and re-ran our core analyses, restricting the data exclusively to within-domain transitions (i.e., only fraction trials preceded by fractions, and integer trials preceded by integers). Crucially, our results show that even when eliminating all explicit cross-domain comparisons, the core findings remain highly stable. This may suggest that this integration of fractions and integers is more than an artifact driven primarily by mixed-domain task demands.

In the multivariate analyses (RSA and MDS), across the math-responsive areas and specific ROIs, the representational geometry consistently organizes numbers by both domain (integers vs fractions) and magnitude, similar to our main results (see below Fig S5).

We revised the information under Results, on page 22, line 501:

“Importantly, these results remained stable even when the analysis was restricted to within-domain trials (i.e., integers preceded by integers, and fractions preceded by fractions; see Figure S5).”

**Figure S5: Visualization of neural representational spaces for numbers within-domain** (i.e., trials in which fractions were preceded by fractions and integers were preceded by integers)**.** Representational similarity matrices (RDMs) and their 2-dimensional multidimensional scale (MDS) solutions were used to visualize the 12-dimensional vector space spanned by number activations within individual voxels-of-interest identified by the math>non-math contrast in the sentence localizer. **A)** On the left, the RDM shows the average dissimilarity across participants, computed using only within-domain trials. In the middle, voxel overlap across individuals is displayed with lighter colors indicating higher overlap (up to a maximum of 15 participants). On the right is the corresponding neural geometry representation derived from MDS. **B)** Results for the intraparietal sulcus (IPS), including average RDM (on the top); the bottom panel shows participant overlap and the resulting neural geometry. **C)** Analysis of the middle frontal gyrus (MFG). **D)** Analyses for the posterior inferior temporal gyrus (pITG). **E-F)** Corresponding analyses for the hippocampal and parahippocampal cortex ROIs, respectively. These regions were defined using Glasser ROIs.

In the voxel-tuning preferences, the topographical gradients remained stable for both within-domain integers (especially in the left hemisphere) and within-domain fractions, with smaller numbers localized near the IPL and larger numbers near the aIPS. When correlating the tuning preferences of voxels responsive to both domains (R^2^ > 0.5), we had a correlation of *r* = 0.29, *p* = 0.002. Moreover, when selecting voxels preferring numbers 0, 1, 2, or 3 within the integer-only map (within-domain), these same voxels exhibited matching magnitude preferences for fractions (within-domain). For example, voxels most responsive to 0 in the integer map also showed a higher activation for the fractions $\frac{1}{4}$, $\frac{1}{3}$, and $\frac{1}{2}$ (See Fig S8 below).

**Figure S8:** **Surface view and coronal slices showing voxel-preferences for integers preceded by integers (left) and fractions preceded by fractions (right) across several voxels within anterior IPS and IPL (R^2^ > 0.5).** This region comprises a congruent numerotopic map across integers and fractions, in which larger numbers are represented with bluish hues and smaller numbers with reddish hues. The map is shown using data averaged across participants. **C)** Correlation between the map of fractions and integers (r = 0.29). **D)** In the aIPS/IPL, voxels preferring 0, 1, 2, and 3, selected based on their preferences in the integer map (using only within-domain trials), show systematic changes in their normalized activation in response to fractions (within-domain).

This information is revised under Results, on page 26, starting on line 592:

“Importantly, this topographic organization remained evident in the aIPS/IPL even when the analysis was restricted to within-domain number comparison trials (i.e., integers preceded by integers, and fractions preceded by fractions; see Figure S8).”

Note, however, that this analysis includes only 45% of the original data (reducing the number of transitions per target from 11 to 5), resulting in maps that are noisier than the full dataset. However, the survival of the results under such reduced statistical power strongly suggests the robustness of our results and the possibility of a shared representation.

Furthermore, separating the analyses into within-domain trials and between-domain trials also allowed us to test the reliability of our measures (i.e., by focusing on the black-highlighted squares corresponding to fractions preceded by a fraction ("f_") or an integer ("i_"; top left), and to integers preceded by either a fraction or an integer (bottom right), see Figure S6). We found that, even after splitting the data into two independent halves, we could still estimate an RSA matrix (blue dashed squares) in which the two independent measurements of activation for the same targets showed both high diagonal similarity and a semantic distance effect. This pattern was observed for both fractions (diagonal > non-diagonal similarity: *t*(19) = -6.58, *p* < 0.0001; correlation with numerical distance for non-diagonal terms: *t*(19) = 4.06, *p* = 0.0003) and integers (diagonal > non-diagonal similarity: *t*(19) = -11.28, *p* < 0.0001; correlation with numerical distance for non-diagonal terms: *t*(19) = 6.93, *p* < 0.0001).

Also, the general pattern of cross-domain similarity for integers (bottom right, blue dashed squares) was also replicated, whether the trials were preceded by integers (Pearson's *r*(13) = 0.84, *p corr* = 0.0002) or by fractions (Pearson's *r*(13) = 0.75, *p corr* = 0.002). Similarly, the pattern of cross-domain similarity for fractions (top left, blue dashed squares) was reproduced whether the trials were preceded by fractions (Pearson's *r*(13) = 0.66, *p corr* = 0.007) or by integers (Pearson's *r*(13) = 0.93, *p corr* < 0.0001).

We added the following text to the Results section, page 22, starting line 503:

“Moreover, this separation allowed us to assess the reliability of our brain-activity measures: there was a high correlation between the brain activity patterns measured on within-domain trials and on cross-domain trials (see Figure S6).”

The detailed information was also added to Figure S6 in the Supplementary Material:

**Figure S6: Reliability of representational similarity matrices (RDMs) estimated from independent subset of trials.** RDMs visualize the 24-dimensional vector space spanned by number-evoked activity within voxels-of-interest identified by the math>non-math contrast in the sentence localizer (p < 0.001 unc, at the individual level). To assess reliability, the data were split according to the preceding stimulus, yielding independent estimates for fractions preceded by either a fraction ("f_") or an integer ("i_"; top left, black-highlighted region), and for integers preceded by either a fraction or an integer (bottom right black-highlighted region). Despite this split, the resulting RDMs (blue dashed squares) preserved two key signatures: higher similarity for repeated presentations of the same target than for different targets (diagonal > non-diagonal) and a graded decrease in similarity with increasing numerical distance. These effects were observed for both fractions (diagonal > non-diagonal similarity: *t*(19) = -6.58, *p* < 0.0001; correlation with numerical distance for non-diagonal terms: *t*(19) = 4.06, *p* = 0.0003) and integers (diagonal > non-diagonal similarity: *t*(19) = -11.28, *p* < 0.0001; numerical distance: *t*(19) = 6.93, *p* < 0.0001). Cross-domain similarity patterns (blue dashed squares) were also highly reproducible across independent subset of trials. For integers, the cross-domain similarity matrices were strongly correlated whether the preceding stimulus was an integer or a fraction (Pearson's *r*(13) = 0.84, *p corr* = 0.0002; Pearson's *r*(13) = 0.75, *p corr* = 0.002, respectively). For fractions, the cross-domain similarity matrices (top left, blue dashed squares) were highly correlated whether the preceding stimulus was a fraction or an integer (Pearson's *r*(13) = 0.66, *p corr* = 0.007; Pearson's *r*(13) = 0.93, *p corr* < 0.0001, respectively).

**3. Third, a related concern involves the stimulus set, which further limits the generality of the conclusions. The study uses only six fractions, four of which fall below 1 and the largest of which is 2.5. Meanwhile, integers span 0 to 5. This creates a near-complete confound between number type (fraction vs. integer) and magnitude range. That is, fractions are predominantly small numbers, while integers cover the full range. This confound has consequences for virtually every analysis in the paper. For example, the first PCA component, which separates fractions from integers, could partly reflect a magnitude range difference rather than a categorical distinction. Given this, it is possible that the second component (which orders numbers by magnitude) simply captures residual variance after removing a first component that is itself partly about magnitude. I wonder whether this might explain why the spatial map shown on Figure 3A appears to be almost a negative of the map in Figure 3B (I am concerned here that this could be an artifact of the PCA attempting to orthogonalize two correlated dimensions rather than reflecting two genuinely independent representational axes). Similarly, the cross-domain correlation in the voxel-tuning analysis may be inflated by the fact that the fraction range (0.25 to 2.5) overlaps almost entirely with the lower integer range (0 to 3). Voxels tuned to small magnitudes will respond to both 1/4 and 0, not necessarily because of deep representational integration, but because both happen to be small numbers.**

**I was also surprised that the stimulus set does not include any fractions equivalent to integers. In my opinion, such stimuli would have provided the strongest test of integration. If the brain truly represents fractions and integers on a shared magnitude map, then a fraction such as 6/2 and an integer such as 3 should produce nearly identical activation vectors despite their different notation. In the absence of such comparisons, the paper cannot clearly dissociate the contribution of numerical magnitude from the contribution of notation format. It is also striking that these six fractions are quite simple and likely well-memorized by educated adults, making it unclear to what extent the findings would generalize to more complex or less familiar fractions.**

**R:** We thank Reviewer 1 for this detailed and thoughtful comment, which provided a great opportunity to think about our findings. We recognize that one limitation of our study is that we tested only a small set of stimuli, and that the selected fractions did not cover the entire numerical range of the integers we probed. We disagree, however, with the statement that “This creates a near-complete confound between number type (fraction vs. integer) and magnitude range”. There was quite a significant variation in magnitude, both within fractions and within integers, and indeed our results revealed very different principal components sensitive to magnitude and to number type.

The stimulus set resulted from several methodological decisions:

1) Each number (integer or fraction) had to be preceded by every other number exactly once. With 12 stimuli, this resulted in 132 transitions, and each run lasted almost 10 minutes.
2) We selected fractions that could be constructed **from the same integers**, allowing us to test whether participants represented quantities rather than merely perceptual features (e.g., whether $\frac{1}{4}$ would be represented far from 4, or whether 1/3 would be represented far from 3 and closer to 0). Had we selected fractions such as $\frac{9}{2}$, the referee may have rightly complained that number 9 was never presented as an integer! We opted to keep the component integers constant, as the expense of a small downward shift in the range of fraction magnitudes compared to integers.
3) We did not include fractions as $\frac{6}{2}$ together with the integer 3 because the task required participants to compare the current number with the immediately preceding one, so we excluded the equivalent numbers.
4) The task was already cognitively demanding as participants had to sustain attention for nearly 10 min while continuously comparing the current number with the previous one. We therefore tried to select a small set of fractions that were not excessively difficult to not discourage participants, especially because, as shown in Figure 1, participants made a substantial number of errors when fractions were involved.
5) Finally, we intentionally limited the number of fractions and integers and repeated them multiple times in order to (1) obtain stable and reliable measurements at 1.2 mm isotropic, which is not trivial; (2) facilitate the visualization of the graded effects in the RSA, and (3) obtain an interpretable MDS representation.

In the Methods section, we tried to justify our stimuli choices on page 7, starting on line 146:

“Participants were engaged in a serial continuous comparison task, inspired by Nelli et al. (2023), in which each target had to be judged as larger or smaller than the previous one. To achieve that, we avoided consecutive numbers with the same magnitude. The targets consisted of 12 numbers: six integers (i.e., 0, 1, 2, 3, 4, and 5), and six fractions ($\frac{1}{4}$, $\frac{1}{3}$, $\frac{1}{2}$, $\frac{2}{3}$, $\frac{3}{2}$, $\frac{5}{2}$). The stimuli were presented as white Arabic numerals on a black screen for 1 second, with fractions shown using a horizontal line. The small set of familiar fractions was selected to avoid excessive task difficulty and discouragement among participants. Importantly, the fractions were constructed from the same integers, thus allowing us to test whether participants represented magnitude rather than perceptual features (e.g., whether $\frac{1}{4}$ is closer to smaller numbers than to 4). All possible transitions between the 12 target numbers occurred an equal number of times, with no number repeated consecutively, resulting in 11 presentations of each target and 132 unique transitions per run. This relatively small and fully balanced stimulus set facilitates interpretable results in MDS and RSA, while ensuring that each number was preceded exactly once by every other number.”

In the PCA, we found 2 main components. The first distinguishes between fractions and integers, and we can see that it is very similar to the contrast between fractions > integers in Fig 2. Note that this network, involving IPS, ITG, and frontal areas seem to be also very similar to the math-responsive areas identified by the contrast math > non-math sentences. Our interpretation is that these regions seem to be more activated when participants perform challenging tasks, such as comparing fractions (Amalric & Dehaene, 2016; Duncan, 2010). As illustrated in the image below, there is a substantial overlap among activations associated with mathematical sentences, fraction processing, and the left first principal component. In the second component, which is by definition orthogonal, we see that numbers are extremely well organized according to their magnitude, independently of whether they are fractions or integers.

**Figure A: Similarity between the contrast math > non-math sentences, the contrast fractions > integers, and the first principal component.** The figure below shows the overlap between regions preferentially engaged by mathematics and fractions, including the IPS, frontal regions, and ITG.

To formally quantify the contributions of numerical domain and magnitude to each principal component, we performed separate multiple linear regression analyses with domain and magnitude as predictors. These results confirm the qualitative observations from PCA and have been added to the Results section (page 21, starting at line 474):

“To determine the unique contribution of domain (fractions vs integers) and numerical magnitude on the brain, we conducted separate multiple linear regressions for the first and second principal components. For the first principal component, the model fit was highly significant (*F*(2, 9) = 25.47, *p* = 0.0002, R^2^ = 0.82), and domain was a significant predictor (*b* = 132.42, *t*(9) = 6, *p* = 0.0002), whereas magnitude was not (*p* = 0.74). For the second principal component, the model fit was also significant (*F*(2, 9) = 11.98, *p* = 0.003, R^2^ = 0.67) but revealed the opposite effect: magnitude significantly predicted the second component (*b* = -28.17, *t*(9) = -4.82, *p* = 0.0009), whereas domain did not (*p* = 0.13).”Thus, this analysis confirms that number magnitude is a major determinant of number representations which affects a broad set of brain areas. Importantly, these findings remained unchanged when the analysis was repeated after excluding the larger integers 4 and 5, so that integers and fractions covered a similar numerical range (see Figure S3).”

Importantly, in aIPS/IPL, we observe the same pattern that we saw in the voxel-tuning preferences – a code for smaller numbers near the IPL and for larger numbers in the aIPS. We also find regions that were not apparent in PC1, such as the hippocampus and ventrotemporal cortex. If the reviewer’s hypothesis was true – that the fractions > integers contrast was largely driven by the smaller numerical magnitude of most fractions – then we would expect similar activation for both the fractions > integers and smaller > larger contrasts. However, this was not what we observed.

Moreover, if the second component was merely an artifact, we would not expect it to emerge across participants. In fact, the second component was observed in 13 out of 20 participants when individual PCA was performed. Additionally, when we perform RSA, a different type of analysis, in the math-responsive areas, we also observe a similar pattern: a distinction between domains (fractions and integers), together with an organization according to magnitude.

To address the referee’s remark directly, we reanalyzed our data while removing the larger integers 4 and 5, thus having very similar ranges for integers and fractions. The principal component analysis was essentially unchanged (see below Figure S3).

**Figure S3: Whole-brain principal component analysis without using larger numbers (i.e., 4 and 5) to have similar ranges to integers and fractions.** The first principal component distinguishes fractions and integers, whereas the second component distinguishes smaller and larger numbers. To determine the unique contribution of domain (fractions vs integers) and numerical magnitude on the brain, we conducted separate multiple linear regressions for the first and second principal components. The model predicting the first component was highly significant (*F*(2, 7) = 17.15, *p* = 0.002, R^2^ = 0.78), and the domain was a significant predictor (*b* = 138.96, *t*(7) = 5.84, *p* = 0.0006), whereas magnitude was not (*p* = 0.27). The model predicting the second component was also significant (*F*(2, 7) = 31.95, *p* = 0.003, R^2^ = 0.87) but revealed the opposite effect. Magnitude significantly predicted the second component (*b* = -45.22, *t*(7) = -7.59, *p* = 0.0001), whereas domain not (*p* = 0.74). **SFG:** superior frontal gyrus; **MFG:** middle frontal gyrus; **IFG:** inferior frontal gyrus; **SMA:** supplementary motor area; **PCC:** posterior cingulate cortex; **ITG:** inferior temporal gyrus; **MTG:** middle temporal gyrus

Still, we added the limitation of the number small number not covering the entire range of the number line in the Discussion, starting on page 30, line 694:

“However, it is important to note, as a limitation of our study, that fractions did not cover the entire numerical range represented by integers. This was due to our choice to avoid fractions comprising integers that were not presented as targets (e.g., $\frac{9}{2}$ was not presented because 9 did not figure among the integer targets). Nevertheless, when the same principal component analysis was repeated using similar numerical ranges for fractions and integers (i.e., excluding the integers 4 and 5), the results remained unchanged (see Figure S3).”

**4. Fourth, the sample consists entirely of participants who hold a bachelor's degree in science. This is mentioned in the methods but never discussed as a limitation. These individuals are at the high end of math education, which once again limits the ability of the paper to make broad claims about how "education" generally expands the number concept and how cultural inventions like fractions become integrated into the neural number system. Demonstrating this would require comparing individuals with different levels of mathematical training, which is not done here.**

**R:** We would like to thank the Reviewer for bringing this to our attention. We identified a translation error regarding the French educational system. The French high-school diploma “Baccalauréat Scientifique” (Bac S) was incorrectly translated as “Bachelor of Science” (BSc). Actually, the “Bac S” is a high-school degree (typically completed around the age of 18) that focuses on scientific subjects such as mathematics, physics, chemistry, or biology. Because it is not a university-level degree, our participants are not at the high-end of mathematical education, but they were prepared to take a scientific bachelor. Indeed, they still represent a more mathematically trained group than the general population, particularly compared to those who took a high-school degree in arts, literature, economics, or vocational training. Some of these participants later pursued university degrees in science.

We corrected this information in methods, page 7, line 137:

“All participants were right-handed, native French speakers, and held a high-school degree with a scientific background.”

We also agree with the Reviewer that we cannot make broad claims about how education expands number concepts, because our sample is very homogeneous. We tried to suggest this as an interesting future study (which we are actually conducting right now). This information has been added to the Discussion section, page 29, starting at line 684:

“An interesting avenue for future studies would be to investigate whether participants with different levels of mathematical education show systematic differences in how the hippocampus is recruited during numerical processing, and whether the organization of numerical maps in the aIPS/IPL varies accordingly.”

**Overall, the issues highlighted above mean that the conclusions of the paper rest on a very specific set of conditions (i.e., a particular task requiring explicit magnitude comparison, a small set of simple fractions, and a highly selected sample). The discrepancy between these conditions and the broad claims made throughout the title, abstract, and discussion is, in my view, too large. I think the findings are more accurately described as demonstrating that when educated adults are engaged in explicit magnitude comparison, the brain can construct an ordered representation that integrates fractions and integers, with a topographic organization in the anterior IPS/IPL. This is a meaningful contribution, particularly the numerotopic mapping for symbolic numbers, which is genuinely novel. But it is a finding about processing magnitudes under some task demands, not about the stable semantic organization of numerical knowledge, and the paper should be framed accordingly.**

**R:** We agree. The sentence “when educated adults are engaged in explicit magnitude comparison, the brain can construct an ordered representation that integrates fractions and integers, with a topographic organization in the anterior IPS/IPL.” is a very good summary of part of our findings, and we have used it in the abstract. We hope that the referee will agree, however, that the finding of a cortical map, bilaterally, with a precise location, is of course another important contribution.

**COMMENT #2** - **I also have some methodological issues with the paper. One concerns the definition of the mask used to constrain most fMRI analyses. If I understand correctly, the math-responsive mask was defined by taking the union of individual-level activation maps, each thresholded at p < 0.001 uncorrected. This means that a voxel that survives the threshold in only one participant (out of 20) is included in the group mask. Unlike a standard one-sample t-test at the group level, this is not a valid group-level inference and this means that the resulting mask is almost certainly very large and includes voxels that are not reliably math-responsive at the group (let alone at the individual) level. This has downstream consequences for the searchlight RSA (which is conducted within this mask, partly defeating the purpose of a searchlight approach), for the RSA and MDS within "math-responsive areas" (where noisy voxels could dilute or distort the signal), and for the Gaussian voxel-tuning analysis (where fitting tuning curves to non-math voxels may add noise). At minimum, the authors should use a proper group-level contrast (e.g., one-sample t-test, ideally corrected for multiple comparisons) to define the search space. Otherwise, there is not much point localizing brain areas with so little specificity across participants.**

**R:** We would like to thank Reviewer 1 for this helpful feedback. We agree with the referee, and have changed our manuscript accordingly. Our sole goal with our original analyses was to analyze the very same voxels used in single-subject analyses. However, the Reviewer’s concern is spot on. Following the Reviewer’s suggestion, we discarded the uncorrected individual-union mask and replaced it with the group-level contrast of math > non-math sentences (FDR, q < 0.05, as in Fig 2A). We re-ran all the relevant analyses – i.e., RSA searchlight and voxel tuning preferences – and we found that our results did not change significantly. As we did in the RSA, we also broke down the math-responsive areas into IPS, ITG, and MFG using the Glasser ROIs (see the images below). We have updated the text in the Results and Methods as well as Figures 6, 7, and Table S1 accordingly. The ROIs' image was included in the Supplementary Materials.

Under methods:

Starting at the line 328:

“This analysis was performed within a mask of mathematically responsive areas (math > non math sentences, FDR q < 0.05) as defined by the localizer, to which we added Glasser-defined ROIs of hippocampi and parahippocampal cortex.”

Starting at the line 342:

“We then created a matrix in which each column represented a target t-value and each row corresponded to a voxel within the mask of math-related regions defined by the localizer math > non-math sentences, FDR q < 0.05, as well as the Glasser ROIs of IPS, ITG, MFG (see the Fig. S4), parahippocampal, and hippocampus.”

Figure S4: Glasser ROIs used on Table S1 and Gaussian fit.

**COMMENT #3** - **Given the absence of preregistration and the large number of statistical tests conducted across multiple analyses and brain regions, I am concerned about the cumulative risk of false positives. I am not asking for correction across all analyses in the paper. However, I do think that tests within a given analysis should be appropriately corrected. This is particularly relevant for the voxel-tuning correlation analyses, where correlations between preferred numbers are reported separately for all math-responsive areas, both within-domain (across runs) and cross-domain (fractions vs. integers). This amounts to a substantial number of tests, each assessed by a separate permutation test with (I believe) no correction for the number of regions tested. The same concern applies to the RSA results reported in Table S1, where the effect of fraction-to-integer distance is tested separately in six ROIs (I don't think there is a correction for multiple comparisons here).**

**R:** We thank Reviewer 1 for raising this critical methodological point. We completely agree that it is important to control the cumulative risk of false positives across multiple ROIs for each analysis. Following the Reviewer’s recommendation, we applied the Benjamini-Hochberg FDR procedure to correct for multiple comparisons.

For the RSA searchlight analysis on Table S1 (see below), we corrected the corresponding Z-score for multiple comparisons across the 6 ROIs using the test Benjamin-Hochberg FDR. It is important to note that the information did not change significantly.

**Table S1: Effect of fraction-to-integer distance on neural dissimilarity matrices within the regions of interest used in the RSA (Figures S4 and 5).** For each region, a multiple regression of RSA matrices was performed as described in Figure 6. The table reports the mean beta coefficient for the crucial fraction-to-integer distance predictor (averaged across subjects, with standard error of the mean in brackets), the peak MNI coordinates of the region, and the corresponding Z-score (derived from a group-level one-sample t-test of the subject betas against zero). *P-values* were corrected for multiple comparisons using Benjamini-Hochberg FDR.

| **Region of interest** | **Average beta** | **MNI peak coordinates** | | | **Z score** | ***P corr*** |
| --- | --- | --- | --- | --- | --- | --- |
|  |  | x | y | z |  |  |
| Math areas | M = 0.35; SEM = 0.06 | -16 | -68 | 2 | 4.57 | < 0.0001 |
| IPS | M = 0.32; SEM = 0.06 | 39 | -42 | 55 | 4.27 | < 0.0001 |
| ITG | M = 0.32; SEM = 0.05 | 42 | -65 | -3 | 4.48 | < 0.0001 |
| MFG | M = 0.34; SEM = 0.05 | 20 | 37 | 39 | 4.51 | < 0.0001 |
| Hippocampus | M = 0.38; SEM =0.06 | -24 | -37 | -8 | 4.68 | < 0.0001 |
| Parahippocampal | M = 0.40; SEM = 0.05 | -26 | -36 | -16 | 5.11 | < 0.0001 |

We similarly applied the Benjamini-Hochberg FDR correction across all ROIs for each test used: within domain (across runs), and between domains. We have updated the text in the Results section to report these corrected values as *p corr*. We are pleased to report that our core findings remain statistically significant after controlling for multiple comparisons.

Due to the extensive revisions to the Results section following the correction of p-values, we are not reproducing all the text here or highlighting all of these changes in the Results, page 17, line 382.

“To control for the cumulative risk of false positives across ROIs, each permutation analysis was corrected for multiple comparisons across ROIs using the Benjamini-Hochberg FDR test.”

**COMMENT #4 - Several analytical choices in the voxel-tuning analysis should be better justified. The R² threshold is set at 0.5 at the group level and 0.3 at the individual level, but no rationale is provided for these values. The smoothing kernel differs between analyses (4 mm for the tuning analysis, 6 mm for other analyses). The top 1% of voxels was selected for PCA visualization. As the study was not preregistered, these choices could influence the results. The authors should either justify these choices explicitly or demonstrate that the key findings are stable across a range of reasonable parameter values.**

**R:** We would like to thank the Reviewer for this critical feedback. We have expanded our Methods section to clarify these decisions.

Regarding the smoothing, all of our analyses, except for the voxel-tuning analysis, were performed without smoothing. Our choice of unsmooth analyses was to keep the high resolution (1.2 mm isotropic) and therefore a sensitivity to subtle changes in activation patterns, allowing us to detect fine-grained distinctions between numbers. The 6 mm smoothing was applied only at the second level for visualization purposes. For the voxel-tunning analysis, we could not use unsmoothed data, perhaps due to the limited number of data, a single session with two runs. In this way, we smoothed the data at 4 mm at both the individual level as well as the group-averaged data. However, we also tested smoothing levels at 2.4 mm and 6 mm, and the results remained unchanged (see below). This information was updated in Methods, page 13, line 292:

“The data remained unsmoothed for all analyses, but for visualization purposes, we smoothed the data at the group level with a 6 mm kernel. We chose to use unsmoothed data to preserve sensitivity to subtle patterns and fine-grained distinctions between numbers. An exception was made for the Gaussian fit analysis, where the data were smoothed at the individual level with a 4 mm kernel as well as the results of the group-averaged data. Possibly due to a limited amount of data, smoothing was necessary, but the results remained when using either using either 2.4 mm or 4 mm smoothing.”

**Figure B: Voxel tuning-preferences at two different smoothing levels (2.4 mm and 6 mm).**

Regarding the R^2^, we justify our choice in the Methods section, page 16, starting on line 350:

“At the single-subject level, maps were thresholded at R² > 0.3 because this threshold allowed us to reliably inspect maps across the 20 participants while ensuring consistency. Similar thresholds have been used in pRF analysis at the single-participant level, even after averaging data across multiple sessions (e.g., Harvey et al., 2013; Hendrikx et al., 2022). In contrast, group-averaged data provides a significantly higher signal-to-noise ratio, allowing us to use a stricter threshold of R^2^ > 0.5. The same threshold was applied both to individual runs and to the average of the two runs.”

In a principal component analysis, using a more lenient approach (e.g., plotting 5% or 10% of the most activated voxels) would produce similar results, but distributed across a larger portion of the brain. By thresholding at the top 1%, we isolate only the voxels showing the most robust and reliable response to each component.

We updated the Methods under “Principal Component Analysis (PCA)” on page 14, line 311:

“This threshold allowed us to isolate the voxels with the most robust and reliable response to each component, whereas more lenient thresholds preserved the results but extended the coverage to a larger portion of the brain”.

**COMMENT #5 - The discussion of hippocampal findings is fairly speculative relative to the strength of the evidence. The Gaussian tuning analysis shows no reliable tuning for integers in the hippocampus and only marginal effects for fractions. The cross-domain correlation in the hippocampus is not reported separately, and it is unclear whether the hippocampal RSA effects survive the multiple comparison concerns raised above. Yet the discussion devotes substantial space to interpreting hippocampal involvement, and even suggests that hippocampal engagement reflects fractions being a "challenging domain" (p. 27) for adults. This is an interesting hypothesis, but it is entirely post-hoc and unsupported by any analysis relating individual differences in fraction competence to hippocampal activation. I think the authors should be more cautious in their interpretation of the hippocampal findings, or provide supporting analyses.**

**R:** We appreciate the Reviewer’s suggestion, and we also agree that our results are mainly exploratory, although interesting. We shortened the paragraph related to the hippocampus and parahippocampal cortex, and we toned down the interpretation of our findings. This change was made in the Discussion, page 29, starting at line 675:

“Interestingly, an exploratory analysis showed that the hippocampus and parahippocampal cortex may also represent information about integers and fractions in a graded manner, both within and between domains. This finding is in line with recent studies suggesting that magnitude information may be encoded in the medial temporal lobe (Kutter et al., 2023; Menon, 2016), together with many other conceptual dimensions of words beyond the math domain (Franch et al., 2025). One hypothesis is that the hippocampus plays a critical but time-limited role in the early phases of knowledge acquisition, by laying out the explicit structure of the learned domain (Behrens et al., 2018; Garvert et al., 2017), before this knowledge gets transferred to the cortex during skill development, as less efficient explicit procedures gradually give way to more automatic and memory-based approaches (Baram et al., 2024).”

**COMMENT #6 - Finally, the data availability statement indicates that code and figure outputs are shared on OSF, but this has limited use if the fMRI data themselves are not also openly available in a public repository. I understand that sharing raw MRI data may be a concern, but I do not think anything prevents the authors from sharing processed maps and would certainly encourage them to do so.**

**R:** We would like to thank the Reviewer for this suggestion. We made the processed maps publicly available.

In the section of data and code availability:

“Code and outputs for Figures 1-7, as well as the processed maps, have been deposited at the Open Science Foundation (OSF). They are publicly available as of the date of publication and can be accessed here: <https://osf.io/jqcna/overview?view_only=4df1d11274c04fbd9ff4048e74199e8c>”

**Reviewer #2:**

**This manuscript describes an empirical 7T fMRI study aiming to understand how the meaning of abstract symbols is represented throughout the human brain. To do so, the authors use an established behavioural paradigm (continuous judgment magnitude comparison task) to measure how BOLD responses to integers and fractions differ across brain regions known to be associated with mathematical thought as well as other plausible networks of association. The integration of both integer and fraction responses into a "joint semantic map" is taken to suggest that the process of enculturation/education leads to a converging of cortical representations that would presumably support shared decision-making and more abstracted mathematical thought across formats. While I have a number of concerns with the current version of the manuscript, I hope these can be straightforwardly addressed with minor revisions.**

**R:** We thank the Reviewer for all the work. We hope our answer adequately addresses your comments!

**1. Given the subject matter, I would have expected some engagement with the distinction between quantical (e.g., quantity discrimination) and numerical (exact, symbolic) cognition, following Núñez (2017) etc. Also, some greater consideration of the need to distinguish between numerosity, numerousness, and number language terms following Dos Santos (2022) would seem warranted given the nature of the claims being made about representations of integers and fractions.**

**The framing of conceptual spaces or "semantic maps" following Gardenfors (2000) is brought up initially but is not applied in any meaningful way as far as I have read Gardenfors; e.g., tests of convexity, any consideration of what would be a "prototypical" integer or fraction to be used as stimuli. MDS is potentially related to the concept of conceptual spaces but otherwise there is little engagement with this theoretical work.**

**There is also mention of "gradients of neuronal activation on the surface of the cortex" (line 50) and "map-like organization" (lines 52-53) in the Introduction without reference to most of the work by Ben Harvey and colleagues on non-symbolic topographic maps of numerosity that would seem very pertinent here, even if not strictly using symbols.**

**Refs:**

**Núñez, R. E. (2017). Is there really an evolved capacity for number? Trends in cognitive sciences, 21(6), 409-424.**

**César Frederico Dos Santos (2022) Re-establishing the distinction between numerosity, numerousness, and number in numerical cognition, Philosophical Psychology, 35:8, 1152-1180, DOI: 10.1080/09515089.2022.2029387**

**R:** We would like to thank the reviewer for the suggestions. We revised the Introduction to clarify the distinction between numerosity, numerousness, and numbers, and to emphasize that our focus is on numbers as a cultural tool. If the Reviewer believes additional clarification or expansion is necessary, we would be happy to provide it.

Page 3, starting on line 61:

“The ability to perceive numerousness (i.e., the subjective sensation of discrete quantities), estimate concrete numerosities (i.e., the objective number of items in a set), and discriminate numerical ratios emerges early in life (Izard et al., 2009; McCrink & Wynn, 2007) and is consistently observed across individuals, regardless of formal education (Dos Santos, 2022; McCrink et al., 2013; Núñez, 2017; Pica et al., 2004). Even the magnitude zero, understood as the quantity of a set with no elements, can be grasped by monkeys and humans (Barnett & Fleming, 2024; Kutter et al., 2024; Nieder, 2016). However, through formal education and the acquisition of symbolic systems, humans move far beyond “quantical cognition” to develop “numerical cognition” (Núñez, 2017), expanding the concept of number even beyond integers.”

The reason we cited Gardenfors was primarily to motivate the idea that semantic similarities between abstract concepts can be represented as distances within a geometric space and modeled using Multidimensional Scaling (MDS) to map these representations. We did not intend to draw on other aspects of Gardenfors’ framework, such as the convexity of a concept or the role of prototypes. To make this clear, we revised the manuscript as follows, starting line 45:

“A plausible hypothesis is that concepts are mapped onto internal conceptual spaces, or “semantic maps”, in which semantic similarities are represented as distances that can be modelled using multidimensional scaling (MDS, Gardenfors, 2000).”

We also agree with the Reviewer regarding the relevance of the work of Ben Harvey and colleagues, and we added the corresponding reference on line 57.

**2. Line 57-59: "…the cerebral representation of numerical symbols, including intuitive ones - such as the integers 0-5 - as well as more abstract forms which are acquired through education - fractions such as 1/2, 1/3, or 5/2."**

**What is exactly intuitive about integer symbols, are they any more intuitive than fraction symbols? Visually simpler perhaps but not necessarily more intuitive. In fact, a case could be made that fraction symbols are more intuitive - a consistent operator is shown as the middle symbol and the numerator and dominator indicate a part/whole relationship. By contrast, individual integer symbols (Indo-Arabic) offer no information about their relationship to quantity or any other mathematical identity. Fractions or rational numbers may signifier more abstract concepts but the point being made is about "the cerebral representation of numerical symbols".**

**R:** We would like to thank the Reviewer for drawing our attention to this issue.

We agree that Arabic integer symbols are cultural inventions and are not inherently “intuitive”, in the sense that the visual shape of “3” does not immediately convey the concept of three things to a child. In this way, Arabic symbolic integers are a cultural invention that must be learned through education, exactly as fractions. Our original phrasing conflated the development intuitiveness of **whole-number concepts** with the **visual symbols** used to represent them.

Indeed, the concept of number as a representation of a quantity, for instance, “three things”, is present in animals (Dehaene et al., 1998; Vallentin & Nieder, 2008), in babies (Izard et al., 2009; McCrink & Wynn, 2007), and even in remote cultures with limited exposure to formal mathematics (McCrink et al., 2013; Pica et al., 2004). However, education allows these basic number concepts to develop into more sophisticated mathematical concepts, such as rational numbers, fractions, or negative numbers. Among these, fractions are particularly difficult to learn (Cauté & Dehaene, 2025; Ischebeck et al., 2009; Schneider & Siegler, 2010). This is the sense in which we interpreted Leopold Kronecker’s famous sentence: “*God made the integers, all else is the work of Man*”.

However, we acknowledge Reviewer 2’s point that the term “intuitive” to describe integers was imprecise and confusing in this context. We have rephrased it on page 3, line 59:

“Here, we tackle this issue by studying a specific domain of semantic competence, central to human education: the cerebral representation of numerical symbols, ranging from simpler numerical concepts – such as the integers 0-5 – to more abstract forms– such as fractions $\frac{1}{2}$, $\frac{1}{3}$, or $\frac{5}{2}$.”

**3. Line 83: You state here that "This process is typically attributed to the Approximate Number System (ANS)" but see the work of Attila Krajcsi on the discrete semantic system (DSS). Some attention to this alternative perspective would seem necessary.**

**Refs:
Krajcsi, A. Numerical distance and size effects dissociate in Indo-Arabic number comparison. Psychon Bull Rev 24, 927-934 (2017).** [**https://doi.org/10.3758/s13423-016-1175-6**](https://doi.org/10.3758/s13423-016-1175-6) **Krajcsi, A., Kojouharova, P., Lengyel, G. (2022). Processing Symbolic Numbers: The Example of Distance and Size Effects. In: Gervain, J., Csibra, G., Kovács, K. (eds) A Life in Cognition. Language, Cognition, and Mind, vol 11. Springer, Cham.** [**https://doi.org/10.1007/978-3-030-66175-5_27**](https://doi.org/10.1007/978-3-030-66175-5_27) **Krajcsi, A., Fedele, M., & Reynvoet, B. (2023). The approximate number system cannot be the leading factor in the acquisition of the first symbolic numbers. Cognitive Development, 65, 101285.
Krajcsi, A., Kojouharova, P. Different sources of the numerical comparison size effect. Mem Cogn (2026).** [**https://doi.org/10.3758/s13421-025-01781-2**](https://doi.org/10.3758/s13421-025-01781-2)

**R:** We appreciate this comment. We updated the Introduction, page 5, on line 97 to introduce this alternative hypothesis to the ANS:

“Note, however, that an alternative account to the ANS is the Discrete Semantic System (DSS), which explains the distance and SNARC effects in symbolic numbers in terms of the strength of the semantic-memory nodes and their connections, as numbers (e.g., 1 and 2) or concepts (e.g., left and small) that co-occur more frequently tend to develop stronger associations (Krajcsi, 2017; Krajcsi et al., 2022, 2023; Krajcsi & Kojouharova, 2026).”

**4. Line 337: Is an efficiency score like the rate correct score (Accuracy / RTs) the best way to measure performance here? Would a balanced integration score and/or some other measure work better? Does the choice of measure effect the MDS result and can you rule out this possibility?**

**R:** We sincerely thank Reviewer 2 for this important methodological suggestion. Following your recommendation, we applied the Balanced Integration Score (BIS; Liesefeld & Janczyk, 2019). We agree that the BIS is a mathematically superior metric for this paradigm, as it efficiently neutralizes individual differences in speed-accuracy-trade-offs without artificial biasing either dimension. As the Reviewer hypothesized, the BIS appears to perform better than our method of Efficiency Score. The results are similar, but less noisy: using the BIS, we can more clearly observe an organization based on magnitude for both integers and fractions. Because the BIS provides a clearer resolution of the underlying cognitive geometry, we decided to replace our original efficiency score with the BIS throughout the main text and primary figures of the revised manuscript (See Figure 1, below).

To implement the BIS for an MDS distance matrix (which requires non-negative values), we implemented the method and function provided by Liesefeld & Janczyk, 2019) and applied a simple baseline shift (by subtracting the minimum value of the matrix)– we made sure that the most difficult comparisons started in 0. Because easier magnitude comparisons correspond to numbers that are further apart on the number line, higher BIS values correspond to greater cognitive distances in the MDS space. We calculated these individual BIS distance matrices symmetrized for each participant and fed them into the indscal MDS algorithm. Figure 1 shows the average matrix across individuals before symmetrizing it.

However, we believe that Efficiency Score is also a relevant measure of task difficulty, and that the convergence of results is reassuring, so we mentioned it in the Supplementary Material (Figure S1, see below).

Under methods, page 13, line 278:

“We analyzed behavioral data by integrating response times and error rates into a Balanced Integration Score (BIS; Liesefeld & Janczyk, 2019), separately for each combination of the 12 numbers, and as a function of the numerical distance between the current and previous number. To ensure that all distance values were non-negative, we subtracted, for each participant, the minimum BIS value from all cells in the matrix.”

We also revised the text under Results, page 18, line 410:

“To combine information from RTs and error rates, we calculated the BIS score for each pair of numbers (Liesefeld & Janczyk, 2019; see Fig. 1D; see Fig. S1 for an alternative measure, Efficiency Score). Values closer to 0 indicate more difficult trials.”

**Figure 1.** **Experimental design and behavioral results. A)** An example of the task sequence in the scanner: participants compared the current number to the previous one. Each number was followed by a fixation cross that turned green when the response was registered and red 500 ms before the next trial to prepare participants. After 45 stimuli, participants received feedback on their percentage of correct answers. Right of panel A, the full set of stimuli is shown, including both integers and fractions. **B)** RTs as a function of absolute distance between current and previous numbers (132 combinations). Panels separate trials in which both the previous and the current numbers were integers (int), both were fractions (frac), and trials with mixed number domains. **C)** Percentage of errors as a function of the absolute distance between current and previous numbers (same format). **D**) Balanced Integration Score (BIS), where smaller values (closer to purple) indicate greater difficulty in comparing numbers, while larger values (yellow) indicate easier comparisons. **E)** Two-dimensional MDS representation of the BIS matrix, showing that fractions and integers are organized according to their magnitudes.

**Figure S1: Results of an alternative method of measuring task difficulty, efficiency score, calculated by combining information about RTs and the percentage of errors (Accuracy / RTs) for each pair of numbers.** **A**) Efficiency score matrix, where smaller values (closer to purple) indicate greater difficulty in comparing numbers, while larger values (yellow) indicate easier comparisons. **B)** When we correlated the x-coordinates of the MDS solution with numerical magnitude using a Spearman correlation (to account for the curved trajectory of the representation), we found a statistically significant correlation. (rho(10) = 0.86, *p* = 0.0004). A Spearman correlation between y-coordinates of the MDS solution and the Efficiency score difficulty measure (defined as the average value of each column) also revealed a similar tendency that did not reach statistical significance (rho(10) = -0.48, *p* = 0.11).

**5. Lines 387-391: Related to the last point, you interpret the two-dimensional MDS scaling qualitatively in terms of magnitudes on the first dimension (x axis), and difficulty on the second dimension (y axis). Is there a quantitative metric to support these observations? For instance, is this scaling pattern different from random arrangement and how different would it need to be for you to retain the current interpretation? Looking at Figure 1E it seems numerosity 2 and 3 are aligned in difficulty (axis 1) - what theory or model of numerical cognition would support that idea?**

**R:** We appreciate Reviewer 2’s question. We agree that our original interpretation of MDS dimensions was largely qualitative, and we welcome the opportunity to provide explicit quantitative metrics and geometric clarifications. To quantitatively verify our interpretation, we extracted the x- and y-coordinates from the MDS solutions (using both the newly adopted BIS metric and the original efficiency score) and correlated them with our experimental variables.

**Dimension 1:** For the x-axis, which we interpreted as representing numerical magnitude, we correlated the integer and fraction values from 0 to 5 with the x-coordinates. We used a spearman correlation because of the pronounced curved trajectory. The x-coordinates significantly correlated with the true numerical of the targets for both the BIS (rho(10) = 0.75, *p =* 0.007), and Efficiency score (rho(10) = 0.87, *p =* 0.0004).

**Dimension 2:** For the y-axis, which we hypothesized as reflecting differences in task difficulty, we first calculated the average column value from the behavioral BIS or Efficiency score matrices as an index of difficulty (where higher BIS or Efficiency score indicate easier comparisons). Spearman correlations between y-coordinates and these behavioral averages per number indicate significant relationships for both the BIS (rho = 0.64, *p* = 0.03) and a similar tendency for the Efficiency score that did not reach statistical significance (rho(10) = -0.48, *p* = 0.11). These results provide quantitative support for our interpretation that dimension 2 might capture differences in task difficulty.

This BIS information was added to the results section, page 19:

Line 426:

“When we correlated x-coordinates of the MDS solution with numerical magnitude using a Spearman correlation (to account for curved or otherwise non-linear representations), we found a statistically significant correlation (rho(10) = 0.75, *p* = 0.007).”

Line 431:

“To obtain a measure of difficulty, we calculated the average value of each column in the behavioral BIS matrix, where higher BIS indicate easier comparisons. A spearman correlation between y-coordinates of the MDS solution and this difficulty measure revealed a significant relationship (rho(10) = 0.64, *p* = 0.03).”

The Efficiency Score information was added to the information of Figure S1:

“When we correlated the x-coordinates of the MDS solution with numerical magnitude using a Spearman correlation (to account for the curved trajectory of the representation), we found a statistically significant correlation. (rho(10) = 0.86, *p* = 0.0004). A Spearman correlation between y-coordinates of the MDS solution and the Efficiency score difficulty measure (defined as the average value of each column) also revealed a similar tendency that did not reach statistical significance (rho(10) = -0.48, *p* = 0.11).”

Regarding the question that observed arrangement may be random, we evaluated the goodness of fit of the indscal MDS solutions, which has been proposed as an appropriate measure of the model fit (Carroll & Chang, 1970). For the BIS-based MDS solution, the model explained 89.7% of the variance of the behavioral data (R^2^ = 0.897). Similarly, for the Efficiency score-based MDS, the model explained 90.7% of the variance (R^2^ = 0.907). To assess whether these values could have arisen by chance, we compared them with the goodness of fit obtained from 1000 INDSCAL MDS solutions generated from symmetrically randomly shuffled BIS and ES distance matrices for each participant. The observed R² values for both MDS solutions were higher than all 1000 random permutations. This result indicates that the two-dimensional geometric representations are highly robust and capture a true underlying structure different from chance (*p* < 0.001).

**6. Lines 390-391: On the comment that "[the second dimension] separated the intrinsically easier numbers 0 and 5 (because they lie at the extremes) from other numbers". You cite work from Dehaene & Changeux (1993) as well as more recently Paul, van Ackooij, ten Cate & Harvey (2022) who highlight monotonically increasing (5) and monotonically decreasing (0) responses may explain responses at both ends of the range amongst tuned responses - could the distinction shown in the second dimension (y axis) instead reflect this possibility, how would you rule this out?**

**R:** We want to thank the Reviewer for this insightful comment. We agree that the spatial separation of 0 and 5 could, in principle, reflect underlying physiological differences in neural coding that cannot be entirely ruled out. However, it is unclear why monotonically tuned neurons would necessarily induce curvature in the MDS solution. Thus, we would like to propose an alternative interpretation regarding the monotonic vs tuned hypothesis for these specific stimuli.

First, although studies by Paul and collaborators (2022) and Karami and collaborators (2019, 2025) have reported a dissociation between monotonic and tuned neural responses, these coding schemes are usually associated with different brain regions (e.g., monotonic coding in early visual areas vs. tuned responses in parietal cortex), rather than with different stimuli within the same representational space. Furthermore, as recently suggested by one of the co-authors, the presence of curvature in MDS solution may rather itself be an indication of tuned representations rather than monotonic coding (Karami et al., 2025, 2026). This information was added to the discussion.

Under Discussion, line 712:

“Another possible explanation is that this curvature might reflect tuned neural responses, whereas a more linear representational structure might be indicative of a monotonic code (Karami et al., 2025, 2026).”

Most crucially, when we recalculated the MDS solution after excluding the extremes of 0 and 5 (Figure S2, a portion of the figure is reproduced below), a pronounced curvature remained evident for both integers and fractions. If the Reviewer’s hypothesis were correct, and that fact that 0 and 5 might rely on different neural codes was the reason for them being closer on the y-axis, then removing these extreme values should have resulted in a more linear representational structure. Instead, the curvature persisted, and dimension 1 continued to track numerical magnitude across both domains. Thus, these observations suggest that the curved representational geometry cannot be explained solely by the inclusion of the extreme values 0 and 5.

**7. Lines 486-530: I had trouble following the descriptions in text and the relevant figure panels in this section. Perhaps it was me, but it seems the text incorrectly refers to panels of Figure 7 and Figure S2 throughout.**

R: We would like to thank Reviewer 2 for identifying this issue. Indeed, we inadvertently confused the figures’ numbers and their description. The text has now been corrected accordingly.

**8. Line 496: what "statistical test"? Same for the results of the next paragraph.**

**R:** We would like to thank Reviewer 2 for pointing out this ambiguity. We agree that the specific statistical tests were not explicitly named in the Results section. To ensure clarity for the reader, we have updated the Results section to explicitly name the permutation test in both paragraphs.

Under Results, page 24, starting in the line 555:

“A permutation statistical test confirmed that the preferred number identified in one run indeed led to significantly stronger activation to that target number than to others in the other run (for integers *p corr* = 0.013, and for fractions *p* *corr* = 0.017).”

Under Results, page 25, starting in the line 567:

“When analyzing separately each of the three main math-responsive regions through a Monte Carlo simulation approach, only the IPS showed a positive and statistically significant correlation between fraction and integer magnitudes (integers: r = 0.23, *p corr* = 0.0001; fractions: r = 0.80, *p* *corr* = 0.0001; across the two domains r = 0.28, *p* *corr* = 0.0003).”

**9. Lines 531-535: I understand the four best participants are shown in Figure S3, but could you compute the topographic progression along the cortical surface to quantify this result as typically done; see Harvey & Dumoulin (2017), Figure 2.**

**R:** We would like to thank Reviewer 2 for this suggestion. Following this recommendation, we defined a continuous map trajectory along the cortical surfaces of our aIPS/IPL ROI for the four representative participants shown in Figure S9 (following the addition of new supplementary figures/analyses). For each hemisphere and each map (integers or fractions), we extracted the preferred numerical magnitude as a function of cortical distance (in mm), binning magnitudes every 5 mm. Cortical distance was computed geodesically starting from the vertex with the lowest preferred magnitude. We, then, computed Spearman rank correlations between cortical distance and preferred magnitude, using permutation testing to assess statistical significance and applying Benjamini-Hochberg FDR correction for multiple comparisons across the 16 tests performed. Even after FDR correction, a statistically significant spatial gradient was confirmed in 14 of 16 individual trajectories (with the vast majority surviving at *p* *corr* < 0.01). In the two cases where the overall correlation did not reach statistical significance (Participant 2 left hemisphere integers map, and Participant 22 left hemisphere, fractions), visual inspection of the plots nevertheless revealed a clear, systematic initial progression until 30/40 mm that abruptly drops at larger cortical distances.

Figure S10: **Topographic progression along the aIPS/IPL ROI for the participants 2, 8, 18, and 22 (see Figure S9).** For each participant, we plotted the topographic progression for each hemisphere and each map (integers or fractions). We extracted the preferred numerical magnitude as a function of cortical distance (in mm), binning magnitudes every 5 mm, and the bars show the standard error mean. Cortical distance was computed geodesically starting from the vertex with the lowest preferred magnitude. Then, a spearman rank correlation was performed between distance and preferred magnitude, using permutation testing to assess statistical significance and applying Benjamini-Hochberg FDR correction for multiple comparisons across the 16 tests. After FDR correction, a statistically significant spatial gradient was confirmed in 14 of 16 individual trajectories (with the vast majority surviving at *p* corr < 0.01). LH – left hemisphere, RH – right hemisphere

Page 26, line 580:

“Maps for fractions and integers from four representative participants are shown in supplementary Figure S9, and Figure S10 shows the topographic progression for each hemisphere and map.”

**10. Graphical comment: Figure 3 does not seem to have an associated colourmap?**

**R:** We would like to thank Reviewer 2 for pointing this out. Previously, we had omitted the color map because the figures were thresholded to show only the top 1% of most active voxels. However, we agree that displaying the intensity gradient within these top 1% voxels might be very informative. We updated Figure 3 to include the corresponding colormap.

**Academic Editor:**

**In addition to the points already raised by the reviewers, I would encourage the authors to consider the following:**

**1. Reviewer 1 articulates well the concern that the manuscript focuses primarily on similarities, with insufficient consideration of their interpretation or underlying causes. There is a substantial body of literature addressing precisely these issues. In particular, the target article by Cohen Kadosh and Walsh (2009, Behavioral and Brain Sciences), as well as earlier work such as Bulthé et al (2014, NeuroImage,** [**https://doi.org/10.1016/j.neuroimage.2013.10.049**](https://doi.org/10.1016/j.neuroimage.2013.10.049)**), Cohen Kadosh et al. (2007, Neuron), Liu et al (2011, PLOS One,** [**https://doi.org/10.1371/journal.pone.0019373**](https://doi.org/10.1371/journal.pone.0019373)**) and Sokolowski et al (2017, NeuroImage,** [**https://doi.org/10.1016/j.neuroimage.2016.10.028**](https://doi.org/10.1016/j.neuroimage.2016.10.028)**) highlight important conceptual and methodological caveats directly relevant to the authors’ approach. These works, among others, are not cited. The manuscript would benefit from a more balanced and cautious perspective, including consideration of analyses to examine potential differences in representations. At present, it misses an opportunity to situate the findings within a broader framework in which multiple representations, potentially including both shared and distinct components shaped by task demands, are considered**.

**R:** We are grateful to the Editor for highlighting these studies, which we have now incorporated into the manuscript, and for the suggestions.

Throughout the Abstract and the Introduction, we have clarified that our findings and conclusions are specific to a task where participants compare magnitudes. In the Discussion, we further elaborate at this point and dedicate a substantial section to the importance of the task as well as our interpretation of the results (as also addressed in Reviewer 1’s first comment, see above).

We have also emphasized that the role of IPS in magnitude processing, regardless of numerical format, remains a matter of ongoing debate in the field:

Under Discussion, page 28, line 657:

“Our findings align with previous studies showing that the bilateral IPS is a key site where numerical magnitude is represented independently of notation (Bugden et al., 2019; Castaldi et al., 2020; Damarla & Just, 2013; Eger et al., 2003, 2009; Ischebeck et al., 2009b; Jacob & Nieder, 2009a; Mock et al., 2018; Piazza et al., 2007; Pinel et al., 2001; but for a distinct viewpoint, see Bulthé et al., 2014, 2015; Cohen Kadosh et al., 2007; Cohen Kadosh & Walsh, 2009; Liu et al., 2011; Sokolowski et al., 2017).”

We hope that Editor finds that our manuscript now has a more balanced and cautious perspective in relation to our findings.

**2. The choice to compare natural fractions (up to 2.5) with integers (up to 5), rather than with decimal fractions of equivalent magnitude, requires stronger justification. In a psychology journal, this caveat would have triggered a rejection.**

**R:** We would like to thank the Editor for raising this important point, echoing referee #1. We hope our response to Reviewer 1 is convincing. One of the most conclusive, I think, is how we reanalyzed the PCA, restricting it to the same range of magnitudes and found very similar results.

Participants were in the scanner and had to decide whether the current number was smaller or larger than the previous one by responding with different hands. Under these conditions, it was not possible to compare equivalent magnitudes. Moreover, due to a series of methodological decisions like having all possible transitions in a single run, a limited set of stimuli to have interpretable RSA and MDS analyses, and time constraints, we were not able to explore all the possibilities (as decimals) in this first study.

Besides, our specific goal was to understand the neural representation of fractions - how information about fractions, a concept known to be notoriously difficult for students, is processed behaviorally and neurally, and whether and how it eventually gets integrated with other numbers. While some authors found clear behavioral and neural results (Ischebeck et al., 2009a; Jacob & Nieder, 2009b) following the known integers pattern, many others had difficulties (e.g., DeWolf et al., 2016).

Our goal was stated in the Introduction, page 5, starting on line 102:

“Here, our goal is to understand how symbolic fractions are represented both neurally and behaviorally during a task that elicits magnitude processing, and whether this representation aligns with a mental number line.”

Nevertheless, we understand the Editor’s concern, as fractions might differ more in the structure/format than decimals and integers. However, replacing fractions and integers would not resolve the perceptual confound. If we used the same number line (0 to 5), comparing a decimal (e.g., 0.75) with an integer (e.g., 1) still compares a complex multicharacter string to a single digit. Recent work of Summerfield and colleagues (Nelli et al., 2023; Sheahan et al., 2021; Summerfield et al., 2020) has suggested that when two contexts are perceived (as with decimals and integers), this will potentially create parallel number lines for each context. Therefore, even if we had used decimals and integers, we would likely still observe a distinction between these two formats in our behavioral and fMRI analyses, likely creating similar results: a distinction between domain and magnitude. Finally, we acknowledge that, in the future, it might be a good idea to compare integers and decimals, but that their comprehension is likely to involve very different processes, including the processing of base 10 positional notation (see e.g. (Dotan & Dehaene, 2020) for an analysis of 2-digit number comprehension).

We agree with the Editor that we need to discuss this point, and we now address this in the Discussion, page 33, starting on lines 772:

“In the future, it might be interesting to expand the present work to decimal numbers such as 2.5, which also constitute an important extension of the number concept beyond integers. Furthermore, decimals and fractions possess the interesting ability to refer to the same quantity (e.g., 1.5 versus $\frac{3}{2}$). However, using decimals would still face a perceptual confound, because comparing a decimal with an integer would involve comparing a multi-character string with a single digit. Recent work by Summerfield and colleagues (Nelli et al., 2023; Sheahan et al., 2021; Summerfield et al., 2020) suggests that when two different contexts are perceived, as in the case of decimals and integers, the brain may represent two distinct parallel number lines for each context. Therefore, with decimals and integers too, one may predict a distinction between these two formats in both behavioral and fMRI analyses, potentially leading to a similar distinction between domain and magnitude as observed here.”

**3. Line 385: It is unclear why the degrees of freedom are reported as 7. It would be helpful to clarify whether the analysis was conducted on data averaged across participants, rather than modelling at the individual level. Ideally, the authors should demonstrate at the participant level that the logarithmic function provides the best fit.**

**R:** We would like to thank the Editor for this question. Indeed, the previous multiple regression analysis was done in the averaged data across participants, but we agree that it hides the individual-level differences. To address the Editor’s concern, we have removed multiple regression averaged data from the manuscript and replaced it with a linear mixed-effects model applied directly to the participant-level data. Moreover, to explicitly test whether the logarithmic function provides the best fit at the participant level, we constructed two competing mixed-effects models: one using absolute linear distance and another using logarithmic distance. Model comparison confirmed that the logarithmic distance provided a superior fit to the data (Δ AIC = 6).

Under Results, page 18, line 412:

“A participant level linear mixed-effects model revealed a logarithmic distance effect between the previous and current numbers (β = 0.79, *t*(2447) = 3.77, *p* = 0.0002), the BIS score decreased when the previous item was a fraction compared to an integer (β = -0.38, *t*(2447) = -4, *p* < 0.0001), and also when the current item was a fraction compared to an integer (β = -0.68, *t*(2447) = -7.04, *p* < 0.0001). The only statistically significant interaction was between the previous and current number conditions, indicating further difficulty when both items were fractions (β = 0.27, *t*(2447) = 2.17, *p* = 0.03). Model comparison confirmed that numerical distance was best modelled logarithmically rather than linearly (Δ AIC = 6).”

**4. The rationale for dividing accuracy by response times is not convincing, particularly given that this approach has been widely criticized. A more appropriate and informative approach would be to apply diffusion modelling and compare drift rates, which would allow a more direct assessment of information processing.**

**R:** We appreciate the Editor’s suggestion. In fact, we revised the main behavioral analysis, replacing the Efficiency score with a Balances integration score as suggested by Reviewer 2. We believe that this change has significantly improved the paper and partly addresses the above criticism.

We also appreciate the value of drift diffusion models of decision making, and indeed we have applied them in the past, including to number comparison data (Dehaene, 2007). We tried it here, although it should be noted that the number of observations is scarce, and therefore makes it very difficult to properly estimate the variability and distribution of response times. Because our continuous fMRI paradigm was optimized to include a single transition between numbers per run, rather than hundreds of trials per transition and individual, fitting a standard drift-diffusion model would have produced unstable parameter estimates. To address the Editor’s request, we applied the EZ-diffusion model (Wagenmakers et al., 2007), a well-established framework that derives drift rate and non-decision time directly from mean RT, RT variance, and accuracy rates for each of the 12x12 numerical transitions.

To do so, however, we had to pool all trials as if they came from a single participant, which is a problematic assumption. Perhaps for this reason, the results, which appear in the figure below, are only partly illuminating. On the positive side, the analysis of drift rate was able to isolate an essentially pure effect of numerical distance, both within and across domains. When drift rates were plotted against absolute distance, we observed a monotonic, quasi-linear increase (B). When plotted against signed distance, the drift rates formed a highly symmetrical V-shape profile (C).

What was less clear was the non-decision time. This variable is supposed to isolate baseline perceptual processing (e.g., visual symbol encoding and motor execution) from the central magnitude extraction task. It did reflect the raw format cost of fractions: participants took much more time to process fractions, and to particularly fraction-to-fraction trials, than integers. However, non-decision time continued to be influenced by distance as well, as shown by the non-orthogonality of the two matrices in the figure below:

**Figure C:** **The EZ-diffusion framework successfully decoupled the structural format costs of the stimuli (non-decision time) from the pure semantic magnitude processing (drift rate).** **A)** The drift rate matrix showed a clear distance effect: numbers closer on the number line (i.e., also near the diagonal) showed lower drift rates, suggesting slower and less efficient processing. In contrast, the non-decision time matrix suggested that fraction-to-fraction transitions required more time for stimulus encoding and response execution than integer-to-integer transitions. Plots of drift rate as a function of absolute distance **(B)** and signed distance **(C)** further demonstrate that numerically closer pairs produced lower drift rates. The opposite pattern was observed for the non-decision time: longer non-decision times were found for fraction-to-fraction transitions and for closer transitions between domains, reflecting the greater encoding demands associated with these more difficult stimuli.

If the Editor considers it important, we would be happy to include this figure and its explanation in the Supplementary Material. However, we find that it complicates the matters rather than shed much light on our task. Fortunately, a drift diffusion analysis is not needed for our conclusions.

**5. Line 493: The reported correlation (r = 0.01, p = 0.008) is extremely small. The degrees of freedom should be reported here, as well as consistently across the manuscript alongside other statistical details (e.g., t values).**

**R:** We would like to thank the reviewer for this suggestion. The degrees of freedom have been added wherever statistical information is reported and where it was appropriated to do so. However, because the voxels were smoothed with a 4 mm FWHM Gaussian kernel, the voxels were not fully independent, making the effective degrees of freedom unreliable. In this way, we used the Monte Carlo simulation approach. This information was added to the Methods section, page 16, starting at line 368:

“To assess the significance of these correlations, we generated 10000 random white-noise brain volumes, smoothed them with a 4mm FWHM kernel to match the spatial smoothness of our real data, and extracted the corresponding voxels to each ROI. For each ROI, we computed the Pearson correlation between the fraction map and each smoothed noise map, generating a null distribution of correlation values. Statistical significance was then calculated by comparing the observed Pearson correlation against this spatially preserved null distribution. This allowed us to test whether the observed correlation between fractions and integers was greater than chance while accounting for the spatial smoothness of the data.”
