# Supplementary material for: A cortical semantic map integrates fractions and integers in the human brain
Source: PLoS Biol. 2026 Oct 22;24(10):e3003820. doi: 10.1371/journal.pbio.3003820 (PMC13690230; doi:10.1371/journal.pbio.3003820)
Supplement: Supplementary file 3 [file pbio.3003820.s015.docx]

**Dear Dr Valério,

Thank you for your patience while we considered your revised manuscript "A cortical semantic space integrating fractions and integers" for publication as a Research Article at PLOS Biology. This revised version of your manuscript has been evaluated by the PLOS Biology editors, the Academic Editor and the original reviewers.

Based on the reviews and on our Academic Editor's assessment of your revision, we are likely to accept this manuscript for publication, provided you satisfactorily address the remaining comments from the Academic Editor. Please also make sure to address the following data and other policy-related requests:

* We would like to suggest a different title to improve its accessibility for our broad audience:
Integers and fractions are integrated in a joint semantic map in the human parietal cortex

* Please add the links to the funding agencies in the Financial Disclosure statement in the manuscript details.

* DATA POLICY:

You may be aware of the PLOS Data Policy, which requires that all data be made available without restriction:** [**http://journals.plos.org/plosbiology/s/data-availability**](http://journals.plos.org/plosbiology/s/data-availability)**. For more information, please also see this editorial:** [**http://dx.doi.org/10.1371/journal.pbio.1001797**](http://dx.doi.org/10.1371/journal.pbio.1001797) **Note that we do not require all raw data. Rather, we ask that all individual quantitative observations that underlie the data summarized in the figures and results of your paper be made available in one of the following forms:

1) Supplementary files (e.g., excel). Please ensure that all data files are uploaded as 'Supporting Information' and are invariably referred to (in the manuscript, figure legends, and the Description field when uploading your files) using the following format verbatim: S1 Data, S2 Data, etc. Multiple panels of a single or even several figures can be included as multiple sheets in one excel file that is saved using exactly the following convention: S1_Data.xlsx (using an underscore).

2) Deposition in a publicly available repository. Please also provide the accession code or a reviewer link so that we may view your data before publication.

Regardless of the method selected, please ensure that you provide the individual numerical values that underlie the summary data displayed in the following figure panels as they are essential for readers to assess your analysis and to reproduce it: 1BC, 7AB, S7C and S8D.

NOTE: the numerical data provided should include all replicates AND the way in which the plotted mean and errors were derived (it should not present only the mean/average values).

Please also ensure that figure legends in your manuscript include information on where the underlying data can be found, and ensure your supplemental data file/s has a legend.

Please ensure that your Data Statement in the submission system accurately describes where your data can be found.

* CODE POLICY

Per journal policy, if you have generated any custom code during the course of this investigation, please make it available without restrictions. Please ensure that the code is sufficiently well documented and reusable, and that your Data Statement in the Editorial Manager submission system accurately describes where your code can be found. More information on our Code Policy, what and how to share can be found here:** [**https://journals.plos.org/plosbiology/s/code-availability**](https://journals.plos.org/plosbiology/s/code-availability) **Please note that we cannot accept sole deposition of code in GitHub, as this could be changed after publication. However, you can archive this version of your publicly available GitHub code to Zenodo. Once you do this, it will generate a DOI number, which you will need to provide in the Data Accessibility Statement (you are welcome to also provide the GitHub access information). See the process for doing this here:** [**https://docs.github.com/en/repositories/archiving-a-github-repository/referencing-and-citing-content**](https://docs.github.com/en/repositories/archiving-a-github-repository/referencing-and-citing-content) **As you address these items, please take this last chance to review your reference list to ensure that it is complete and correct. If you have cited papers that have been retracted, please include the rationale for doing so in the manuscript text, or remove these references and replace them with relevant current references. Any changes to the reference list should be mentioned in the cover letter that accompanies your revised manuscript.

In addition to these revisions, you may need to complete some formatting changes, which you will receive in a follow up email. A member of our team will be in touch with a set of requests shortly. If you do not receive a separate email within a few days, please assume that checks have been completed, and no additional changes are required.

We expect to receive your revised manuscript within two weeks.

To submit your revision, please go to** [**https://www.editorialmanager.com/pbiology/**](https://www.editorialmanager.com/pbiology/) **and log in as an Author. Click the link labelled 'Submissions Needing Revision' to find your submission record. Your revised submission must include the following:
-  a cover letter that should detail your responses to any editorial requests, if applicable, and whether changes have been made to the reference list
-  a Response to Reviewers file that provides a detailed response to the reviewers' comments (if applicable, if not applicable please do not delete your existing 'Response to Reviewers' file.)
-  a track-changes file indicating any changes that you have made to the manuscript.

NOTE: If Supporting Information files are included with your article, note that these are not copyedited and will be published as they are submitted. Please ensure that these files are legible and of high quality (at least 300 dpi) in an easily accessible file format. For this reason, please be aware that any references listed in an SI file will not be indexed. For more information, see our Supporting Information guidelines:**
[**https://journals.plos.org/plosbiology/s/supporting-information**](https://journals.plos.org/plosbiology/s/supporting-information) 

***Published Peer Review History*
Please note that you may have the opportunity to make the peer review history publicly available. The record will include editor decision letters (with reviews) and your responses to reviewer comments. If eligible, we will contact you to opt in or out. Please see here for more details:**
[**https://plos.org/published-peer-review-history/**](https://plos.org/published-peer-review-history/) ***Press*
Should you, your institution's press office or the journal office choose to press release your paper, please ensure you have opted out of Early Article Posting on the submission form. We ask that you notify us as soon as possible if you or your institution is planning to press release the article.

*Protocols deposition*
To enhance the reproducibility of your results, we recommend that if applicable you deposit your laboratory protocols in** [**protocols.io**](http://protocols.io/)**, where a protocol can be assigned its own identifier (DOI) such that it can be cited independently in the future. Additionally, PLOS ONE offers an option for publishing peer-reviewed Lab Protocol articles, which describe protocols hosted on** [**protocols.io**](http://protocols.io/)**. Read more information on sharing protocols at** [**https://plos.org/protocols?utm_medium=editorial-email&utm_source=authorletters&utm_campaign=protocols**](https://plos.org/protocols?utm_medium=editorial-email&utm_source=authorletters&utm_campaign=protocols) **Please do not hesitate to contact me should you have any questions.

Sincerely,
Christian

Christian Schnell, PhD
Senior Editor**
[**cschnell@plos.org**](mailto:cschnell@plos.org) **PLOS Biology**

Corresponding Author

Daniela Valério

Neurospin, CEA, Saclay

INSERM-CEA Saclay, Cognitive Neuroimaging Unit

Paris, France

[daniela.filipa.valerio@gmail.com](mailto:daniela.filipa.valerio@gmail.com)

September 13^th^, 2026

Dear Professor Christian Schnell,

Subject: Resubmission of manuscript, now entitled “A cortical semantic **map** integrating fractions and integers”.

Thank you very much for your decision on our manuscript. We greatly appreciate the constructive feedback provided by you and the Reviewers. Your comments and suggestions have greatly improved the clarity and quality of our work, making this process very instructive and a positive experience.

Regarding the manuscript title, we carefully considered your suggestion “**Integers and fractions are integrated in a joint semantic map in the human parietal cortex”**. We would like to adopt it only in part: the word “map” indeed seems to be more accessible than our original word “space”. However, we believe that, with this new word, our original title “**A cortical semantic map integrating fractions and integers**” is more concise, conveys the broader scope of our findings, and better captures the main results of our paper. In particular, our behavioral, PCA, and even RSA analyses consistently demonstrate that fractions and integers are integrated according to both magnitude and domain in a broad set of regions, not just the parietal cortex. We therefore prefer a title that reflects this overarching spatial organization, which, in PCA and RSA, does not seem to be present only in the parietal cortex, rather than one that focuses only on the voxel tuning preference results.

Following your guidance, we have carefully revised the manuscript to address all remaining concerns and to comply fully with the journal’s requirements and data accessibility.

We are pleased to resubmit our revised manuscript to *PLOS Biology* and believe that the revisions have further improved the clarity, making our paper a stronger contribution to the field.

Looking forward to hearing from you,

Daniela Valério

(on behalf of all authors)Point-by-point responses to the Reviewers are presented below. We present the Reviewer’s comments in bold, and our responses in normal font. All quotes from the revised manuscript will be presented underlined. We also provide the manuscript with the main changes highlighted to help Reviewers evaluate the revisions.

**Reviewer remarks:

Reviewer #1:**

**I would like to thank the authors for having made a genuine effort to address the concerns I raised in the initial review. In particular, the reframing of the claims throughout the abstract, introduction, and discussion now accurately reflects what the data can support, i.e., that during an active magnitude comparison task, the brain constructs an integrated, topographically organized representation of fractions and integers in the IPS. The additional analyses, especially the within-domain trials analysis and the PCA with matched magnitude ranges, strengthen the empirical contribution. I am satisfied with the revised paper, which I believe makes a novel contribution to the literature.
R:** We thank the Reviewer 1 for the kind words and for the constructive feedback throughout the review process. We sincerely believe that the message and readability became clearer. **Reviewer #2:**

**I appreciate the authors have engaged thoroughly with my previous comments and have addressed all of my previously stated concerns:

1. Sufficient clarification in text
2. Resolved concept of "intuitive"
3. Alternative explanation now included
4. I'm glad to see use of the BIS now, which is a more robust measure and seems to provide clearer results than ES (albeit they often agree anyway)
5. Quantitative tests now provide stronger grounding for interpretation of dimensions
6. Alternative explanation now provided
7. Figure/text update can now be followed
8. Clarification of statistical tests easier to follow
9. Individual differences and group trends in orderly topographic progression are now present for readers to interpret
10. Figure now more interpretable

I have no further outstanding concerns and can recommend acceptance.
R:** We thank Reviewer 2 for the careful and hard work on this manuscript, which has substantially improved its clarity and overall quality.

**Comments from the Academic Editor:
1. Lines 746–747: “However, other accounts suggest that, depending on the task goal, different types of number representations may be created (Cohen Kadosh & Walsh, 2009).” This does not accurately represent the cited account. Specifically, the paper proposed that multiple representations exist by default, for example for fractions, integers and non-symbolic quantities, whereas a shared representation may be created when explicitly required by the task. This interpretation actually supports Reviewer 1’s suggestion and would appropriately temper the authors’ stronger claim.**

**R:** We would like to thank the Editor for this suggestion. We agree that this clarification makes the hypothesis clearer:

Page 32, lines 746-748:

“However, other accounts suggest that multiple representations exist by default, whereas a shared representation may be created when explicitly required by the task (Cohen Kadosh & Walsh, 2009).”

**2. Line 157: “This relatively small and fully balanced stimulus set facilitates interpretable results in MDS and RSA…” I would avoid describing the stimulus set as “fully balanced”, given the acknowledged concern regarding the unmatched stimuli. The authors have addressed this caveat well through the additional analyses, but calling the set fully balanced would be an overstatement.**

**R:** We understand the Editor’s concern and have removed the words “fully balanced” from the sentence.

Page 8, lines 156-158:

“This relatively small stimulus set facilitates interpretable results in MDS and RSA, while ensuring that each number was preceded exactly once by every other number.”

**3. Linear mixed-effects model: Could the authors please clarify the random-effects structure, including how participant, trial type and number were modelled, and whether trial type and number were treated as fixed or random effects?**

**R:** We would like to thank the Editor for this suggestion. We have revised the manuscript to make it clearer for the reader:

Page 13, lines 282-286, under the Methods section:

“To the BIS values, we fitted a linear mixed-effects model in which trial type (fraction vs. integer domain for both the current and the previous stimuli) and numerical distance (the log of the absolute difference between current and previous numbers) were included as interacting fixed effects. To account for repeated measures and individual differences in baseline performance, participant was included as a random effect.”

**4. The reported correlation is extremely small but nevertheless statistically significant (r=.01). I can understand how this might arise given the number of observations, but it would be worth confirming that the value is correct and not a typographical or analytical error.**

**R:** We would like to thank the Editor for this comment. In fact, this value has changed since the first submission because we used a different ROI to define the math areas (as suggested by Reviewer 1), and a different method to obtain the p value. In response to your first comment on this issue, we replaced our original method with a Monte Carlo simulation approach, in which we generated 10000 random white-noise brain volumes and smoothed them with a 4mm FWHM kernel to match the spatial smoothness of our real data. As a result of these methodological changes, the value changed to r = 0.09 (p_corr_ = 0.004). We agree that the previous p value was probably inflated because we used a statistic across voxels and which therefore had a large number of observations (N=38573 voxels). This was inappropriate because nearby voxels are not independent of each other. The new approach does not suffer from this problem and the correlation remains statistically significant. To the best of our knowledge, there is no typographical or analytical error, and we have carefully rechecked the analysis to confirm this.
