# Supplementary material for: A cortical semantic map integrates fractions and integers in the human brain
Source: PLoS Biol. 2026 Oct 22;24(10):e3003820. doi: 10.1371/journal.pbio.3003820 (PMC13690230; doi:10.1371/journal.pbio.3003820)
Supplement: Peer review history

# Editor report: Decision Letter 0

**DOI:** 10.1371/journal.pbio.3003820.r001

**Authors:** Christian Schnell, PhD

23 Mar 2026

Dear Dr Valério,

Thank you for submitting your manuscript entitled "A cortical semantic space integrating fractions and integers" for consideration as a Research Article by PLOS Biology.

Your manuscript has now been evaluated by the PLOS Biology editorial staff and I am writing to let you know that we would like to send your submission out for external peer review. Please note that we were unable so far to find an academic editor to handle your manuscript, so we have not yet made a firm decision on whether the advance is sufficient for PLOS Biology. We will discuss this aspect with an academic editor once we have received the reviewer reports.

Before we can send your manuscript to reviewers, we need you to complete your submission by providing the metadata that is required for full assessment. To this end, please login to Editorial Manager where you will find the paper in the 'Submissions Needing Revisions' folder on your homepage. Please click 'Revise Submission' from the Action Links and complete all additional questions in the submission questionnaire.

Once your full submission is complete, your paper will undergo a series of checks in preparation for peer review. After your manuscript has passed the checks it will be sent out for review. To provide the metadata for your submission, please Login to Editorial Manager (https://www.editorialmanager.com/pbiology) within two working days, i.e. by Mar 25 2026 11:59PM.

If your manuscript has been previously peer-reviewed at another journal, PLOS Biology is willing to work with those reviews in order to avoid re-starting the process. Submission of the previous reviews is entirely optional and our ability to use them effectively will depend on the willingness of the previous journal to confirm the content of the reports and share the reviewer identities. Please note that we reserve the right to invite additional reviewers if we consider that additional/independent reviewers are needed, although we aim to avoid this as far as possible. In our experience, working with previous reviews does save time.

If you would like us to consider previous reviewer reports, please edit your cover letter to let us know and include the name of the journal where the work was previously considered and the manuscript ID it was given. In addition, please upload a response to the reviews as a 'Prior Peer Review' file type, which should include the reports in full and a point-by-point reply detailing how you have or plan to address the reviewers' concerns.

During the process of completing your manuscript submission, you will be invited to opt-in to posting your pre-review manuscript as a bioRxiv preprint. Visit http://journals.plos.org/plosbiology/s/preprints for full details. If you consent to posting your current manuscript as a preprint, please upload a single Preprint PDF.

Feel free to email us at plosbiology@plos.org if you have any queries relating to your submission.

Kind regards,

Christian

Christian Schnell, PhD

Senior Editor

PLOS Biology

cschnell@plos.org

# Peer review: Decision Letter 1

**DOI:** 10.1371/journal.pbio.3003820.r002

**Authors:** Christian Schnell, PhD

8 May 2026

Dear Dr Valério,

Thank you for your patience while your manuscript "A cortical semantic space integrating fractions and integers" was peer-reviewed at PLOS Biology. It has now been evaluated by the PLOS Biology editors, an Academic Editor with relevant expertise, and by several independent reviewers.

In light of the reviews, which you will find at the end of this email, we would like to invite you to revise the work to thoroughly address the reviewers' reports.

As you will see below, the reviewers think that your study is interesting and the results potentially important. However, they also raised a number of concerns, in particular about the theoretical framing being too broad and that the findings might be limited to this specific task which would limit the generalizability and broader interest in the findings. The academic editor has also made a few additional comments, which I have pasted below the reviewer reports.

Given the extent of revision needed, we cannot make a decision about publication until we have seen the revised manuscript and your response to the reviewers' comments. Your revised manuscript is likely to be sent for further evaluation by all or a subset of the reviewers.

In addition to these revisions, you will need to complete some formatting changes, which you will receive in a follow up email. A member of our team will be in touch with a set of requests shortly.

We expect to receive your revised manuscript within 3 months. Please email us (plosbiology@plos.org) if you have any questions or concerns, or would like to request an extension.

At this stage, your manuscript remains formally under active consideration at our journal; please notify us by email if you do not intend to submit a revision so that we may withdraw it.

**IMPORTANT - SUBMITTING YOUR REVISION**

Your revisions should address the specific points made by each reviewer. Please submit the following files along with your revised manuscript:

1. A 'Response to Reviewers' file - this should detail your responses to the editorial requests, present a point-by-point response to all of the reviewers' comments, and indicate the changes made to the manuscript.

*NOTE: In your point-by-point response to the reviewers, please provide the full context of each review. Do not selectively quote paragraphs or sentences to reply to. The entire set of reviewer comments should be present in full and each specific point should be responded to individually, point by point.

You should also cite any additional relevant literature that has been published since the original submission and mention any additional citations in your response.

2. In addition to a clean copy of the manuscript, please also upload a 'track-changes' version of your manuscript that specifies the edits made. This should be uploaded as a "Revised Article with Changes Highlighted" file type.

*Re-submission Checklist*

When you are ready to resubmit your revised manuscript, please refer to this re-submission checklist: https://plos.io/Biology_Checklist

To submit a revised version of your manuscript, please go to https://www.editorialmanager.com/pbiology/ and log in as an Author. Click the link labelled 'Submissions Needing Revision' where you will find your submission record.

Please make sure to read the following important policies and guidelines while preparing your revision:

*Published Peer Review*

Please note while forming your response, if your article is accepted, you may have the opportunity to make the peer review history publicly available. The record will include editor decision letters (with reviews) and your responses to reviewer comments. If eligible, we will contact you to opt in or out. Please see here for more details:

https://blogs.plos.org/plos/2019/05/plos-journals-now-open-for-published-peer-review/

*PLOS Data Policy*

Please note that as a condition of publication PLOS' data policy (http://journals.plos.org/plosbiology/s/data-availability) requires that you make available all data used to draw the conclusions arrived at in your manuscript. If you have not already done so, you must include any data used in your manuscript either in appropriate repositories, within the body of the manuscript, or as supporting information (N.B. this includes any numerical values that were used to generate graphs, histograms etc.). For an example see here: http://www.plosbiology.org/article/info%3Adoi%2F10.1371%2Fjournal.pbio.1001908#s5

*Blot and Gel Data Policy*

We require the original, uncropped and minimally adjusted images supporting all blot and gel results reported in an article's figures or Supporting Information files. We will require these files before a manuscript can be accepted so please prepare them now, if you have not already uploaded them. Please carefully read our guidelines for how to prepare and upload this data: https://journals.plos.org/plosbiology/s/figures#loc-blot-and-gel-reporting-requirements

*Protocols deposition*

To enhance the reproducibility of your results, we recommend that if applicable you deposit your laboratory protocols in protocols.io, where a protocol can be assigned its own identifier (DOI) such that it can be cited independently in the future. Additionally, PLOS ONE offers an option for publishing peer-reviewed Lab Protocol articles, which describe protocols hosted on protocols.io. Read more information on sharing protocols at https://plos.org/protocols?utm_medium=editorial-email&utm_source=authorletters&utm_campaign=protocols

Thank you again for your submission to our journal. We hope that our editorial process has been constructive thus far, and we welcome your feedback at any time. Please don't hesitate to contact us if you have any questions or comments.

Sincerely,

Christian

Christian Schnell, PhD

Senior Editor

PLOS Biology

cschnell@plos.org

------------------------------------

REVIEWS:

Reviewer #1: In the present study, Valério and colleagues use high-field (7T) fMRI to investigate how fractions and integers are represented in the brain during a continuous magnitude comparison task. Using a combination of analyses (univariate, PCA, RSA, MDS, and Gaussian voxel-tuning), they report that neural activation patterns within areas localized as responsive to mathematical sentences are organized along two main dimensions, i.e., a categorical distinction between fractions and integers, and a gradient of numerical magnitude integrating fractions and integers along the same dimension. They further identify a "numerotopic" map in the IPS, where voxels show magnitude preferences across both fractions and integers.

The manuscript has a number of strengths, including the use of high-resolution imaging and an analytic approach using several methods applied to a task mixing fractions and integers. The results, showing a systematic organisation of symbolic numerical quantities in the adult IPS, are genuinely novel and actually conflict with a previous report that failed to find such organization (Cai et al., 2023). However, there are also several significant limitations that temper my enthusiasm, as they raise questions about whether the findings clearly support the strong claims of the paper regarding how fractions and integers (and even more broadly symbols) are represented in the brain.

COMMENT #1

My main concerns with this paper all relate to the same fundamental issue, which is that the conclusions are, in my opinion, too broad relative to what the data can actually demonstrate. As it stands, the paper is framed as revealing how the brain represents the meaning of abstract numerical symbols, suggesting that integers and fractions are integrated into a shared cortical semantic space. I do not think the current data support this level of generality, for several reasons that I develop below.

First, the framing of the paper refers to a broad question about how the brain represents the meaning of abstract symbols, drawing parallels with semantic mapping work (e.g., Huth et al., 2016). However, magnitude is only one dimension of the meaning of a fraction. That meaning also encompasses its part-whole relationship, its equivalence class, its relation to its components, and so on. By using a task that exclusively targets magnitude comparison, the study focuses on one dimension and then concludes that this dimension organizes the representation. It is also worth noting that the semantic mapping studies cited as inspiration (e.g., Huth et al.) typically use passive or minimally demanding paradigms precisely to capture stable representational properties rather than task-induced computations. The contrast with the present continuous comparison task could hardly be sharper.

Second, the task used (i.e., a continuous magnitude comparison task in which participants must explicitly judge whether the current number is larger or smaller than the previous one) requires participants to explicitly convert every stimulus (fraction or integer) into a magnitude and compare it to the preceding one. In other words, the task demands that participants construct a shared magnitude representation across fractions and integers on every trial. Finding that the brain represents fractions and integers along a common magnitude dimension is therefore, to some extent, a confirmation that participants are doing what the task asks them to do. To me, the most critical question (i.e., whether fractions and integers are integrated into a shared semantic space as a stable representational property, independently of task demands) is not really answered by the current design. In fact, the authors are aware of this, as they emphasize that previous studies, such as DeWolf et al. (2016), did not find evidence for a shared code because fractions and integers were presented separately, allowing participants to rely on domain-specific strategies. This is a reasonable argument, but it actually undermines the generality of the present findings. I would argue that if the shared representation only emerges when cross-domain comparison is explicitly required (as in the present task) and disappears when it is not (as in DeWolf et al., 2016), then the integration may be task-induced rather than a default property of the number system. What the current data may show is that adults CAN represent fractions and integers on a common magnitude scale when explicitly required to do so, in a fairly specific task with fairly specific quantities (see below), which is a weaker claim than what is currently made in the paper.

Importantly, there may be a missed opportunity to address this issue of task-specificity in the current study. The task actually contains both mixed-domain trials (fraction compared to integer and vice versa) and within-domain trials (fraction compared to fraction, integer compared to integer). If I understand correctly, the authors separate these trial types for the behavioral analyses but not for the fMRI analyses, where a single regressor per target number is used in the GLM regardless of the preceding stimulus. It would be informative to separate these trials in the fMRI analyses as well. If the cross-domain magnitude code in the RSA and tuning analyses is just as strong when restricting to within-domain trials, that would strengthen the claim of a stable shared representation. If, on the other hand, the integration is primarily driven by mixed-domain trials, that would confirm the task-induced interpretation. I would encourage the authors to conduct such an analysis, or at minimum to explicitly discuss this limitation and temper their conclusions accordingly (for instance by framing the findings in terms of task-induced magnitude computation rather than stable semantic organization).

Third, a related concern involves the stimulus set, which further limits the generality of the conclusions. The study uses only six fractions, four of which fall below 1 and the largest of which is 2.5. Meanwhile, integers span 0 to 5. This creates a near-complete confound between number type (fraction vs. integer) and magnitude range. That is, fractions are predominantly small numbers, while integers cover the full range. This confound has consequences for virtually every analysis in the paper. For example, the first PCA component, which separates fractions from integers, could partly reflect a magnitude range difference rather than a categorical distinction. Given this, it is possible that the second component (which orders numbers by magnitude) simply captures residual variance after removing a first component that is itself partly about magnitude. I wonder whether this might explain why the spatial map shown on Figure 3A appears to be almost a negative of the map in Figure 3B (I am concerned here that this could be an artifact of the PCA attempting to orthogonalize two correlated dimensions rather than reflecting two genuinely independent representational axes). Similarly, the cross-domain correlation in the voxel-tuning analysis may be inflated by the fact that the fraction range (0.25 to 2.5) overlaps almost entirely with the lower integer range (0 to 3). Voxels tuned to small magnitudes will respond to both 1/4 and 0, not necessarily because of deep representational integration, but because both happen to be small numbers.

I was also surprised that the stimulus set does not include any fractions equivalent to integers. In my opinion, such stimuli would have provided the strongest test of integration. If the brain truly represents fractions and integers on a shared magnitude map, then a fraction such as 6/2 and an integer such as 3 should produce nearly identical activation vectors despite their different notation. In the absence of such comparisons, the paper cannot clearly dissociate the contribution of numerical magnitude from the contribution of notation format. It is also striking that these six fractions are quite simple and likely well-memorized by educated adults, making it unclear to what extent the findings would generalize to more complex or less familiar fractions.

Fourth, the sample consists entirely of participants who hold a bachelor's degree in science. This is mentioned in the methods but never discussed as a limitation. These individuals are at the high end of math education, which once again limits the ability of the paper to make broad claims about how "education" generally expands the number concept and how cultural inventions like fractions become integrated into the neural number system. Demonstrating this would require comparing individuals with different levels of mathematical training, which is not done here.

Overall, the issues highlighted above mean that the conclusions of the paper rest on a very specific set of conditions (i.e., a particular task requiring explicit magnitude comparison, a small set of simple fractions, and a highly selected sample). The discrepancy between these conditions and the broad claims made throughout the title, abstract, and discussion is, in my view, too large. I think the findings are more accurately described as demonstrating that when educated adults are engaged in explicit magnitude comparison, the brain can construct an ordered representation that integrates fractions and integers, with a topographic organization in the anterior IPS/IPL. This is a meaningful contribution, particularly the numerotopic mapping for symbolic numbers, which is genuinely novel. But it is a finding about processing magnitudes under some task demands, not about the stable semantic organization of numerical knowledge, and the paper should be framed accordingly.

COMMENT #2

I also have some methodological issues with the paper. One concerns the definition of the mask used to constrain most fMRI analyses. If I understand correctly, the math-responsive mask was defined by taking the union of individual-level activation maps, each thresholded at p < 0.001 uncorrected. This means that a voxel that survives the threshold in only one participant (out of 20) is included in the group mask. Unlike a standard one-sample t-test at the group level, this is not a valid group-level inference and this means that the resulting mask is almost certainly very large and includes voxels that are not reliably math-responsive at the group (let alone at the individual) level. This has downstream consequences for the searchlight RSA (which is conducted within this mask, partly defeating the purpose of a searchlight approach), for the RSA and MDS within "math-responsive areas" (where noisy voxels could dilute or distort the signal), and for the Gaussian voxel-tuning analysis (where fitting tuning curves to non-math voxels may add noise). At minimum, the authors should use a proper group-level contrast (e.g., one-sample t-test, ideally corrected for multiple comparisons) to define the search space. Otherwise, there is not much point localizing brain areas with so little specificity across participants.

COMMENT #3

Given the absence of preregistration and the large number of statistical tests conducted across multiple analyses and brain regions, I am concerned about the cumulative risk of false positives. I am not asking for correction across all analyses in the paper. However, I do think that tests within a given analysis should be appropriately corrected. This is particularly relevant for the voxel-tuning correlation analyses, where correlations between preferred numbers are reported separately for all math-responsive areas, both within-domain (across runs) and cross-domain (fractions vs. integers). This amounts to a substantial number of tests, each assessed by a separate permutation test with (I believe) no correction for the number of regions tested. The same concern applies to the RSA results reported in Table S1, where the effect of fraction-to-integer distance is tested separately in six ROIs (I don't think there is a correction for multiple comparisons here).

COMMENT #4

Several analytical choices in the voxel-tuning analysis should be better justified. The R² threshold is set at 0.5 at the group level and 0.3 at the individual level, but no rationale is provided for these values. The smoothing kernel differs between analyses (4 mm for the tuning analysis, 6 mm for other analyses). The top 1% of voxels was selected for PCA visualization. As the study was not preregistered, these choices could influence the results. The authors should either justify these choices explicitly or demonstrate that the key findings are stable across a range of reasonable parameter values.

COMMENT #5

The discussion of hippocampal findings is fairly speculative relative to the strength of the evidence. The Gaussian tuning analysis shows no reliable tuning for integers in the hippocampus and only marginal effects for fractions. The cross-domain correlation in the hippocampus is not reported separately, and it is unclear whether the hippocampal RSA effects survive the multiple comparison concerns raised above. Yet the discussion devotes substantial space to interpreting hippocampal involvement, and even suggests that hippocampal engagement reflects fractions being a "challenging domain" (p. 27) for adults. This is an interesting hypothesis, but it is entirely post-hoc and unsupported by any analysis relating individual differences in fraction competence to hippocampal activation. I think the authors should be more cautious in their interpretation of the hippocampal findings, or provide supporting analyses.

COMMENT #6

Finally, the data availability statement indicates that code and figure outputs are shared on OSF, but this has limited use if the fMRI data themselves are not also openly available in a public repository. I understand that sharing raw MRI data may be a concern, but I do not think anything prevents the authors from sharing processed maps and would certainly encourage them to do so.

Reviewer #2: This manuscript describes an empirical 7T fMRI study aiming to understand how the meaning of abstract symbols is represented throughout the human brain. To do so, the authors use an established behavioural paradigm (continuous judgment magnitude comparison task) to measure how BOLD responses to integers and fractions differ across brain regions known to be associated with mathematical thought as well as other plausible networks of association. The integration of both integer and fraction responses into a "joint semantic map" is taken to suggest that the process of enculturation/education leads to a converging of cortical representations that would presumably support shared decision-making and more abstracted mathematical thought across formats. While I have a number of concerns with the current version of the manuscript, I hope these can be straightforwardly addressed with minor revisions.

Given the subject matter, I would have expected some engagement with the distinction between quantical (e.g., quantity discrimination) and numerical (exact, symbolic) cognition, following Núñez (2017) etc. Also, some greater consideration of the need to distinguish between numerosity, numerousness, and number language terms following Dos Santos (2022) would seem warranted given the nature of the claims being made about representations of integers and fractions.

Núñez, R. E. (2017). Is there really an evolved capacity for number?. Trends in cognitive sciences, 21(6), 409-424.

César Frederico Dos Santos (2022) Re-establishing the distinction between numerosity, numerousness, and number in numerical cognition, Philosophical Psychology, 35:8, 1152-1180, DOI: 10.1080/09515089.2022.2029387

The framing of conceptual spaces or "semantic maps" following Gardenfors (2000) is brought up initially but is not applied in any meaningful way as far as I have read Gardenfors; e.g., tests of convexity, any consideration of what would be a "prototypical" integer or fraction to be used as stimuli. MDS is potentially related to the concept of conceptual spaces but otherwise there is little engagement with this theoretical work.

There is also mention of "gradients of neuronal activation on the surface of the cortex" (line 50) and "map-like organization" (lines 52-53) in the Introduction without reference to most of the work by Ben Harvey and colleagues on non-symbolic topographic maps of numerosity that would seem very pertinent here, even if not strictly using symbols.

Line 57-59: "…the cerebral representation of numerical symbols, including intuitive ones - such as the integers 0-5 - as well as more abstract forms which are acquired through education - fractions such as 1/2, 1/3, or 5/2."

What is exactly intuitive about integer symbols, are they any more intuitive than fraction symbols? Visually simpler perhaps but not necessarily more intuitive. In fact, a case could be made that fraction symbols are more intuitive - a consistent operator is shown as the middle symbol and the numerator and dominator indicate a part/whole relationship. By contrast, individual integer symbols (Indo-Arabic) offer no information about their relationship to quantity or any other mathematical identity. Fractions or rational numbers may signifier more abstract concepts but the point being made is about "the cerebral representation of numerical symbols".

Line 83: You state here that "This process is typically attributed to the Approximate Number System (ANS)" but see the work of Attila Krajcsi on the discrete semantic system (DSS). Some attention to this alternative perspective would seem necessary.

Krajcsi, A. Numerical distance and size effects dissociate in Indo-Arabic number comparison. Psychon Bull Rev 24, 927-934 (2017). https://doi.org/10.3758/s13423-016-1175-6

Krajcsi, A., Kojouharova, P., Lengyel, G. (2022). Processing Symbolic Numbers: The Example of Distance and Size Effects. In: Gervain, J., Csibra, G., Kovács, K. (eds) A Life in Cognition. Language, Cognition, and Mind, vol 11. Springer, Cham. https://doi.org/10.1007/978-3-030-66175-5_27

Krajcsi, A., Fedele, M., & Reynvoet, B. (2023). The approximate number system cannot be the leading factor in the acquisition of the first symbolic numbers. Cognitive Development, 65, 101285.

Krajcsi, A., Kojouharova, P. Different sources of the numerical comparison size effect. Mem Cogn (2026). https://doi.org/10.3758/s13421-025-01781-2

Line 337: Is an efficiency score like the rate correct score (Accuracy / RTs) the best way to measure performance here? Would a balanced integration score and/or some other measure work better? Does the choice of measure effect the MDS result and can you rule out this possibility?

Lines 387-391: Related to the last point, you interpret the two-dimensional MDS scaling qualitatively in terms of magnitudes on the first dimension (x axis), and difficulty on the second dimension (y axis). Is there a quantitative metric to support these observations? For instance, is this scaling pattern different from random arrangement and how different would it need to be for you to retain the current interpretation? Looking at Figure 1E it seems numerosity 2 and 3 are aligned in difficulty (axis 1) - what theory or model of numerical cognition would support that idea?

Lines 390-391: On the comment that "[the second dimension] separated the intrinsically easier numbers 0 and 5 (because they lie at the extremes) from other numbers". You cite work from Dehaene & Changeux (1993) as well as more recently Paul, van Ackooij, ten Cate & Harvey (2022) who highlight monotonically increasing (5) and monotonically decreasing (0) responses may explain responses at both ends of the range amongst tuned responses - could the distinction shown in the second dimension (y axis) instead reflect this possibility, how would you rule this out?

Lines 486-530: I had trouble following the descriptions in text and the relevant figure panels in this section. Perhaps it was me, but it seems the text incorrectly refers to panels of Figure 7 and Figure S2 throughout.

Line 496: what "statistical test"? Same for the results of the next paragraph.

Lines 531-535: I understand the four best participants are shown in Figure S3, but could you compute the topographic progression along the cortical surface to quantify this result as typically done; see Harvey & Dumoulin (2017), Figure 2.

Graphical comment: Figure 3 does not seem to have an associated colourmap?

Academic Editor:

In addition to the points already raised by the reviewers, I would encourage the authors to consider the following:

Reviewer 1 articulates well the concern that the manuscript focuses primarily on similarities, with insufficient consideration of their interpretation or underlying causes. There is a substantial body of literature addressing precisely these issues. In particular, the target article by Cohen Kadosh and Walsh (2009, Behavioral and Brain Sciences), as well as earlier work such as Bulthé et al (2014, NeuroImage, https://doi.org/10.1016/j.neuroimage.2013.10.049), Cohen Kadosh et al. (2007, Neuron), Liu et al (2011, PLOS One, https://doi.org/10.1371/journal.pone.0019373) and Sokolowski et al (2017, NeuroImage, https://doi.org/10.1016/j.neuroimage.2016.10.028) highlight important conceptual and methodological caveats directly relevant to the authors’ approach. These works, among others, are not cited. The manuscript would benefit from a more balanced and cautious perspective, including consideration of analyses to examine potential differences in representations. At present, it misses an opportunity to situate the findings within a broader framework in which multiple representations, potentially including both shared and distinct components shaped by task demands, are considered.

The choice to compare natural fractions (up to 2.5) with integers (up to 5), rather than with decimal fractions of equivalent magnitude, requires stronger justification. In a psychology journal, this caveat would have triggered a rejection.

Line 385: It is unclear why the degrees of freedom are reported as 7. It would be helpful to clarify whether the analysis was conducted on data averaged across participants, rather than modelling at the individual level. Ideally, the authors should demonstrate at the participant level that the logarithmic function provides the best fit.

The rationale for dividing accuracy by response times is not convincing, particularly given that this approach has been widely criticised. A more appropriate and informative approach would be to apply diffusion modelling and compare drift rates, which would allow a more direct assessment of information processing.

Line 493: The reported correlation (r = 0.01, p = 0.008) is extremely small. The degrees of freedom should be reported here, as well as consistently across the manuscript alongside other statistical details (e.g., t values).

# Author comment: Author response to Decision Letter 2

**DOI:** 10.1371/journal.pbio.3003820.r003

16 Jul 2026

Attachment Submitted filename: response_reviewers_1507.docx

# Peer review: Decision Letter 2

**DOI:** 10.1371/journal.pbio.3003820.r004

**Authors:** Christian Schnell, PhD

14 Aug 2026

Dear Dr Valério,

Thank you for your patience while we considered your revised manuscript "A cortical semantic space integrating fractions and integers" for publication as a Research Article at PLOS Biology. This revised version of your manuscript has been evaluated by the PLOS Biology editors, the Academic Editor and the original reviewers.

Based on the reviews and on our Academic Editor's assessment of your revision, we are likely to accept this manuscript for publication, provided you satisfactorily address the remaining comments from the Academic Editor. Please also make sure to address the following data and other policy-related requests:

* We would like to suggest a different title to improve its accessibility for our broad audience:

Integers and fractions are integrated in a joint semantic map in the human parietal cortex

* Please add the links to the funding agencies in the Financial Disclosure statement in the manuscript details.

* DATA POLICY:

You may be aware of the PLOS Data Policy, which requires that all data be made available without restriction: http://journals.plos.org/plosbiology/s/data-availability. For more information, please also see this editorial: http://dx.doi.org/10.1371/journal.pbio.1001797

Note that we do not require all raw data. Rather, we ask that all individual quantitative observations that underlie the data summarized in the figures and results of your paper be made available in one of the following forms:

1) Supplementary files (e.g., excel). Please ensure that all data files are uploaded as 'Supporting Information' and are invariably referred to (in the manuscript, figure legends, and the Description field when uploading your files) using the following format verbatim: S1 Data, S2 Data, etc. Multiple panels of a single or even several figures can be included as multiple sheets in one excel file that is saved using exactly the following convention: S1_Data.xlsx (using an underscore).

2) Deposition in a publicly available repository. Please also provide the accession code or a reviewer link so that we may view your data before publication.

Regardless of the method selected, please ensure that you provide the individual numerical values that underlie the summary data displayed in the following figure panels as they are essential for readers to assess your analysis and to reproduce it: 1BC, 7AB, S7C and S8D.

NOTE: the numerical data provided should include all replicates AND the way in which the plotted mean and errors were derived (it should not present only the mean/average values).

Please also ensure that figure legends in your manuscript include information on where the underlying data can be found, and ensure your supplemental data file/s has a legend.

Please ensure that your Data Statement in the submission system accurately describes where your data can be found.

* CODE POLICY

Per journal policy, if you have generated any custom code during the course of this investigation, please make it available without restrictions. Please ensure that the code is sufficiently well documented and reusable, and that your Data Statement in the Editorial Manager submission system accurately describes where your code can be found. More information on our Code Policy, what and how to share can be found here: https://journals.plos.org/plosbiology/s/code-availability

Please note that we cannot accept sole deposition of code in GitHub, as this could be changed after publication. However, you can archive this version of your publicly available GitHub code to Zenodo. Once you do this, it will generate a DOI number, which you will need to provide in the Data Accessibility Statement (you are welcome to also provide the GitHub access information). See the process for doing this here: https://docs.github.com/en/repositories/archiving-a-github-repository/referencing-and-citing-content

As you address these items, please take this last chance to review your reference list to ensure that it is complete and correct. If you have cited papers that have been retracted, please include the rationale for doing so in the manuscript text, or remove these references and replace them with relevant current references. Any changes to the reference list should be mentioned in the cover letter that accompanies your revised manuscript.

In addition to these revisions, you may need to complete some formatting changes, which you will receive in a follow up email. A member of our team will be in touch with a set of requests shortly. If you do not receive a separate email within a few days, please assume that checks have been completed, and no additional changes are required.

We expect to receive your revised manuscript within two weeks.

To submit your revision, please go to https://www.editorialmanager.com/pbiology/ and log in as an Author. Click the link labelled 'Submissions Needing Revision' to find your submission record. Your revised submission must include the following:

- a cover letter that should detail your responses to any editorial requests, if applicable, and whether changes have been made to the reference list

- a Response to Reviewers file that provides a detailed response to the reviewers' comments (if applicable, if not applicable please do not delete your existing 'Response to Reviewers' file.)

- a track-changes file indicating any changes that you have made to the manuscript.

NOTE: If Supporting Information files are included with your article, note that these are not copyedited and will be published as they are submitted. Please ensure that these files are legible and of high quality (at least 300 dpi) in an easily accessible file format. For this reason, please be aware that any references listed in an SI file will not be indexed. For more information, see our Supporting Information guidelines:

https://journals.plos.org/plosbiology/s/supporting-information

*Published Peer Review History*

Please note that you may have the opportunity to make the peer review history publicly available. The record will include editor decision letters (with reviews) and your responses to reviewer comments. If eligible, we will contact you to opt in or out. Please see here for more details:

https://plos.org/published-peer-review-history/

*Press*

Should you, your institution's press office or the journal office choose to press release your paper, please ensure you have opted out of Early Article Posting on the submission form. We ask that you notify us as soon as possible if you or your institution is planning to press release the article.

*Protocols deposition*

To enhance the reproducibility of your results, we recommend that if applicable you deposit your laboratory protocols in protocols.io, where a protocol can be assigned its own identifier (DOI) such that it can be cited independently in the future. Additionally, PLOS ONE offers an option for publishing peer-reviewed Lab Protocol articles, which describe protocols hosted on protocols.io. Read more information on sharing protocols at https://plos.org/protocols?utm_medium=editorial-email&utm_source=authorletters&utm_campaign=protocols

Please do not hesitate to contact me should you have any questions.

Sincerely,

Christian

Christian Schnell, PhD

Senior Editor

cschnell@plos.org

PLOS Biology

------------------------------------------------------------------------

Reviewer remarks:

Reviewer #1: I would like to thank the authors for having made a genuine effort to address the concerns I raised in the initial review. In particular, the reframing of the claims throughout the abstract, introduction, and discussion now accurately reflects what the data can support, i.e., that during an active magnitude comparison task, the brain constructs an integrated, topographically organized representation of fractions and integers in the IPS. The additional analyses, especially the within-domain trials analysis and the PCA with matched magnitude ranges, strengthen the empirical contribution. I am satisfied with the revised paper, which I believe makes a novel contribution to the literature.

Reviewer #2: I appreciate the authors have engaged thoroughly with my previous comments and have addressed all of my previously stated concerns:

1. Sufficient clarification in text

2. Resolved concept of "intuitive"

3. Alternative explanation now included

4. I'm glad to see use of the BIS now, which is a more robust measure and seems to provide clearer results than ES (albeit they often agree anyway)

5. Quantitative tests now provide stronger grounding for interpretation of dimensions

6. Alternative explanation now provided

7. Figure/text update can now be followed

8. Clarification of statistical tests easier to follow

9. Individual differences and group trends in orderly topographic progression are now present for readers to interpret

10. Figure now more interpretable

I have no further outstanding concerns and can recommend acceptance.

Comments from the Academic Editor:

1. Lines 746–747: “However, other accounts suggest that, depending on the task goal, different types of number representations may be created (Cohen Kadosh & Walsh, 2009).” This does not accurately represent the cited account. Specifically, the paper proposed that multiple representations exist by default, for example for fractions, integers and non-symbolic quantities, whereas a shared representation may be created when explicitly required by the task. This interpretation actually supports Reviewer 1’s suggestion and would appropriately temper the authors’ stronger claim.

2. Line 157: “This relatively small and fully balanced stimulus set facilitates interpretable results in MDS and RSA…” I would avoid describing the stimulus set as “fully balanced”, given the acknowledged concern regarding the unmatched stimuli. The authors have addressed this caveat well through the additional analyses, but calling the set fully balanced would be an overstatement.

3. Linear mixed-effects model: Could the authors please clarify the random-effects structure, including how participant, trial type and number were modelled, and whether trial type and number were treated as fixed or random effects?

4. The reported correlation is extremely small but nevertheless statistically significant (r=.01). I can understand how this might arise given the number of observations, but it would be worth confirming that the value is correct and not a typographical or analytical error.

# Author comment: Author response to Decision Letter 3

**DOI:** 10.1371/journal.pbio.3003820.r005

15 Sep 2026

Attachment Submitted filename: response_reviewers_2ndround_sd.docx

# Editor report: Decision Letter 3

**DOI:** 10.1371/journal.pbio.3003820.r006

**Authors:** Christian Schnell, PhD

18 Sep 2026

Dear Dr Valério,

Thank you for the submission of your revised Research Article "A cortical semantic map integrates fractions and integers in the human brain" for publication in PLOS Biology. On behalf of my colleagues and the Academic Editor, Roi Cohen Kadosh, I am pleased to say that we can in principle accept your manuscript for publication, provided you address any remaining formatting and reporting issues. These will be detailed in an email you should receive within 2-3 business days from our colleagues in the journal operations team; no action is required from you until then. Please note that we will not be able to formally accept your manuscript and schedule it for publication until you have completed any requested changes.

Please take a minute to log into Editorial Manager at http://www.editorialmanager.com/pbiology/, click the "Update My Information" link at the top of the page, and update your user information to ensure an efficient production process.

PRESS

We frequently collaborate with press offices. If your institution or institutions have a press office, please notify them about your upcoming paper at this point, to enable them to help maximize its impact. If the press office is planning to promote your findings, we would be grateful if they could coordinate with biologypress@plos.org. If you have previously opted in to the early version process, we ask that you notify us immediately of any press plans so that we may opt out on your behalf.

We also ask that you take this opportunity to read our Embargo Policy regarding the discussion, promotion and media coverage of work that is yet to be published by PLOS. As your manuscript is not yet published, it is bound by the conditions of our Embargo Policy. Please be aware that this policy is in place both to ensure that any press coverage of your article is fully substantiated and to provide a direct link between such coverage and the published work. For full details of our Embargo Policy, please visit http://www.plos.org/about/media-inquiries/embargo-policy/.

Thank you again for choosing PLOS Biology for publication and supporting Open Access publishing. We look forward to publishing your study.

Sincerely,

Christian

Christian Schnell, PhD

Senior Editor

PLOS Biology

cschnell@plos.org
